# Supplementary material for: Investigating grey matter volumetric trajectories through the lifespan at the individual level
Source: Nat Commun. 2024 Jul 15;15:5954. doi: 10.1038/s41467-024-50305-0 (PMC11251262; doi:10.1038/s41467-024-50305-0)
Supplement: Supplementary file 1 — Supplementary Information [file 41467_2024_50305_MOESM1_ESM.pdf]

Supplementary Information

Investigating grey matter volumetric trajectories through the lifespan at the individual level

Runye Shi, Shitong Xiang, Tianye Jia, Trevor W. Robbins, Jujiao Kang, Tobias Banaschewski, Gareth J. Barker, Arun L.W. Bokde, Sylvane Desrivières, Herta Flor,, Antoine Grigis, Hugh Garavan, Penny Gowland, Andreas Heinz, Rüdiger Brühl, Jean-Luc Martinot, Marie-Laure Paillère Martinot, Eric Artiges, Frauke Nees, Dimitri Papadopoulos Orfanos, Tomáš Paus, Luise Poustka, Sarah Hohmann, Sabina Millenet, Juliane H. Fröhner, Michael N. Smolka, Nilakshi Vaidya, Henrik Walter, Robert Whelan, Gunter Schumann, Xiaolei Lin*, Barbara J. Sahakian*, Jianfeng Feng* and IMAGEN Consortium

Contents

Supplementary Fig. 1. The joint distribution of baseline total GMV and GMV developmental trajectories. ...4
Supplementary Fig. 2. The first two PCA components. Principal component analysis was used to define a low-dimensional representation of GMV developmental patterns. ...5
Supplementary Fig. 3. The Elbow Method showing the optimal k. ...6
Supplementary Fig. 4. Total GMV developmental curves of groups from 14y to 23y. ...7
Supplementary Fig. 5. Estimated total GMV developmental curves of individuals in groups from 5y to 37y...8
Supplementary Fig. 6. Estimated total GMV developmental curves of groups from 5y to 37y. ...9
Supplementary Fig. 7. Neurocognition comparisons of Group 3 and Group 2 vs Group 1 at baseline and last follow-up. ...10
Supplementary Fig. 8. Correlation between longitudinal trajectory of GMV in each cortical ROI and neurocognition in groups ...11
Supplementary Fig. 9. Locus zoom of the top SNP rs9375442 on chromosome 6. ...12
Supplementary Fig. 10. Associations between SNPs on CENPW and cortical surface area (CSA) and brain volume (BV) in existing studies. ...13
Supplementary Fig. 11. Gene-based GWAS results for delayed neurodevelopment. ...14
Supplementary Fig. 12. Validation of GWAS results for delayed neurodevelopment in IMAGEN. ...15
Supplementary Fig. 13. GWAS results and validation for Group 2 vs Group 1. ...16
Supplementary Fig. 14. EWAS Manhattan plot in the IMAGEN population comparing Group 3 with Group 1 and Group 2. ...17
Supplementary Fig. 16. Correlations between CENPW score and socio-economics, cognitive and mental health and in UKB. ...19
Supplementary Fig. 17. The top 5 discriminating ROIs with largest t values comparing the GMV trajectories between Group 3 and Group 2. ...20
Supplementary Fig. 18. Polygenic risk scores (PGS) used to predict ADHD, ASD ad IQ in IMAGEN ...21
Supplementary Fig. 19. Miami plot of GWAS for Group 3 vs Group 1 and Group 2. ...22
Supplementary Fig. 20. Q-Q plots of GWAS for Group 3 vs Group 1 and Group 2. ...23
Supplementary Fig. 21. Q-Q plots of GWAS for Group 3 vs Group 1/2. ...24
Supplementary Fig. 22. Polygenic risk scores (PGS) used to distinguish Group 3 from Group 1/2 and Group 2 from Group 1 in IMAGEN. ...25
Supplementary Table 1. Rotation matrix, factor loading and proportion of variance explained by each PCA component. ...26
Supplementary Table 2. Full demographics and baseline characteristics for Group 1, Group 2 and Group 3 in IMAGEN. ...30
Supplementary Table 3. t-map and d-map of the top 20 distinct brain regions in the group clustering in IMAGEN. ...33
Supplementary Table 4. Full comparison of Group 2 and Group 3 vs Group 1 in terms of the personal traits, environmental burden, neuro-cognition, behavioral risk factors and mental symptoms at age 14, longitudinal trajectory and at age 23 ...34
Supplementary Table 5. The longitudinal correlation between cortical GMVs and neurocognition functions for Group 3. ...40
Supplementary Table 6. The longitudinal correlation between cortical GMVs and neurocognition functions

for Group 2. .... **42**
Supplementary Table 7. The overlapping number of participants between clustering results within different sex and the one within the whole population **44**
Supplementary Table 8. Full comparison of Group 2 and Group 3 vs Group 1 clustered within sex in terms of the personal traits, environmental burden, neuro-cognition, behavioral risk factors and mental symptoms at age 14, longitudinal trajectory and at age 23. .... **45**
Supplementary Table 9. Comparison of PRS for attention-deficit/hyperactivity disorder (ADHD), austism disorder (ASD), educational attainment (EA) and IQ among three groups. **51**
Supplementary Table 10. Correlation between Group3-reweighted GMV and neurocognition in ABCD. **52**
Supplementary Table 11. SNPs with significant genome-wise significant effects in Group 3 GWAS. **53**
Supplementary Table 12. Mediation effect of different environmental factors on peak GMV through cg06064461 in IMAGEN. **55**
Supplementary Table 13. Correlations between PRS of delayed neurodevelopment and baseline brain region GMVs in UKB. **56**
Supplementary Table 14. Correlations between CENPW score of delayed neurodevelopment and baseline brain region GMVs in UKB. **58**
Supplementary Table 15. Correlations between genetic risk score of delayed neurodevelopment and neurocognition in UKB **60**
Supplementary Table 16. Demographics and baseline characteristics for all adolescents in IMAGEN and those included in the analyses. **61**
Supplementary Table 17. Demographics and baseline characteristics for participants in ABCD, IMAGEN and UKB study. **63**
Supplementary Methods **65**
ABCD - Adolescent Brain and Cognitive Development 65
Structural neuroimaging data. 65
Genetic data 65
IMAGEN 65
Structural neuroimaging data 66
Genetic data and epigenetic data 66
HCP - Human Connectome Project 67
Structural neuroimaging data 67
PNC - Philadelphia Neuroimaging Cohort 67
Structural neuroimaging data 67
UKB - UK Biobank 68
Structural neuroimaging data 68
Genetic data 68
Supplementary Table 18. A sketch of data utilization in analysis. 68
Supplementary Table 19. Neurocognition assessments in ABCD 68
Supplementary Table 20. Environmental, neurocognition, behavioral, personal trait and mental health assessments in IMAGEN 69
Supplementary Table 21. Socioeconomic, cognition and mental health assessments in UKB 74
Supplementary References **76**

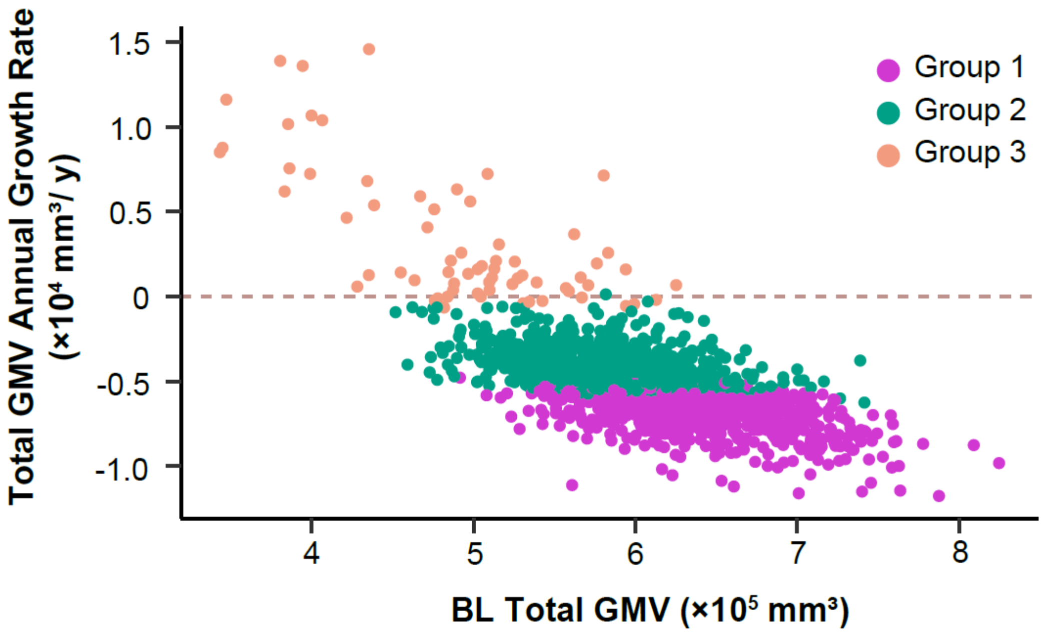

Supplementary Fig. 1. The joint distribution of baseline total GMV and GMV developmental trajectories. Individuals in IMAGEN (n = 1,543) exhibit remarkable heterogeneity in baseline (BL) total GMV and annual growth rate of total GMV from baseline to follow-ups.

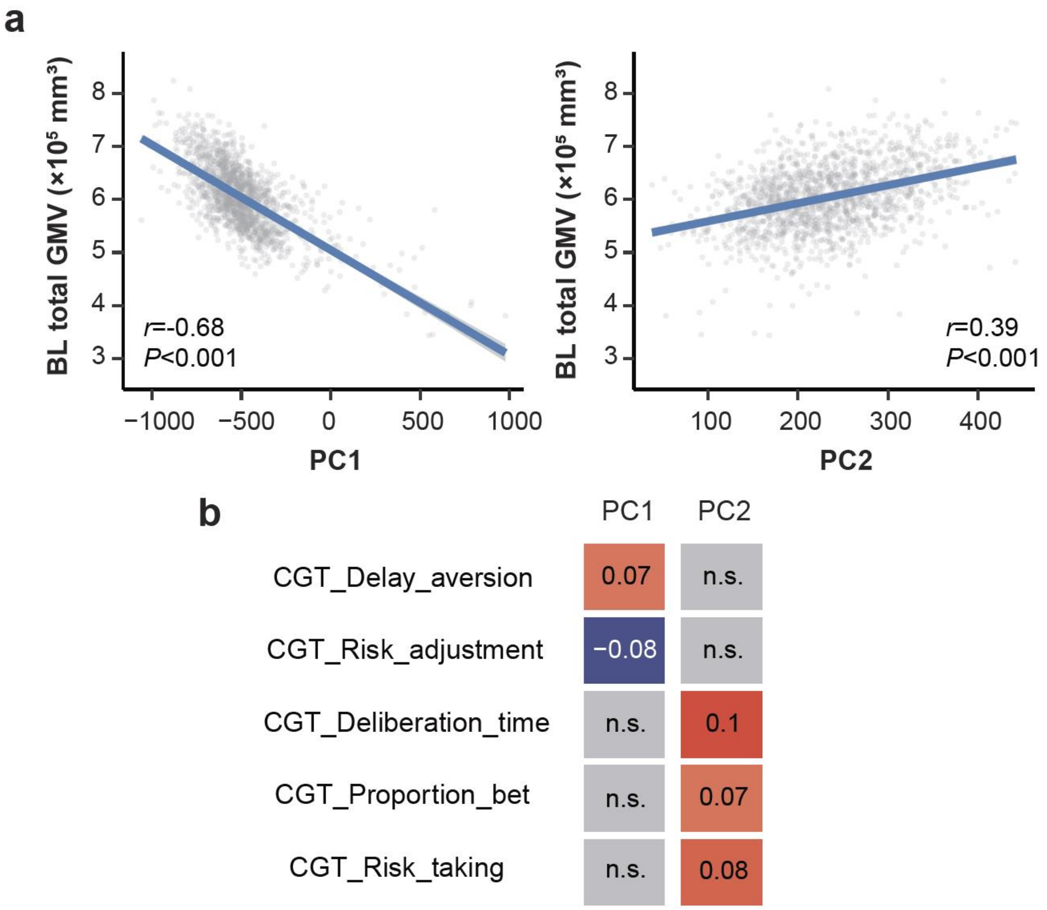

Supplementary Fig. 2. The first two PCA components. Principal component analysis was used to define a low-dimensional representation of GMV developmental patterns.

The first and second PCs were significantly associated with baseline total GMV **(a)** (*n* = 1,543) while they exhibited different association patterns with executive functions in the Cambridge Gambling Task (CGT) **(b)**. **(b)** illustrates the correlations between PCs and CGT performances (n.s. = not significant).

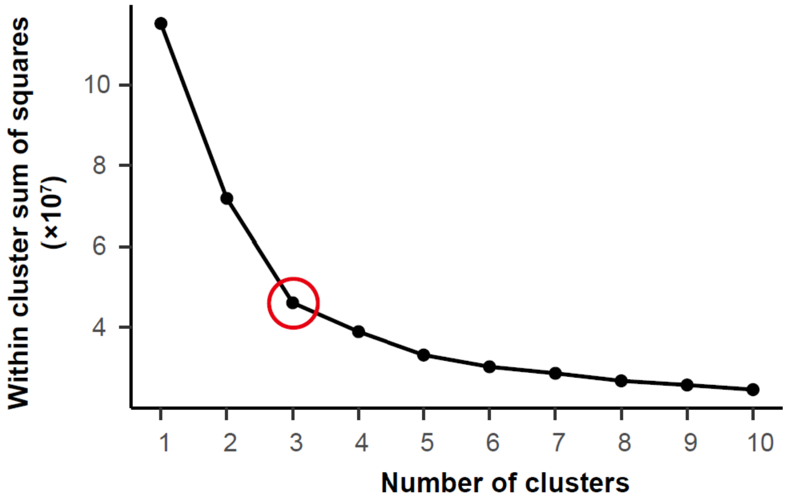

Supplementary Fig. 3. The Elbow Method showing the optimal k.

Considering the distribution of population among groups, 3 was chosen as the optimal number of clusters.

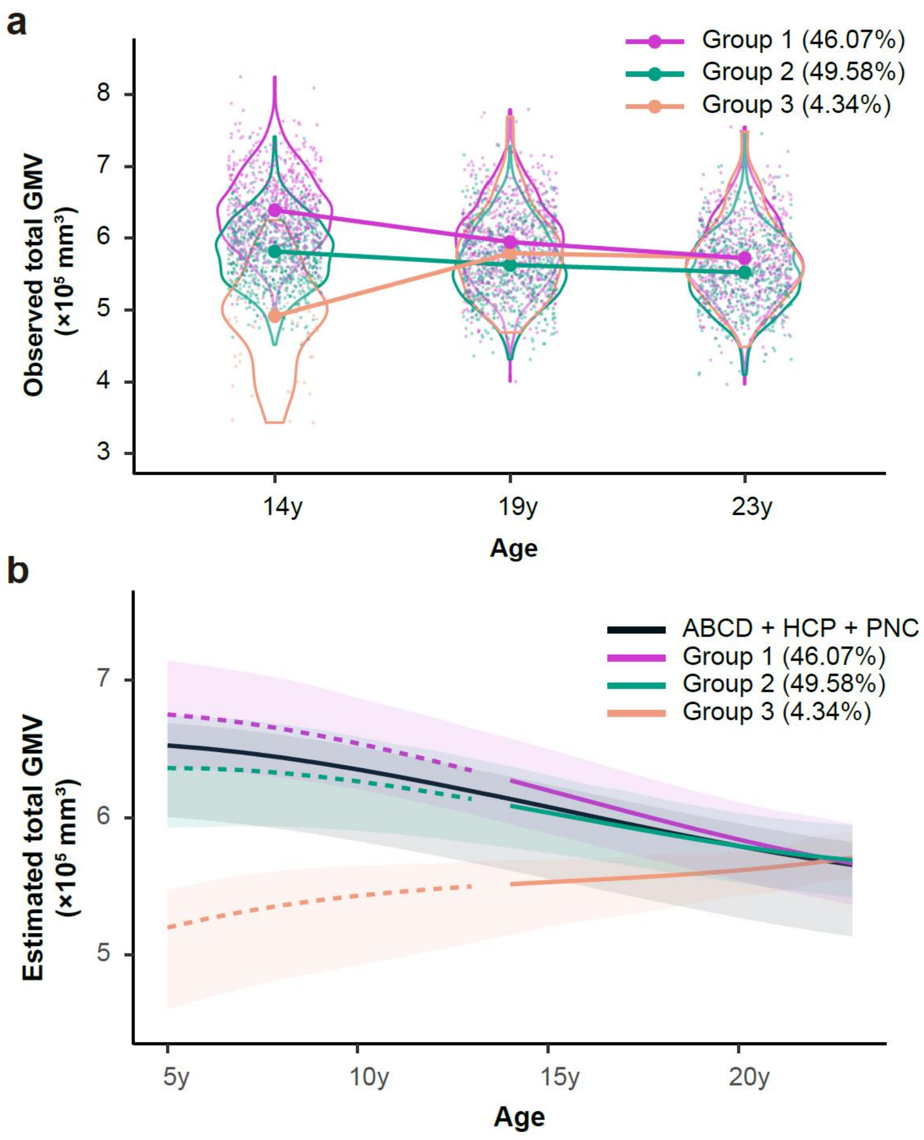

Supplementary Fig. 4. Total GMV developmental curves of groups from 14y to 23y.

(a) Observed total GMV developmental curves of groups from 14y to 23y (n = 711 for Group 1, n = 765 for Group 2, n = 67 for Group 3). **(b)** Estimated total GMV developmental curves (with 95% confidence bands) for groups and reference population (ABCD+HCP+PNC, n = 21,826). These curves were estimated adjusting for sex, site/scanner, handedness and intra-cranial volume (**Methods**). Group 1 and 2 exhibited similar GMV developmental trend with the reference population, while Group 3 had opposite GMV developmental trend.

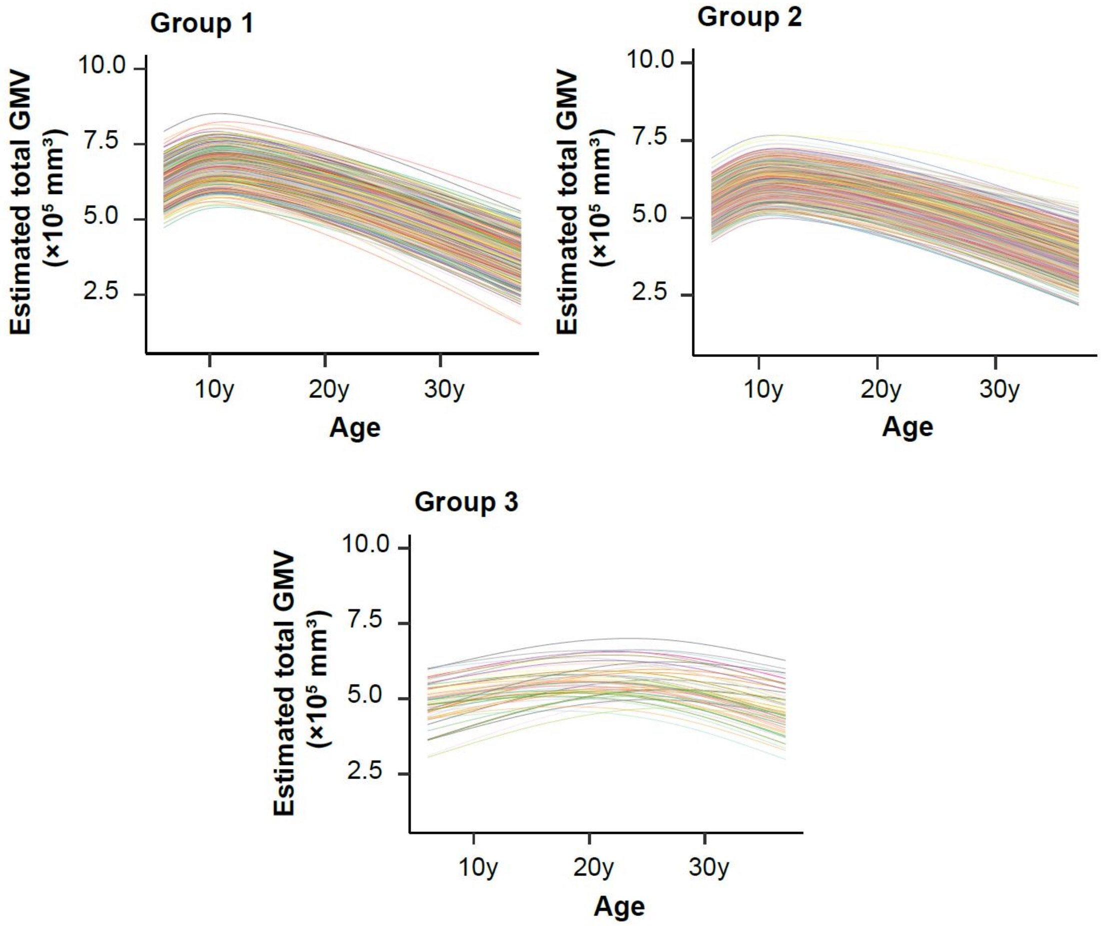

Supplementary Fig. 5. Estimated total GMV developmental curves of individuals in groups from 5y to 37y. Total GMV developmental trajectories for individuals in three groups were estimated using linear mixed effect model with B spline function of age (**Methods**).

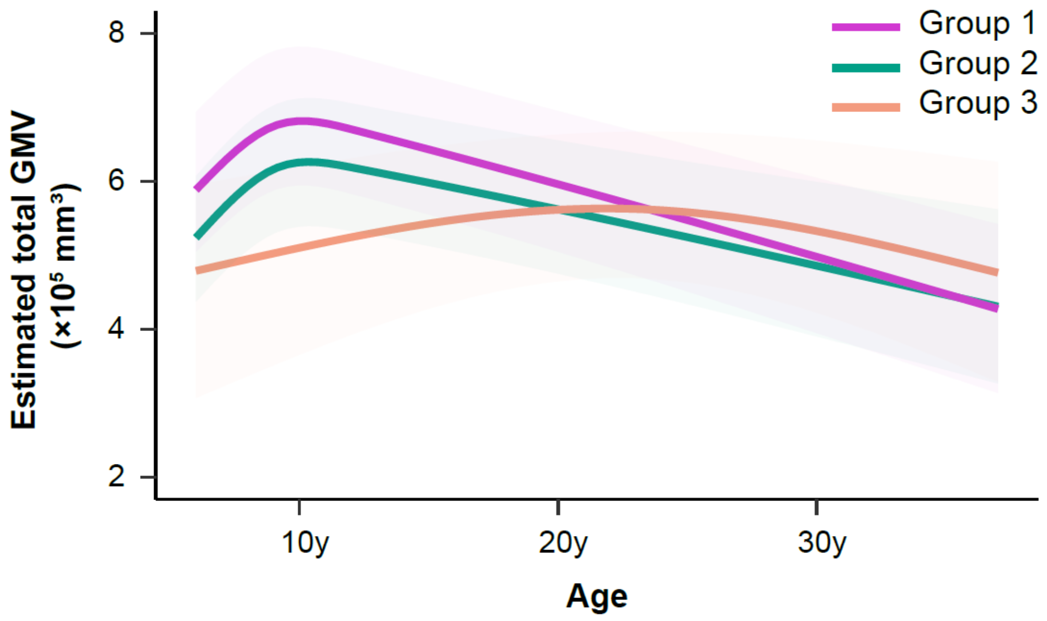

Supplementary Fig. 6. Estimated total GMV developmental curves of groups from 5y to 37y.
Mean total GMV developmental trajectories (with 95% confidence bands) for groups were plotted using estimated individual GMV trajectories in **Supplementary Fig. 5**. Ranges from the 2.5th percentile to the 97.5th percentile of the corresponding group were plotted as bands.

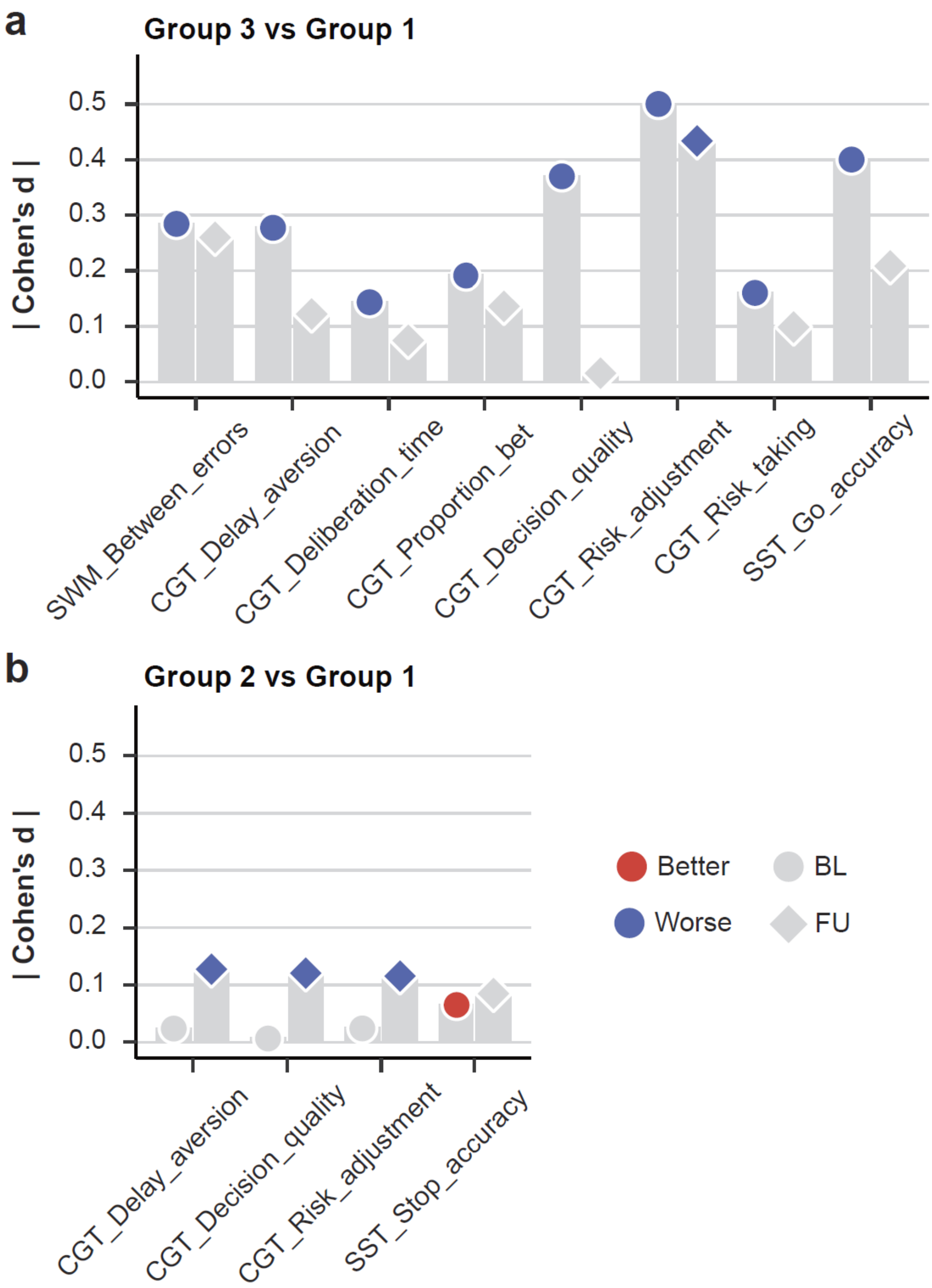

Supplementary Fig. 7. Neurocognition comparisons of Group 3 and Group 2 vs Group 1 at baseline and last follow-up.

Neurocognition profiles of Group 3 **(a)** and Group 2 **(b)** at baseline (14y) and last follow-up (23y). Comparisons of neurocognitive performances were converted to Cohen's d and item-specific neurocognitive comparisons reflect better or worse performances relative to Group 1. Gray color indicated not significant results. SST comparisons were not included in the count in **Fig. 2a**.

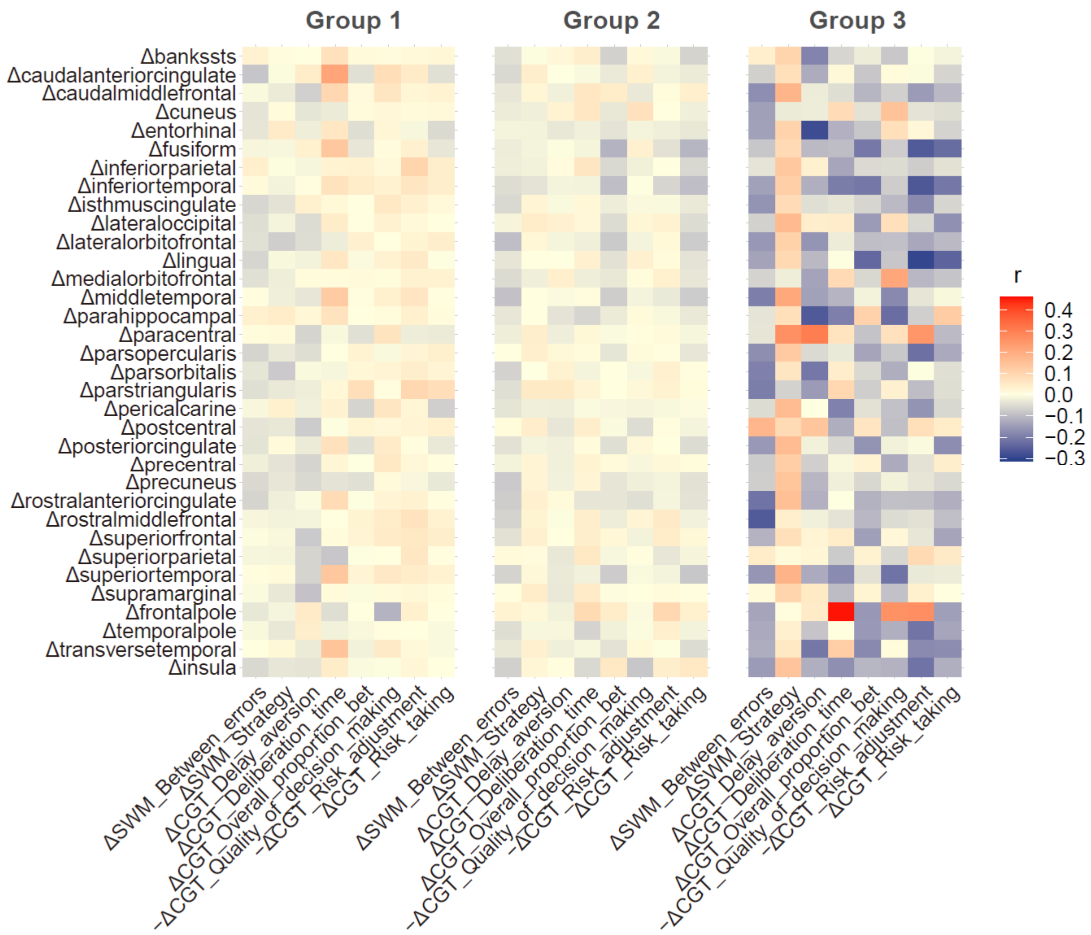

Supplementary Fig. 8. Correlation between longitudinal trajectory of GMV in each cortical ROI and neurocognition in groups.

Negative correlations between the developmental trajectories of GMV in the top discriminating ROIs and neurocognitive performances for Group 2 (n = 765) were observed while increasing GMV in the top discriminating ROIs showed positive correlation with improvements of neurocognitive functions for Group 3 (n = 67).

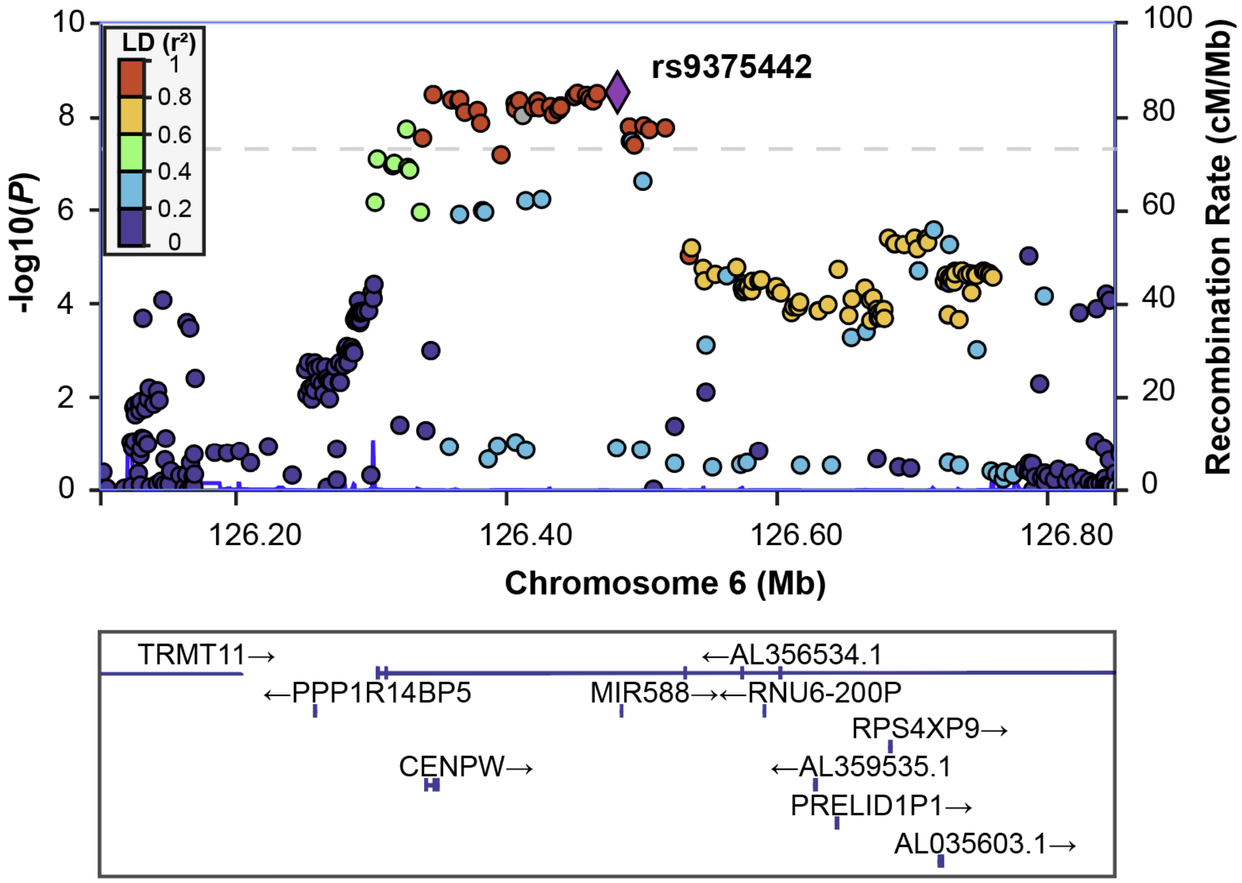

Supplementary Fig. 9. Locus zoom of the top SNP rs9375442 on chromosome 6.

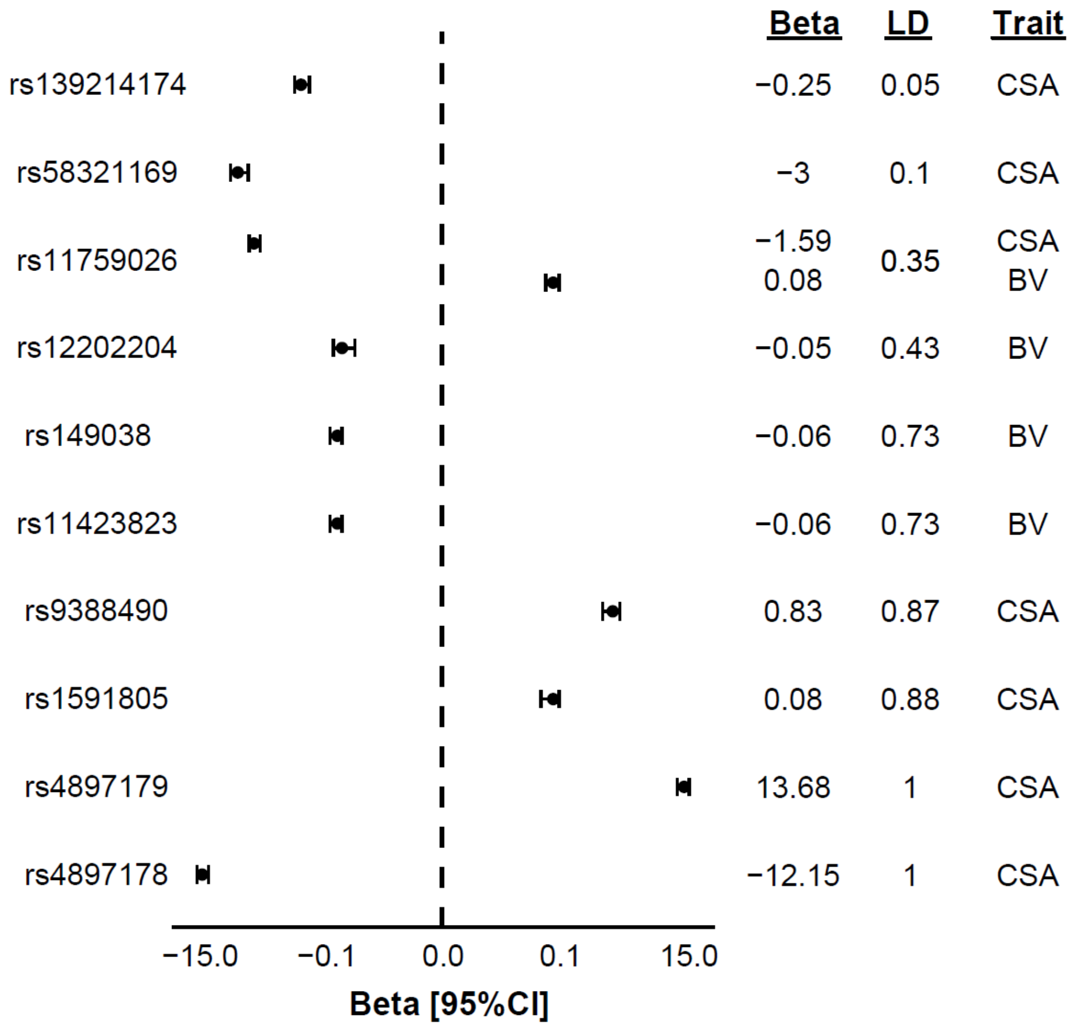

Supplementary Fig. 10. Associations between SNPs on CENPW and cortical surface area (CSA) and brain volume (BV) in existing studies.

Beta values and their 95% confidence intervals for each SNP were plotted in the forest plot, and LD were calculated for the corresponding SNP with the leading SNP from GWAS results in **Fig. 3b**.

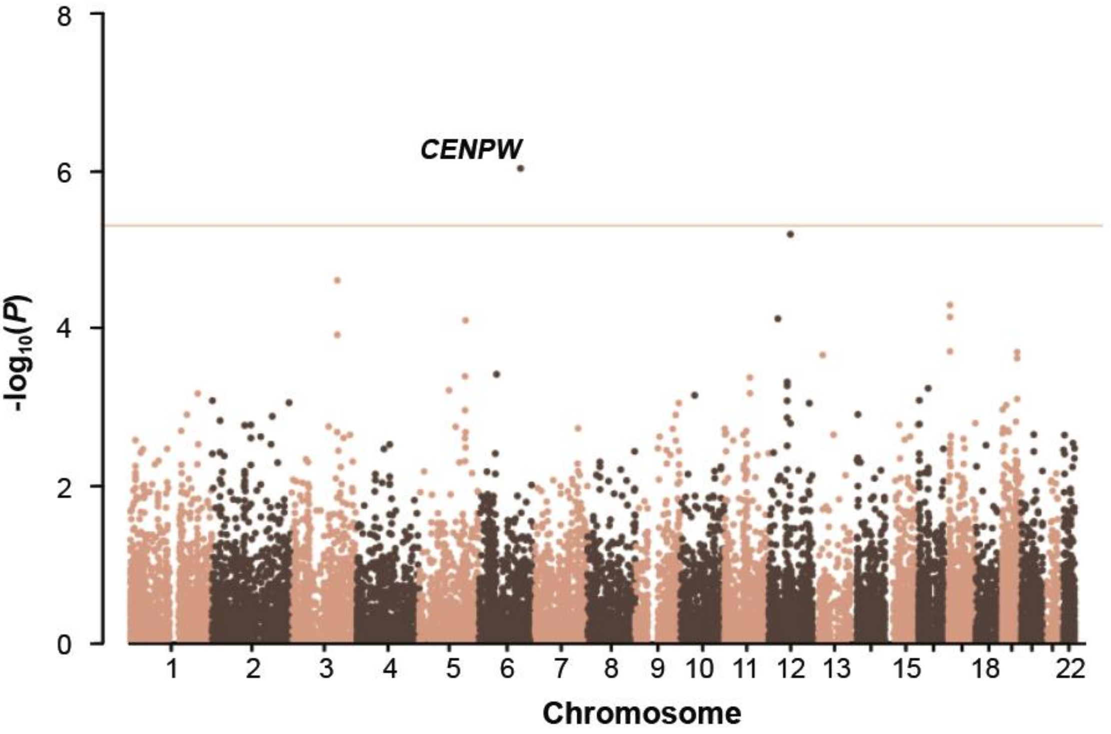

Supplementary Fig. 11. Gene-based GWAS results for delayed neurodevelopment.
Gene-based association tests (n = 7,662) identified one genome-wide significant genes (*CENPW*, $P = 9.39\times10^{-7}$) (significance level: $P = 5\times10^{-6}$) associated with delayed neurodevelopment in Group 3.

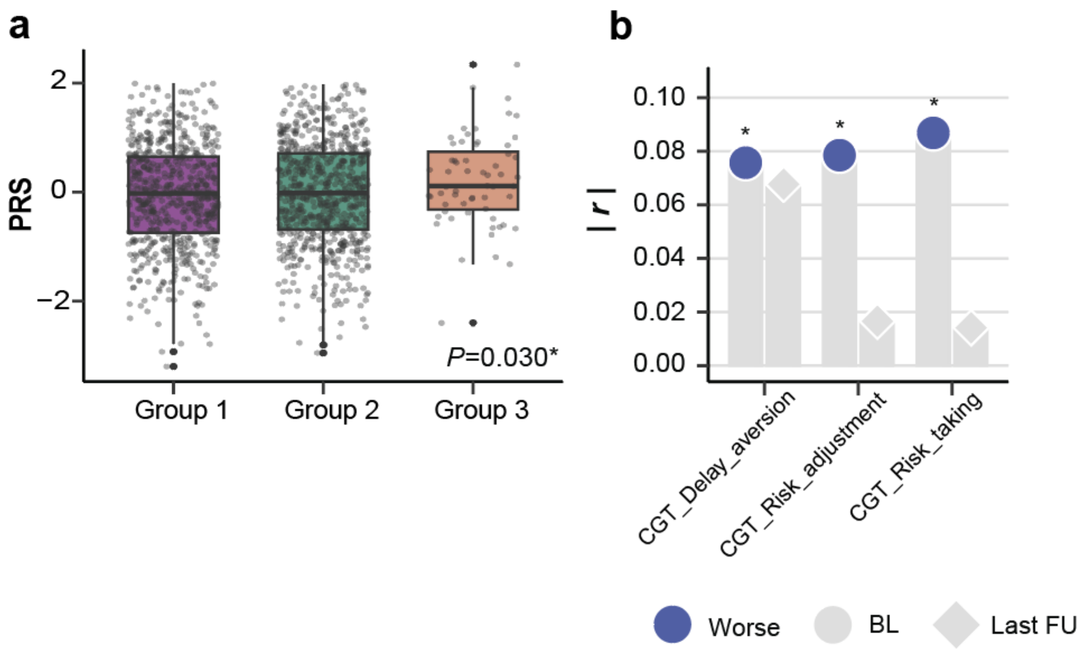

Supplementary Fig. 12. Validation of GWAS results for delayed neurodevelopment in IMAGEN.
Box plot in **(a)** showed that PRS of delayed neurodevelopment was higher in Group 3 (n = 60) compared to Group 1 and 2 (n = 1,338) (two-sided t-test: P = 0.030). (b) indicated that PRS of delayed neurodevelopment was negatively correlated with baseline neurocognitive performance, and became non-significant at the last follow-up. CGT delay aversion, BL (r = 0.08, *P_{adj} = 0.022), FU3 (r = -0.07, P_{adj} = 0.209); CGT risk adjustment, BL (r = -0.08, *P_{adj} = 0.022), FU3 (r = -0.02, P_{adj} = 0.711); CGT risk taking BL (r = 0.09, *P_{adj} = 0.018), FU3 (r = -0.01, P_{adj} = 0.711).

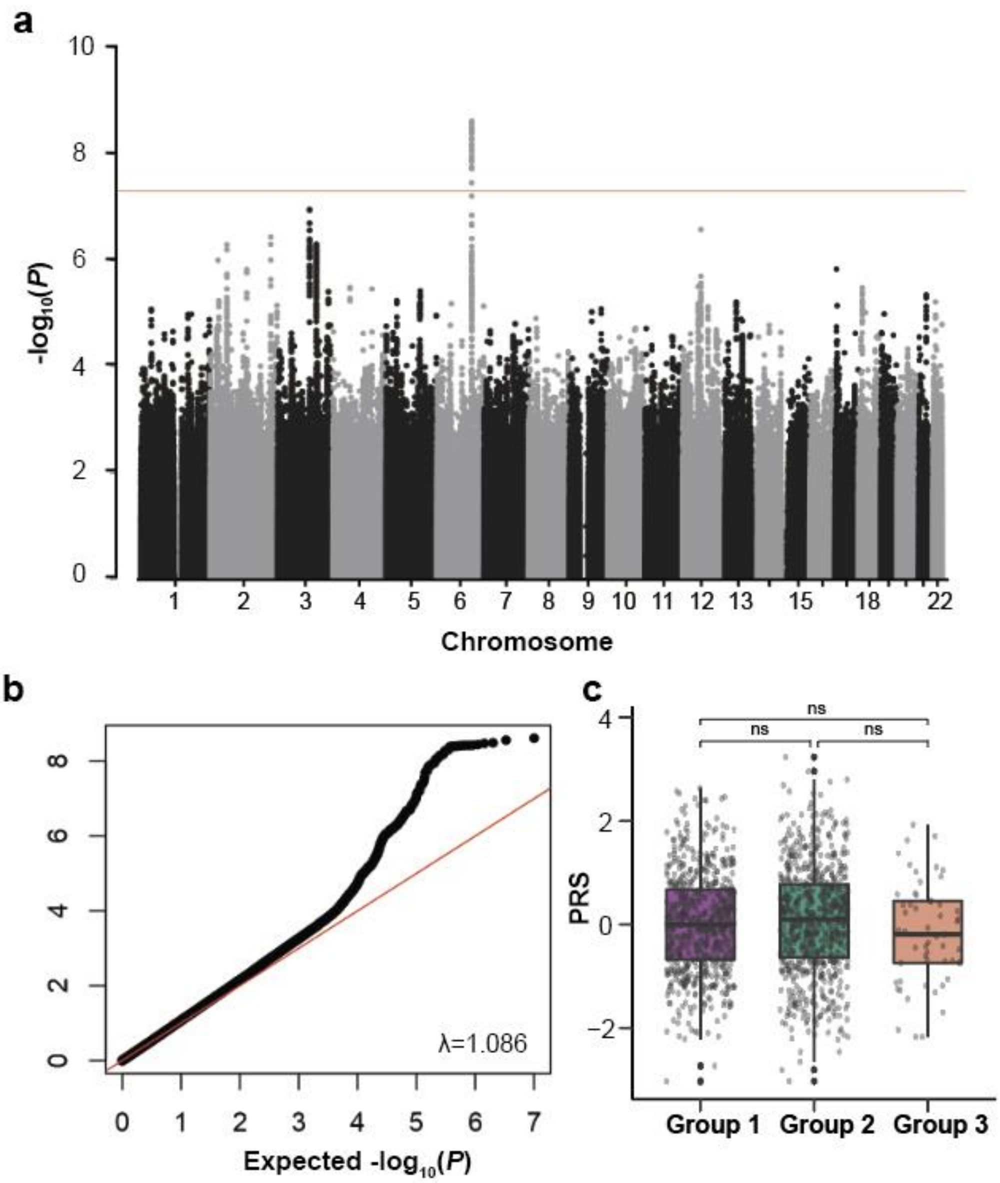

Supplementary Fig. 13. GWAS results and validation for Group 2 vs Group 1.

(a) GWAS Manhattan plot for Group2-reweighted GMV in the ABCD population (n = 7,662). Group2-reweighted GMV was calculated for each adolescent (**Methods**) and used as the proxy phenotype. **(b)** Q-Q plot of GWAS for Group 2 vs Group 1. **(c)** indicated that PRS of Group2-reweighted GWAS didn't differ among groups.

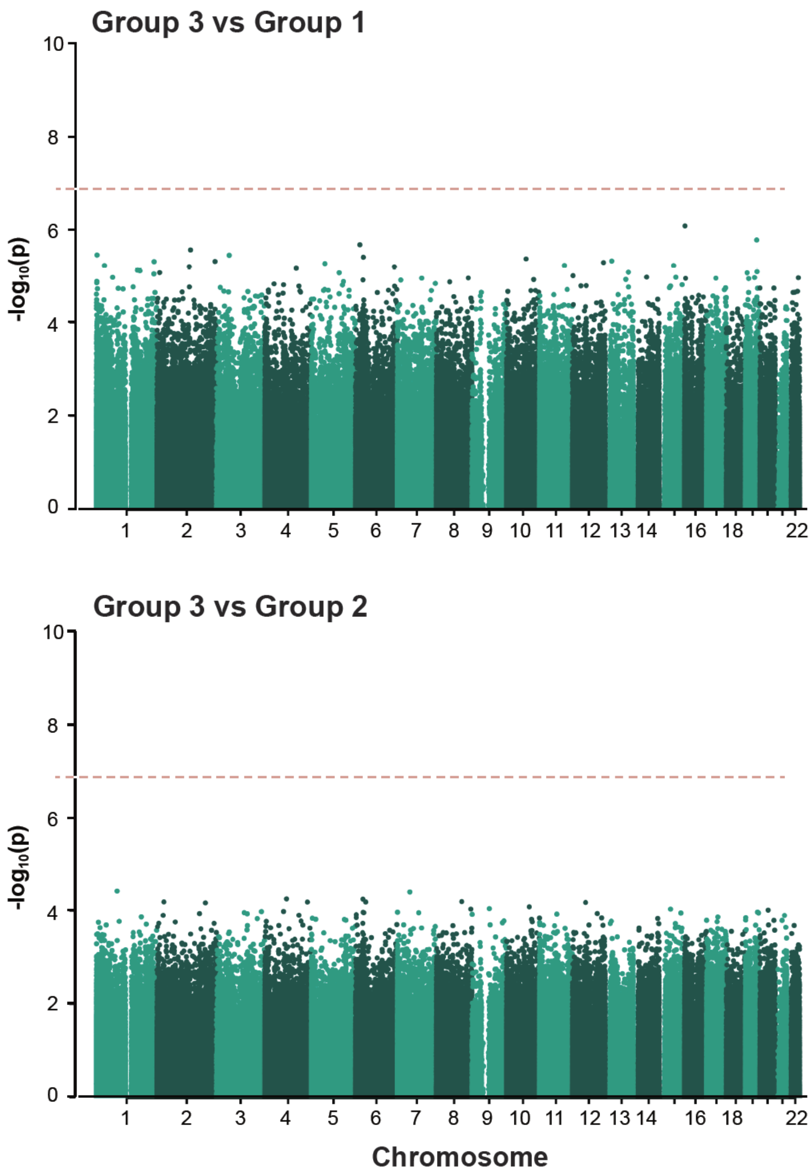

Supplementary Fig. 14. EWAS Manhattan plot in the IMAGEN population comparing Group 3 with Group 1 and Group 2.

Group 1 (n = 446) and Group 2 (n = 463) (relative to Group 3, n = 36) status was used as the phenotype, adjusting for potential confounders. No significant site was identified in the EWAS investigating Group 3 versus Group 1/2.

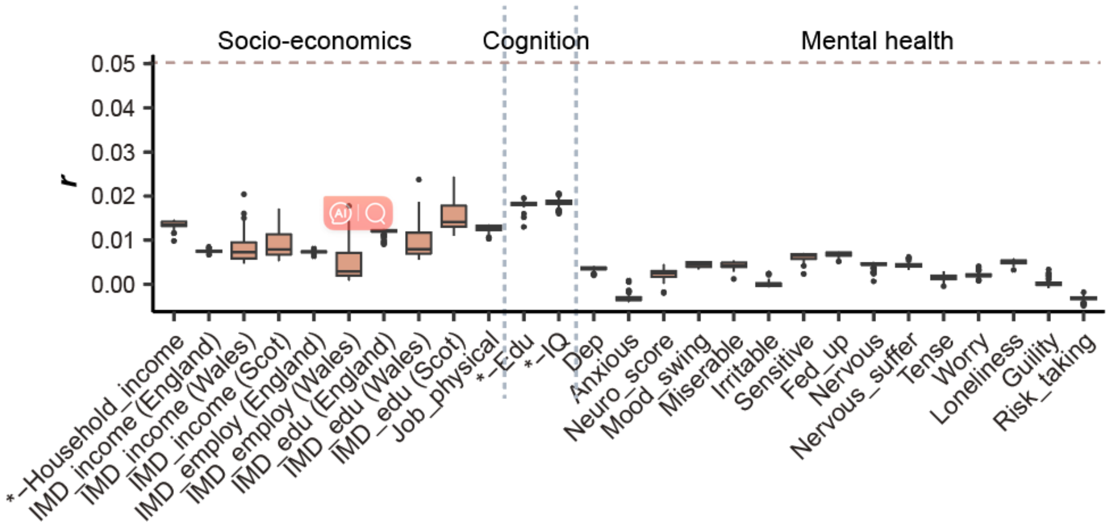

Supplementary Fig. 15. Correlations between all PRS and socio-economics, cognitive and mental health and in UKB.

Non-inferiority test against 0.05 correlation coefficient showed that genetic variants had limited effect on the long-term cognitive, mental health and socio-economic outcomes (*n* = 337,199).

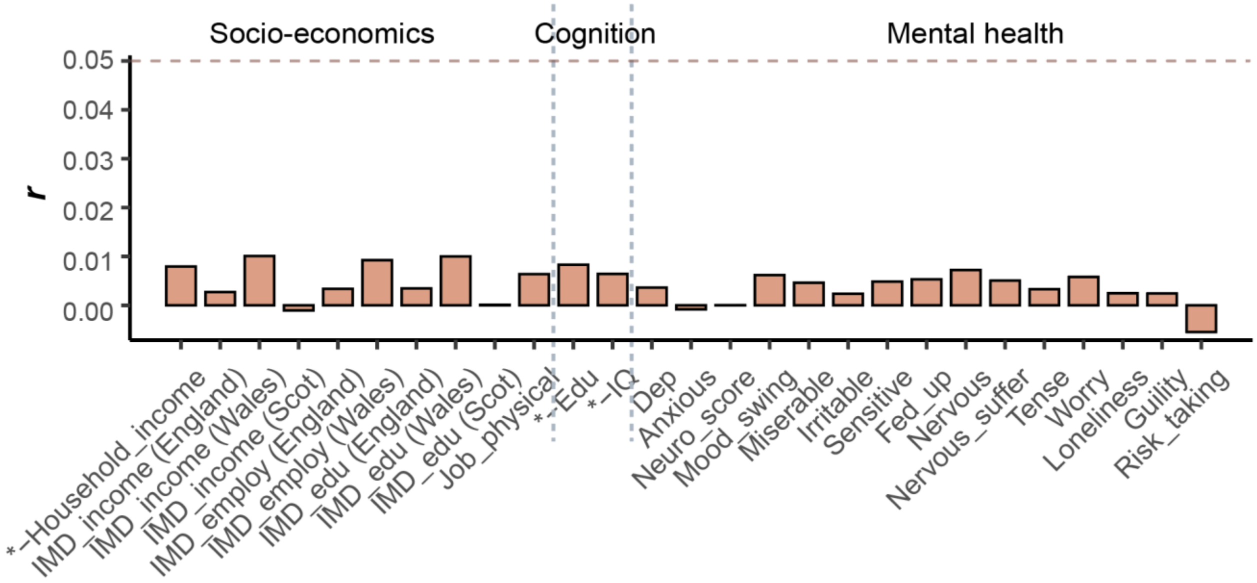

Supplementary Fig. 16. Correlations between CENPW score and socio-economics, cognitive and mental health and in UKB.

Non-inferiority test against 0.05 correlation coefficient showed that genetic variants had limited effect on the long-term cognitive, mental health and socio-economic outcomes (*n* = 337,199).

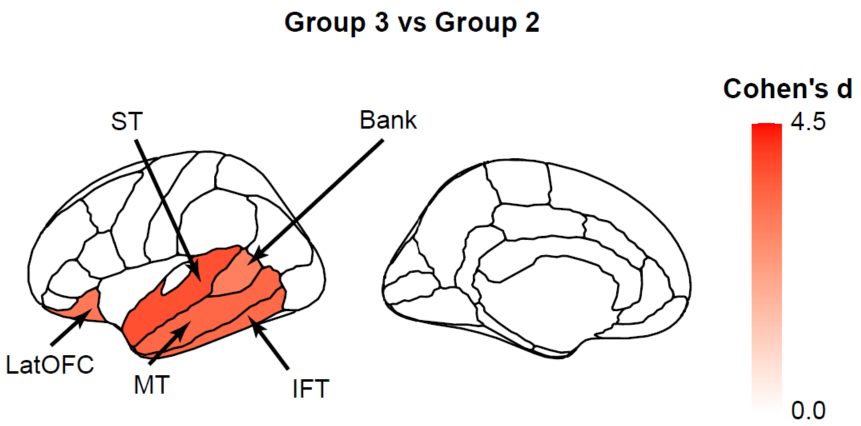

Supplementary Fig. 17. The top 5 discriminating ROIs with largest t values comparing the GMV trajectories between Group 3 and Group 2.

Sex, imaging site, handedness and ICV were adjusted. IFT ($d = 3.33$, $t = 16.65$, ***$P_{adj} < 0.001$), MT ($d = 3.37$, $t = 16.28$, ***$P_{adj} < 0.001$), LatOFC ($d = 3.05$, $t = 14.60$, ***$P_{adj} < 0.001$), ST ($d = 3.77$, $t = 14.55$, ***$P_{adj} < 0.001$), Bank ($d = 2.92$, $t = 14.50$, ***$P_{adj} < 0.001$). Bank, bankssts; IFT, inferior temporal; LatOFC, lateral orbitofrontal; MT, middle frontal; ST, superior temporal.

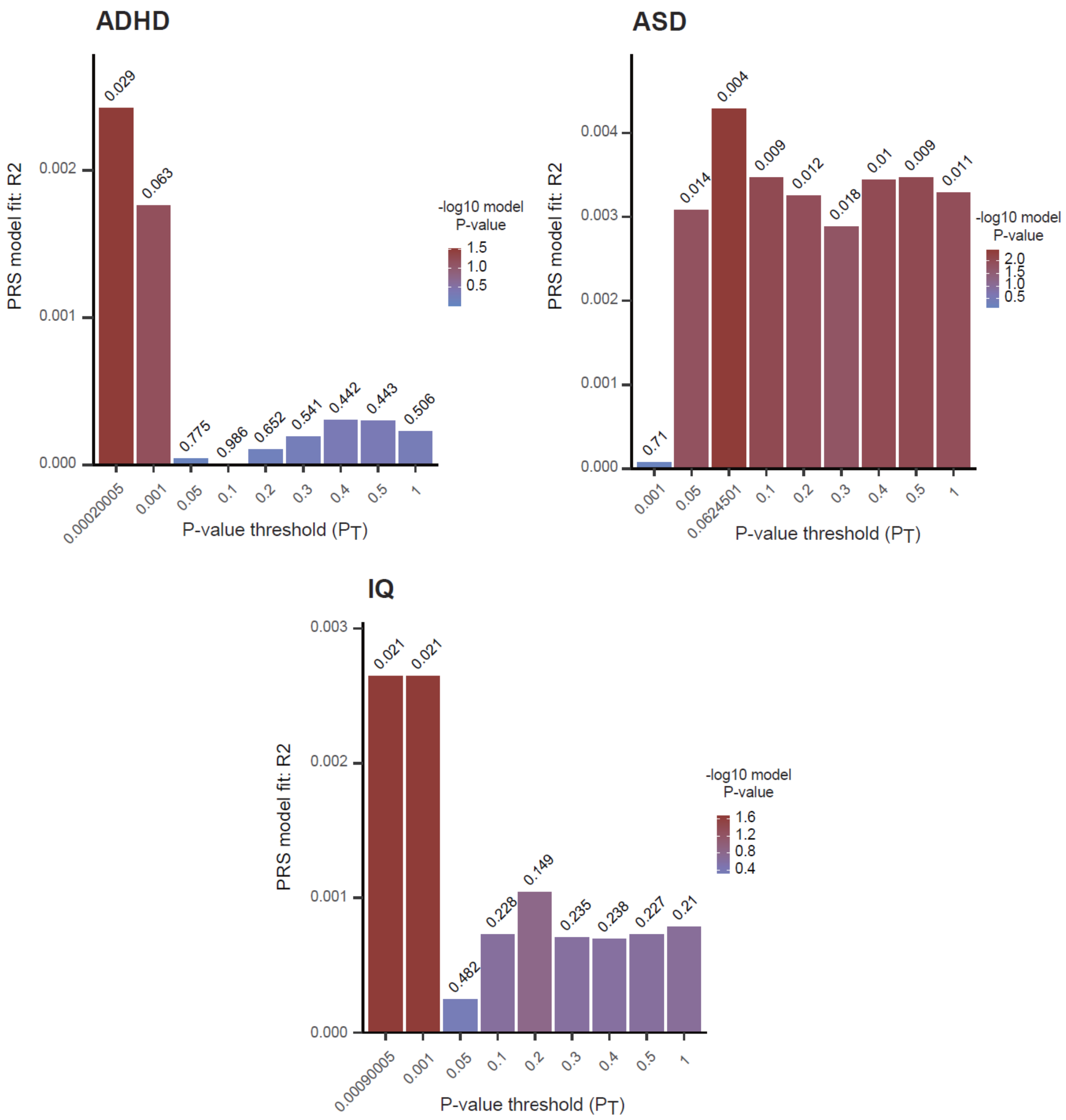

Supplementary Fig. 18. Polygenic risk scores (PGS) used to predict ADHD, ASD ad IQ in IMAGEN. Total variance in corresponding traits in IMAGEN explained by the PGS for multiple p value thresholds was shown. The red bar indicated the optimal p value threshold explaining the maximum amount of variance.

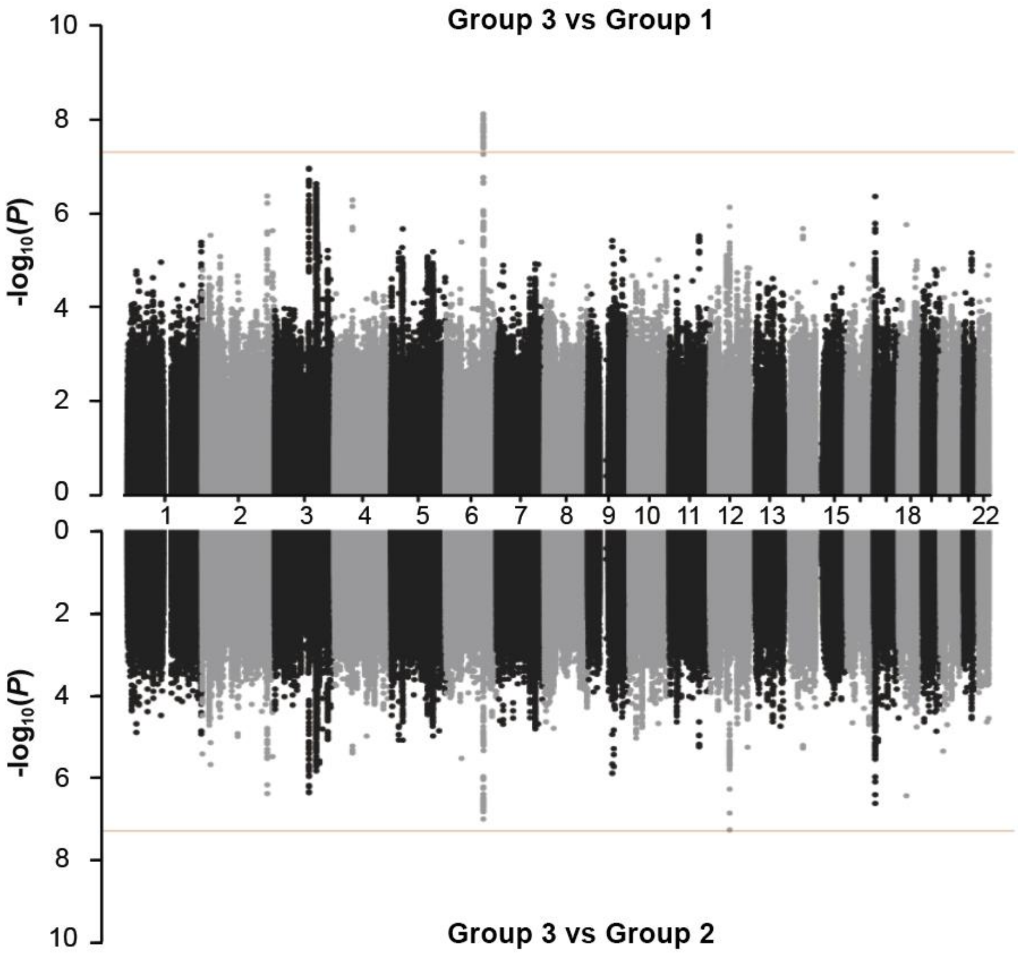

Supplementary Fig. 19. Miami plot of GWAS for Group 3 vs Group 1 and Group 2.
GWAS Manhattan plots for Group3-reweighted (vs Group 1 on top and Group 2 on bottom) GMV in the ABCD population (*n* = 7,662) were compared. Reweighted GMV was calculated for each adolescent (**Methods**) and used as the proxy phenotype.

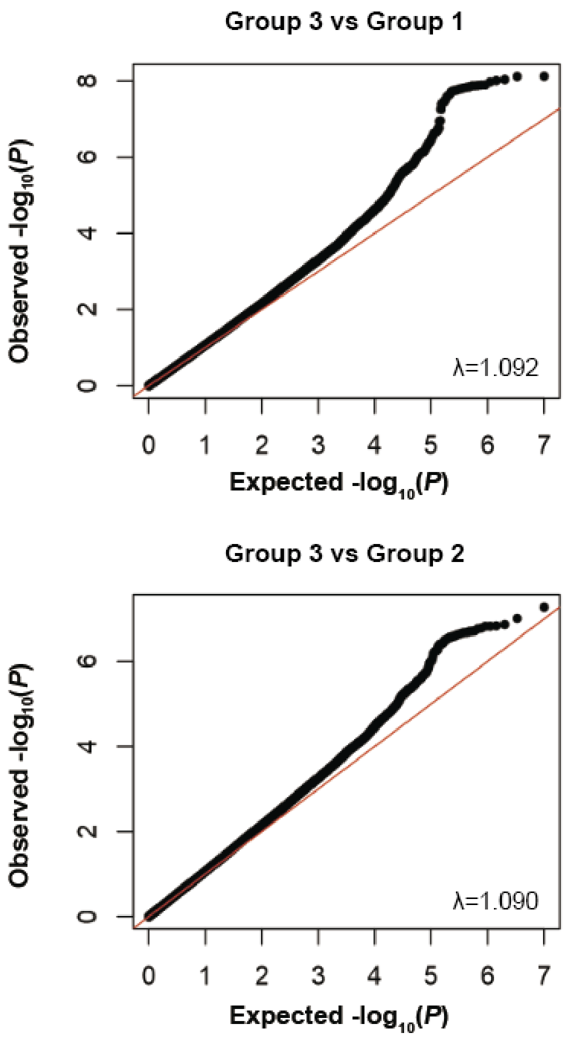

Supplementary Fig. 20. Q-Q plots of GWAS for Group 3 vs Group 1 and Group 2.

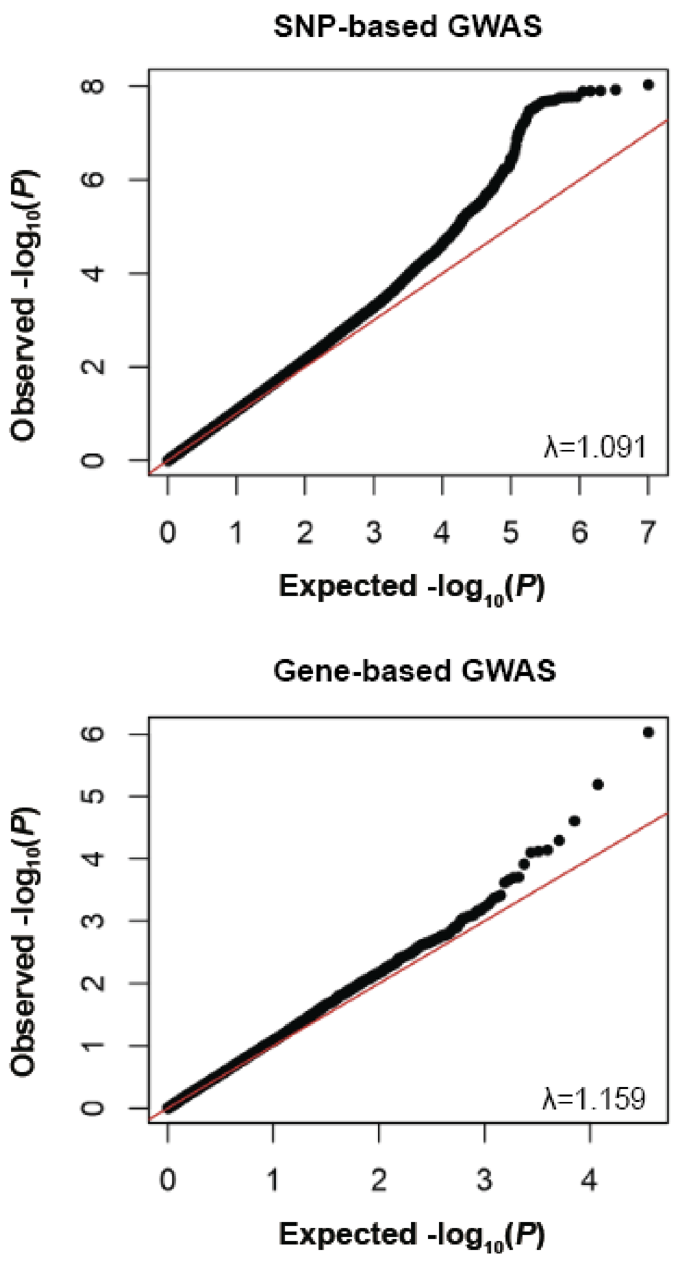

Supplementary Fig. 21. Q-Q plots of GWAS for Group 3 vs Group 1/2.

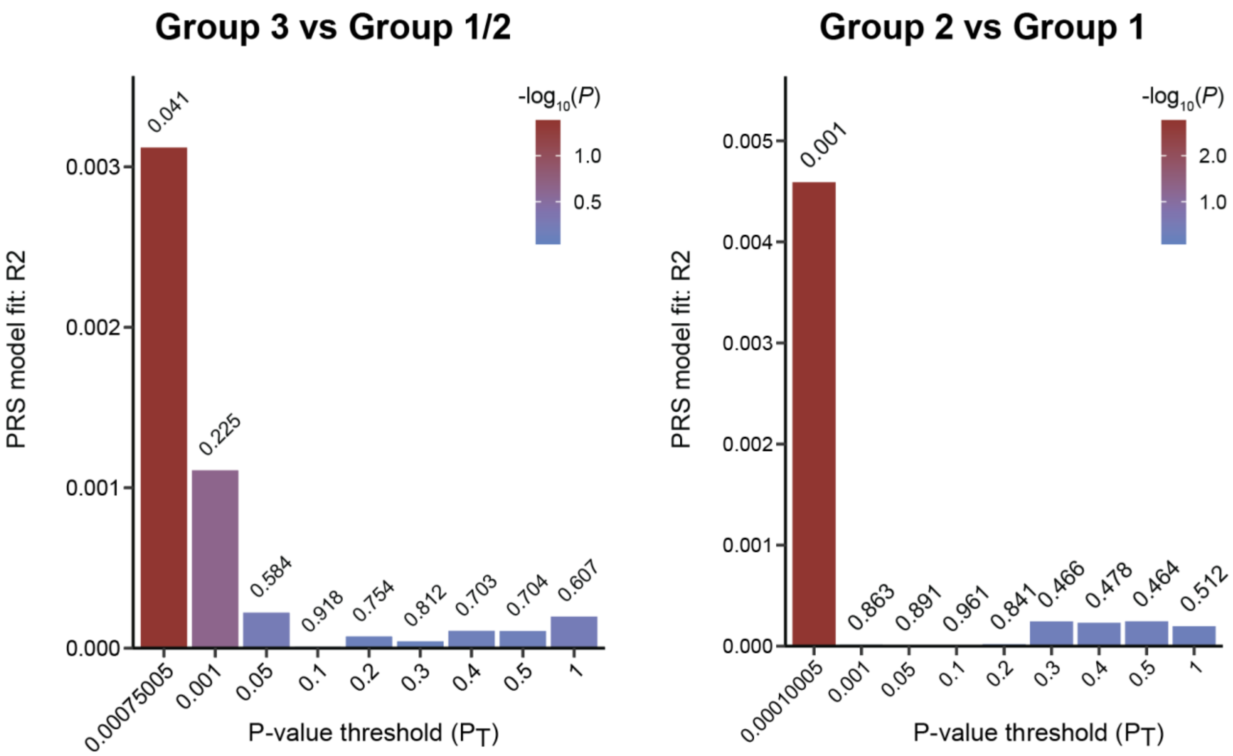

Supplementary Fig. 22. Polygenic risk scores (PGS) used to distinguish Group 3 from Group 1/2 and Group 2 from Group 1 in IMAGEN.

Total variance in the Group3-reweighted GMV and Group2-reweighted GMV in IMAGEN explained by the PGS for multiple p value thresholds was shown. The red bar indicated the optimal p value threshold explaining the maximum amount of variance.

Supplementary Table 1. Rotation matrix, factor loading and proportion of variance explained by each PCA component.

Brain region	Rotation matrix (Factor loadings)														
	**PC1**	**PC2**	**PC3**	**PC4**	**PC5**	**PC6**	**PC7**	**PC8**	**PC9**	**PC10**	**PC11**	**PC12**	**PC13**	**PC14**	**PC15**
**Cortical regions**															
bankssts	0.18	0.07	-0.04	0.03	-0.04	0.18	-0.09	0.12	-0.19	0.09	-0.14	0.09	0.00	-0.08	-0.07
	(0.75)	(0.12)	(-0.06)	(0.04)	(-0.05)	(0.21)	(-0.10)	(0.12)	(-0.18)	(0.08)	(-0.13)	(0.08)	(0.00)	(-0.06)	(-0.06)
caudalanteriorcingulate	0.17	0.03	-0.03	-0.12	0.04	0.06	0.27	-0.19	0.16	0.07	-0.21	-0.09	0.10	0.16	-0.02
	(0.71)	(0.05)	(-0.04)	(-0.17)	(0.04)	(0.07)	(0.29)	(-0.19)	(0.16)	(0.07)	(-0.19)	(-0.08)	(0.08)	(0.14)	(-0.01)
caudalmiddlefrontal	0.18	-0.16	-0.02	-0.19	0.04	0.01	0.14	0.04	0.09	0.08	0.12	-0.06	-0.06	-0.06	-0.01
	(0.78)	(-0.25)	(-0.03)	(-0.27)	(0.04)	(0.01)	(0.15)	(0.04)	(0.08)	(0.07)	(0.11)	(-0.06)	(-0.05)	(-0.05)	(-0.01)
cuneus	0.13	-0.18	0.13	0.27	0.25	-0.18	-0.18	-0.10	0.05	-0.11	-0.04	-0.08	0.00	-0.03	0.25
	(0.53)	(-0.29)	(0.19)	(0.37)	(0.28)	(-0.21)	(-0.19)	(-0.10)	(0.05)	(-0.11)	(-0.03)	(-0.07)	(0.00)	(-0.03)	(0.20)
entorhinal	0.12	0.13	-0.17	0.06	0.09	0.17	-0.04	-0.09	0.27	-0.22	0.20	0.27	-0.09	-0.01	-0.09
	(0.51)	(0.22)	(-0.24)	(0.09)	(0.10)	(0.19)	(-0.05)	(-0.09)	(0.27)	(-0.21)	(0.18)	(0.24)	(-0.07)	(-0.01)	(-0.07)
fusiform	0.19	0.20	-0.12	0.09	0.09	0.05	-0.14	0.06	-0.04	-0.03	-0.08	0.02	0.08	-0.04	0.02
	(0.80)	(0.32)	(-0.17)	(0.12)	(0.10)	(0.06)	(-0.15)	(0.06)	(-0.03)	(-0.03)	(-0.08)	(0.02)	(0.06)	(-0.03)	(0.01)
inferiorparietal	0.20	-0.09	-0.00	0.02	-0.06	0.12	-0.04	0.13	-0.14	-0.05	-0.09	-0.01	-0.02	-0.14	-0.01
	(0.84)	(-0.14)	(-0.00)	(0.02)	(-0.07)	(0.14)	(-0.04)	(0.13)	(-0.14)	(-0.05)	(-0.08)	(-0.01)	(-0.02)	(-0.12)	(-0.01)
inferiortemporal	0.18	0.23	-0.17	0.02	0.04	0.06	-0.11	0.15	-0.04	-0.08	0.02	0.01	0.01	-0.03	0.11
	(0.76)	(0.37)	(-0.25)	(0.03)	(0.04)	(0.06)	(-0.12)	(0.15)	(-0.04)	(-0.08)	(0.02)	(0.00)	(0.01)	(-0.02)	(0.09)
isthmuscingulate	0.16	0.00	0.15	0.30	-0.06	-0.04	-0.09	-0.07	-0.01	0.09	-0.20	-0.02	0.05	0.04	-0.10
	(0.67)	(0.00)	(0.22)	(0.41)	(-0.06)	(-0.05)	(-0.10)	(-0.07)	(-0.01)	(0.08)	(-0.18)	(-0.01)	(0.04)	(0.03)	(-0.08)
lateraloccipital	0.18	-0.08	0.01	0.12	0.13	-0.07	-0.14	0.12	-0.05	-0.05	-0.06	-0.01	0.04	-0.12	0.21
	(0.77)	(-0.13)	(0.02)	(0.16)	(0.14)	(-0.08)	(-0.15)	(0.12)	(-0.05)	(-0.05)	(-0.06)	(-0.01)	(0.03)	(-0.10)	(0.17)
lateralorbitofrontal	0.20	0.09	-0.11	-0.07	-0.08	-0.01	0.01	-0.11	0.03	-0.00	0.01	-0.08	-0.08	0.01	0.07
	(0.84)	(0.15)	(-0.16)	(-0.09)	(-0.09)	(-0.01)	(0.01)	(-0.11)	(0.03)	(-0.00)	(0.01)	(-0.07)	(-0.07)	(0.00)	(0.05)
lingual	0.16	-0.02	0.05	0.27	0.25	-0.18	-0.12	-0.08	0.01	0.02	-0.12	-0.04	0.10	-0.13	-0.07
	(0.68)	(-0.03)	(0.07)	(0.37)	(0.29)	(-0.20)	(-0.13)	(-0.08)	(0.01)	(0.02)	(-0.11)	(-0.03)	(0.09)	(-0.11)	(-0.05)
medialorbitofrontal	0.15	0.08	-0.04	-0.05	-0.22	-0.22	-0.01	-0.25	-0.06	-0.09	0.04	-0.05	0.02	0.03	0.03
	(0.64)	(0.13)	(-0.06)	(-0.07)	(-0.26)	(-0.25)	(-0.01)	(-0.25)	(-0.06)	(-0.09)	(0.04)	(-0.04)	(0.02)	(0.02)	(0.03)

middletemporal	0.19	0.20	-0.14	-0.03	0.03	0.07	-0.07	0.13	-0.10	-0.03	-0.08	0.00	0.02	-0.08	0.11
	(0.80)	(0.32)	(-0.21)	(-0.04)	(0.03)	(0.08)	(-0.07)	(0.13)	(-0.10)	(-0.03)	(-0.08)	(0.00)	(0.02)	(-0.06)	(0.09)
parahippocampal	0.12	0.23	-0.07	0.14	-0.05	0.11	-0.11	0.07	0.21	0.07	0.14	0.20	-0.19	0.05	-0.57
	(0.49)	(0.37)	(-0.10)	(0.19)	(-0.05)	(0.12)	(-0.11)	(0.07)	(0.20)	(0.06)	(0.13)	(0.18)	(-0.16)	(0.04)	(-0.46)
paracentral	0.15	-0.24	0.21	-0.07	-0.17	0.11	0.07	0.04	0.17	0.03	0.16	0.11	0.09	0.18	0.04
	(0.62)	(-0.38)	(0.31)	(-0.09)	(-0.20)	(0.13)	(0.08)	(0.04)	(0.16)	(0.02)	(0.15)	(0.09)	(0.08)	(0.15)	(0.03)
parsopercularis	0.18	-0.04	-0.04	-0.09	0.07	-0.09	0.01	-0.13	0.01	0.36	0.06	-0.08	-0.05	-0.04	0.02
	(0.76)	(-0.06)	(-0.07)	(-0.12)	(0.08)	(-0.10)	(0.01)	(-0.13)	(0.01)	(0.34)	(0.05)	(-0.07)	(-0.04)	(-0.04)	(0.01)
parsorbitalis	0.18	0.02	-0.16	-0.13	0.03	-0.15	-0.04	-0.08	-0.04	0.02	0.09	-0.04	-0.10	-0.10	0.08
	(0.75)	(0.04)	(-0.23)	(-0.18)	(0.04)	(-0.17)	(-0.04)	(-0.08)	(-0.04)	(0.02)	(0.08)	(-0.03)	(-0.09)	(-0.08)	(0.06)
parstriangularis	0.17	-0.01	-0.10	-0.13	0.08	-0.15	-0.01	-0.09	0.01	0.36	0.11	-0.02	-0.07	-0.03	0.09
	(0.74)	(-0.01)	(-0.14)	(-0.18)	(0.09)	(-0.17)	(-0.01)	(-0.09)	(0.01)	(0.34)	(0.10)	(-0.02)	(-0.06)	(-0.02)	(0.07)
pericalcarine	0.09	-0.14	0.18	0.24	0.15	-0.32	-0.10	-0.07	0.20	-0.14	0.02	-0.23	-0.33	0.11	-0.29
	(0.39)	(-0.23)	(0.27)	(0.33)	(0.17)	(-0.36)	(-0.11)	(-0.07)	(0.19)	(-0.13)	(0.02)	(-0.21)	(-0.28)	(0.09)	(-0.23)
postcentral	0.15	-0.25	0.20	-0.03	-0.19	0.04	0.02	0.07	-0.01	-0.20	0.09	-0.02	-0.00	-0.01	0.00
	(0.62)	(-0.41)	(0.29)	(-0.04)	(-0.22)	(0.05)	(0.02)	(0.07)	(-0.01)	(-0.19)	(0.08)	(-0.02)	(-0.00)	(-0.01)	(0.00)
posteriorcingulate	0.18	-0.03	0.16	0.03	-0.13	0.08	0.09	-0.11	0.15	0.16	-0.10	0.05	0.13	0.15	-0.11
	(0.77)	(-0.05)	(0.24)	(0.04)	(-0.15)	(0.09)	(0.10)	(-0.11)	(0.15)	(0.15)	(-0.10)	(0.05)	(0.11)	(0.13)	(-0.09)
precentral	0.19	-0.13	0.11	-0.11	-0.07	0.06	0.06	0.10	0.15	-0.06	0.12	0.02	-0.01	0.01	-0.03
	(0.82)	(-0.22)	(0.16)	(-0.15)	(-0.08)	(0.07)	(0.07)	(0.10)	(0.15)	(-0.06)	(0.11)	(0.02)	(-0.00)	(0.01)	(-0.02)
precuneus	0.20	-0.11	0.15	0.11	-0.06	0.13	-0.06	0.00	-0.01	0.01	-0.08	0.09	0.05	-0.00	0.01
	(0.83)	(-0.18)	(0.23)	(0.15)	(-0.07)	(0.15)	(-0.06)	(0.00)	(-0.01)	(0.01)	(-0.08)	(0.08)	(0.04)	(-0.00)	(0.01)
rostralanteriorcingulate	0.17	0.10	-0.09	-0.09	0.02	0.01	0.22	-0.19	0.09	-0.08	-0.25	-0.11	0.06	0.04	-0.07
	(0.74)	(0.17)	(-0.14)	(-0.12)	(0.02)	(0.01)	(0.23)	(-0.19)	(0.08)	(-0.08)	(-0.23)	(-0.10)	(0.05)	(0.03)	(-0.05)
rostralmiddlefrontal	0.20	-0.07	-0.15	-0.12	0.05	-0.13	0.02	-0.03	-0.03	0.11	0.09	-0.04	-0.10	-0.06	0.02
	(0.84)	(-0.11)	(-0.22)	(-0.16)	(0.06)	(-0.15)	(0.02)	(-0.03)	(-0.03)	(0.10)	(0.08)	(-0.04)	(-0.08)	(-0.05)	(0.02)
superiorfrontal	0.19	-0.17	-0.02	-0.20	-0.01	-0.07	0.10	-0.00	0.06	0.08	0.13	-0.03	-0.06	-0.03	-0.01
	(0.82)	(-0.27)	(-0.04)	(-0.28)	(-0.01)	(-0.08)	(0.10)	(-0.00)	(0.06)	(0.08)	(0.12)	(-0.02)	(-0.05)	(-0.02)	(-0.00)
superiorparietal	0.17	-0.22	0.10	-0.02	-0.08	0.13	-0.07	0.14	-0.08	-0.18	-0.00	0.05	-0.04	-0.09	0.06
	(0.73)	(-0.36)	(0.15)	(-0.03)	(-0.09)	(0.15)	(-0.08)	(0.14)	(-0.08)	(-0.17)	(-0.00)	(0.04)	(-0.03)	(-0.08)	(0.05)
superiortemporal	0.21	0.12	-0.06	-0.03	-0.01	0.05	-0.06	0.05	-0.04	0.10	-0.04	0.05	0.00	-0.02	0.02

	(0.89)	(0.19)	(-0.08)	(-0.04)	(-0.01)	(0.05)	(-0.06)	(0.05)	(-0.04)	(0.09)	(-0.04)	(0.04)	(0.00)	(-0.01)	(0.01)
supramarginal	0.19	-0.11	0.06	-0.03	-0.18	0.13	-0.07	0.13	-0.06	-0.16	-0.06	0.07	-0.01	-0.07	-0.04
	(0.79)	(-0.18)	(0.09)	(-0.05)	(-0.21)	(0.15)	(-0.08)	(0.13)	(-0.06)	(-0.15)	(-0.05)	(0.06)	(-0.01)	(-0.06)	(-0.04)
frontalpole	0.07	-0.06	-0.06	-0.12	-0.15	-0.48	-0.07	-0.16	-0.43	-0.12	0.05	0.46	0.27	0.11	-0.26
	(0.29)	(-0.10)	(-0.09)	(-0.16)	(-0.17)	(-0.54)	(-0.07)	(-0.16)	(-0.42)	(-0.11)	(0.05)	(0.41)	(0.23)	(0.09)	(-0.21)
temporalpole	0.12	0.15	-0.13	0.04	-0.01	0.08	-0.16	-0.28	0.09	-0.35	0.26	0.04	-0.02	0.42	0.30
	(0.51)	(0.24)	(-0.19)	(0.06)	(-0.01)	(0.09)	(-0.17)	(-0.28)	(0.09)	(-0.33)	(0.24)	(0.04)	(-0.02)	(0.35)	(0.25)
transversetemporal	0.16	0.04	0.09	0.14	-0.02	0.08	-0.16	0.07	0.04	0.38	0.02	0.12	0.20	0.18	0.09
	(0.66)	(0.06)	(0.13)	(0.19)	(-0.02)	(0.09)	(-0.18)	(0.07)	(0.04)	(0.36)	(0.02)	(0.11)	(0.17)	(0.15)	(0.07)
insula	0.14	0.02	-0.10	-0.08	0.06	0.10	0.19	-0.11	-0.05	-0.27	-0.41	-0.10	-0.06	-0.09	-0.09
	(0.60)	(0.03)	(-0.15)	(-0.11)	(0.06)	(0.12)	(0.20)	(-0.11)	(-0.04)	(-0.26)	(-0.37)	(-0.09)	(-0.05)	(-0.07)	(-0.08)
**Subcortical regions**															
cerebellum_white_matter	-0.03	0.22	0.29	-0.17	-0.27	-0.12	-0.12	-0.14	0.09	0.01	-0.13	0.23	-0.56	-0.32	0.19
	(-0.11)	(0.36)	(0.43)	(-0.24)	(-0.31)	(-0.14)	(-0.12)	(-0.14)	(0.09)	(0.01)	(-0.12)	(0.21)	(-0.47)	(-0.26)	(0.15)
cerebellum_cortex	0.11	0.04	-0.02	-0.26	0.12	-0.22	-0.06	0.44	-0.04	-0.19	0.19	-0.29	0.04	0.06	-0.16
	(0.47)	(0.07)	(-0.04)	(-0.35)	(0.14)	(-0.25)	(-0.07)	(0.44)	(-0.04)	(-0.18)	(0.17)	(-0.26)	(0.03)	(0.05)	(-0.13)
thalamus_proper	0.01	0.07	0.39	-0.28	0.30	-0.02	-0.09	0.01	-0.16	0.01	-0.23	0.21	-0.12	0.29	0.06
	(0.04)	(0.11)	(0.57)	(-0.39)	(0.35)	(-0.02)	(-0.10)	(0.01)	(-0.15)	(0.00)	(-0.21)	(0.19)	(-0.10)	(0.24)	(0.04)
caudate	0.11	0.05	0.13	0.13	0.12	0.12	0.43	-0.12	-0.38	-0.10	0.05	-0.07	-0.15	0.17	-0.06
	(0.47)	(0.09)	(0.19)	(0.17)	(0.14)	(0.14)	(0.45)	(-0.12)	(-0.37)	(-0.10)	(0.05)	(-0.06)	(-0.13)	(0.14)	(-0.05)
putamen	0.08	0.11	0.16	0.29	0.10	0.15	0.27	-0.10	-0.37	0.09	0.45	-0.00	-0.14	-0.23	0.01
	(0.33)	(0.18)	(0.24)	(0.39)	(0.11)	(0.17)	(0.29)	(-0.10)	(-0.36)	(0.08)	(0.41)	(-0.00)	(-0.12)	(-0.19)	(0.01)
pallidum	-0.00	0.16	0.31	-0.21	0.03	0.13	-0.20	-0.37	0.08	-0.13	0.17	-0.21	0.45	-0.44	-0.17
	(-0.00)	(0.27)	(0.46)	(-0.29)	(0.03)	(0.15)	(-0.21)	(-0.37)	(0.07)	(-0.12)	(0.15)	(-0.19)	(0.38)	(-0.36)	(-0.13)
hippocampus	0.05	0.37	0.22	-0.01	-0.16	-0.18	0.08	0.23	-0.02	0.03	-0.04	-0.22	0.00	0.07	-0.14
	(0.22)	(0.61)	(0.32)	(-0.02)	(-0.18)	(-0.20)	(0.08)	(0.23)	(-0.02)	(0.02)	(-0.04)	(-0.19)	(0.00)	(0.05)	(-0.11)
amygdala	0.04	0.35	0.24	0.08	-0.27	-0.11	0.02	0.12	-0.00	0.02	0.10	-0.23	0.11	0.20	0.23
	(0.19)	(0.57)	(0.36)	(0.11)	(-0.31)	(-0.13)	(0.02)	(0.12)	(-0.00)	(0.02)	(0.09)	(-0.20)	(0.09)	(0.17)	(0.19)
accumbens_area	0.05	0.08	-0.01	0.23	-0.07	-0.34	0.49	0.20	0.30	-0.11	-0.03	0.31	0.20	-0.25	0.18
	(0.22)	(0.13)	(-0.02)	(0.32)	(-0.08)	(-0.38)	(0.52)	(0.20)	(0.30)	(-0.10)	(-0.03)	(0.27)	(0.17)	(-0.21)	(0.15)
brain_stem	0.04	0.18	0.25	-0.22	0.52	0.03	0.11	0.11	0.14	-0.05	0.09	0.28	0.09	-0.01	0.04

	(0.15)	(0.30)	(0.37)	(-0.30)	(0.60)	(0.03)	(0.12)	(0.11)	(0.13)	(-0.04)	(0.08)	(0.24)	(0.07)	(-0.01)	(0.03)
Standard deviation	4.24	1.63	1.47	1.37	1.15	1.14	1.07	1.00	0.97	0.94	0.92	0.89	0.85	0.83	0.81
Proportion of variance	0.41	0.06	0.05	0.04	0.03	0.03	0.03	0.02	0.02	0.02	0.02	0.02	0.02	0.02	0.01
Cumulative proportion	0.41	0.47	0.52	0.56	0.59	0.62	0.65	0.67	0.69	0.71	0.73	0.75	0.76	0.78	0.79

Supplementary Table 2. Full demographics and baseline characteristics for Group 1, Group 2 and Group 3 in IMAGEN.

Variable	Adolescents selected	Group 1	Group 2	Group 3	*P**	*P**
	(*N*=1543)	(*N*=711)	(*N*=765)	(*N*=67)	(Group 2 v.s. Group 1)	(Group 3 v.s. Group 1)
Baseline age (y; mean ± s.d.)	14.39 ± 0.39	14.33 ± 0.39	14.44 ± 0.38	14.32 ± 0.39	3.70E-04	0.159
Birthweight (g)	3434.84 ± 578.04	3498.28 ± 582.77	3381.81 ± 565.65	3388.98 ± 612.15	7.21E-05	0.069
WISCIV full score	176.89 ± 22.50	178.16 ± 22.67	176.40 ± 21.82	168.94 ± 26.39	0.011	1.48E-05
WISCIV Block Design	51.17 ± 9.41	51.78 ± 9.08	50.84 ± 9.39	48.48 ± 12.17	0.005	6.39E-05
WISCIV Digit Span backward	8.76 ± 2.09	8.79 ± 2.14	8.73 ± 2.01	8.65 ± 2.41	0.131	0.241
WISCIV Digit Span forward	9.68 ± 2.10	9.71 ± 2.18	9.66 ± 2.05	9.48 ± 1.94	0.513	0.264
WISCIV Matrix Reasoning	26.42 ± 4.24	26.48 ± 4.24	26.40 ± 4.18	25.92 ± 5.00	0.129	0.025
WISCIV Similarities	30.63 ± 5.53	31.05 ± 5.57	30.39 ± 5.42	28.86 ± 5.81	0.105	0.004
WISCIV Vocabulary	50.16 ± 8.57	50.32 ± 8.62	50.23 ± 8.44	47.56 ± 9.16	0.081	3.69E-04
Male (n, %)	747, 48.4%	335, 47.1%	433, 56.6%	28, 41.8%	6.49E-05	0.497
Maternal ethnicity					2.03E-04	0.011
White	1423, 92.8%	673, 95.2%	690, 90.8%	60, 89.6%		
Black	29, 1.9%	9, 1.3%	19, 2.5%	1, 1.5%		
Other	82, 5.3%	25, 3.5%	51, 6.7%	6, 9.0%		
Paternal ethnicity					3.69E-04	0.002
White	1450, 94.6%	682, 96.5%	707, 93.1%	61, 91.0%		
Black	23, 1.5%	9, 1.3%	11, 1.4%	3, 4.5%		
Other	60, 3.9%	16, 2.3%	41, 5.4%	3, 4.5%		
Maternal education					0.020	0.015
Professional qualification	201, 13.1%	120, 16.9%	77, 10.1%	4, 6.1%		
Bachelor degree	381, 24.8%	181, 25.5%	186, 24.4%	14, 21.2%		
Advanced diploma	266, 17.3%	123, 17.3%	133, 17.5%	10, 15.2%		
A levels or a BTEC national diploma	219, 14.2%	120, 16.9%	89, 11.7%	10, 15.2%		
NVQ or GNVQ	286, 18.6%	98, 13.8%	171, 22.4%	17, 25.8%		
O levels, GCSEs/CSEs or less	163, 10.6%	57, 8.0%	95, 12.5%	11, 16.7%		

None of the above	23, 1.5%	12, 1.7%	11, 1.4%	0, 0.0%		
Paternal education					0.003	0.010
Professional qualification	282, 18.3%	171, 24.1%	106, 13.9%	5, 7.6%		
Bachelor degree	360, 23.4%	166, 23.3%	180, 23.6%	14, 21.2%		
Advanced diploma	203, 13.2%	96, 13.5%	98, 12.9%	9, 13.6%		
A levels or a BTEC national diploma	184, 12.0%	82, 11.5%	94, 12.3%	8, 12.1%		
NVQ or GNVQ	231, 15.0%	88, 12.4%	128, 16.8%	15, 22.7%		
O levels, GCSEs/CSEs or less	256, 16.6%	99, 13.9%	144, 18.9%	13, 19.7%		
None of the above	23, 1.5%	9, 1.3%	12, 1.6%	2, 3.0%		
Handedness					0.829	0.807
Left Handedness	157, 10.2%	75, 10.5%	75, 9.8%	7, 10.4%		
Right Handedness	1386, 89.8%	636, 89.5%	690, 90.2%	60, 89.6%		
Mother smoking during pregnancy†					0.146	0.127
No	577, 76.1%	272, 77.7%	283, 75.1%	22, 71.0%		
Yes	181, 23.9%	78, 22.3%	94, 24.9%	9, 29.0%		
Father smoking during pregnancy					0.347	0.354
No	980, 69.2%	456, 70.8%	484, 68.0%	40, 66.7%		
Yes	436, 30.8%	188, 29.2%	228, 32.0%	20, 33.3%		
Mother drink during pregnancy					0.788	0.777
No	1072, 75.7%	484, 75.2%	539, 75.8%	49, 80.3%		
Yes	344, 24.3%	160, 24.8%	172, 24.2%	12, 19.7%		
Mother medical problems during pregnancy					0.358	0.225
No	1260, 88.9%	581, 89.8%	628, 88.3%	51, 86.4%		
Yes	157, 11.1%	66, 10.2%	83, 11.7%	8, 13.6%		
Family psychiatric history					0.693	0.534
No	731, 48.5%	332, 47.6%	365, 48.9%	34, 53.1%		
Yes	776, 51.5%	365, 52.4%	381, 51.1%	30, 46.9%		
Family Stresses						
Socioeconomic/housing score	0.56 ± 0.86	0.42 ± 0.64	0.68 ± 0.99	0.59 ± 0.81	6.14E-06	0.093

Work/pressure score	1.00 ± 0.92	0.96 ± 0.91	1.01 ± 0.93	1.13 ± 0.88	0.958	0.333
Health score	0.50 ± 0.75	0.42 ± 0.65	0.54 ± 0.79	0.72 ± 0.98	0.014	0.004
Relationship/addiction score	0.32 ± 0.53	0.24 ± 0.36	0.39 ± 0.63	0.34 ± 0.56	1.07E-05	0.143
Childs experience of family life						
Parental support	10.84 ± 1.33	10.87 ± 1.26	10.82 ± 1.38	10.83 ± 1.40	0.045	0.254
Discipline	3.28 ± 1.41	3.29 ± 1.39	3.25 ± 1.42	3.47 ± 1.38	0.843	0.082
Rules	4.67 ± 1.09	4.69 ± 1.06	4.66 ± 1.13	4.61 ± 1.00	0.839	0.648
Special allowances	5.02 ± 1.53	5.02 ± 1.52	5.01 ± 1.56	5.10 ± 1.37	0.099	0.920

* Multivariate ordinal model for Parental education and generalized linear model for all other variables, adjusted for Imaging Center, were used to compare between Group 2, Group 3 and Group 1. One sample two-sided t tests were used. BH-FDR method was used for multiple correction. Parental ethnicity was only compared between white and non-white.

† More than 50% of the variable are missing.

Supplementary Table 3. t-map and d-map of the top 20 distinct brain regions in the group clustering in IMAGEN.

Group 2 vs Group 1	*Cohen's d*	*t*	P_{adj}	Group 3 vs Group 1	*Cohen's d*	*t*	P_{adj}	Group 3 vs Group 2	*Cohen's d*	*t*	P_{adj}
superiorfrontal	1.28	24.50	3.740E-109	inferiortemporal	4.43	20.13	2.891E-29	inferiortemporal	3.33	16.65	2.080E-24
rostralmiddlefrontal	1.14	21.95	2.945E-91	middletemporal	4.38	20.07	2.891E-29	middletemporal	3.37	16.28	4.160E-24
caudalmiddlefrontal	1.09	20.77	7.301E-83	lateralorbitofrontal	4.26	18.31	4.082E-27	lateralorbitofrontal	3.05	14.60	9.828E-22
precentral	1.05	20.14	9.267E-79	precentral	3.63	18.11	4.082E-27	superiortemporal	3.77	14.55	1.257E-21
inferiorparietal	1.00	19.07	1.798E-71	superiorfrontal	3.61	17.92	5.861E-27	bankssts	2.92	14.50	9.828E-22
superiorparietal	0.98	18.74	2.993E-69	rostralmiddlefrontal	4.01	17.72	1.532E-26	fusiform	3.52	13.87	1.132E-20
postcentral	0.96	18.36	1.392E-66	bankssts	3.52	17.52	1.532E-26	precentral	2.73	13.35	4.092E-20
superiortemporal	0.91	17.54	5.635E-62	superiortemporal	4.67	17.41	5.480E-26	rostralmiddlefrontal	2.99	13.24	6.913E-20
supramarginal	0.91	17.37	9.660E-61	fusiform	4.31	16.20	2.475E-24	superiorfrontal	2.80	12.49	1.072E-18
lateralorbitofrontal	0.89	17.24	3.795E-60	caudalmiddlefrontal	3.29	15.73	5.270E-24	rostralanteriorcingulate	2.67	12.18	2.966E-18
lateraloccipital	0.90	17.08	5.475E-59	supramarginal	2.94	15.00	4.754E-23	parsorbitalis	2.49	11.74	1.337E-17
middletemporal	0.88	16.97	1.321E-58	parsorbitalis	3.29	14.75	1.722E-22	temporalpole	1.90	11.65	1.045E-17
precuneus	0.85	16.38	5.637E-55	rostralanteriorcingulate	3.24	14.51	3.450E-22	caudalmiddlefrontal	2.51	11.24	9.941E-17
paracentral	0.81	15.41	3.495E-49	parstriangularis	2.85	14.45	3.165E-22	parstriangularis	2.35	11.15	1.185E-16
inferiortemporal	0.79	15.15	7.225E-48	inferiorparietal	3.44	14.39	6.057E-22	supramarginal	2.39	11.01	2.029E-16
parsopercularis	0.76	14.63	7.531E-45	temporalpole	2.23	13.68	1.995E-21	inferiorparietal	2.85	11.01	2.305E-16
parstriangularis	0.74	14.15	2.723E-42	transversetemporal	2.74	13.58	6.455E-21	transversetemporal	2.11	10.91	2.299E-16
parsorbitalis	0.72	13.90	4.951E-41	superiorparietal	2.62	13.11	3.555E-20	posteriorcingulate	2.73	10.68	8.125E-16
fusiform	0.72	13.90	4.951E-41	posteriorcingulate	3.12	12.80	1.721E-19	caudalanteriorcingulate	2.32	9.94	1.306E-14
bankssts	0.66	12.68	1.012E-34	lateraloccipital	2.77	12.59	3.021E-19	entorhinal	1.89	9.92	1.142E-14

Two-sample two-sided t-test was used.

Supplementary Table 4. Full comparison of Group 2 and Group 3 vs Group 1 in terms of the personal traits, environmental burden, neuro-cognition, behavioral risk factors and mental symptoms at age 14, longitudinal trajectory and at age 23.

Questionnaire	Item	Time	Group 2 vs Group 1		Group 3 vs Group 1		Group 3 vs Group 2	
			Cohen's d	P_{adj}	***Cohen's d***	P_{adj}	***Cohen's d***	P_{adj}
NEO	Neuroticism	14y	0.02	0.346	-0.19	0.485	-0.23	0.230
		trajectory	0.01	0.848	0.20	0.381	0.19	0.287
		23y	0.10	0.078	0.05	0.746	-0.06	0.732
	Extraversion	14y	0.00	0.579	0.16	0.478	0.16	0.482
		trajectory	-0.08	0.149	-0.25	0.233	-0.20	0.287
		23y	-0.11	0.068	-0.18	0.315	-0.08	0.708
	Openness	14y	0.00	0.394	-0.19	0.091	-0.18	0.196
		trajectory	-0.08	0.149	-0.05	0.814	0.04	0.830
		23y	-0.04	0.348	-0.24	0.315	-0.19	0.632
	Agreeableness	14y	-0.05	0.346	-0.10	0.688	-0.04	0.966
		trajectory	-0.13	0.087	0.12	0.814	0.25	0.287
		23y	-0.19	0.002	-0.05	0.746	0.13	0.632
	Conscientiousness	14y	0.00	0.515	0.00	0.688	0.00	0.966
		trajectory	-0.09	0.149	0.05	0.838	0.14	0.448
		23y	-0.10	0.078	0.04	0.945	0.15	0.632
TCI	Exploratory excitability	14y	0.04	0.943	0.08	0.883	0.03	0.923
		trajectory	-0.09	0.440	-0.16	0.349	-0.07	0.574
		23y	-0.11	0.095	-0.08	0.707	0.03	0.979
	Impulsiveness	14y	-0.02	0.943	0.23	0.089	0.26	0.286
		trajectory	0.04	0.889	-0.21	0.349	-0.24	0.226
		23y	-0.03	0.698	-0.13	0.707	-0.10	0.979
	Extravagance	14y	0.00	0.943	0.11	0.767	0.10	0.854
		trajectory	0.00	0.959	-0.08	0.566	-0.08	0.574
		23y	0.04	0.587	0.19	0.707	0.14	0.979
	Disorderliness	14y	0.01	0.943	0.06	0.956	0.05	0.923
		trajectory	0.07	0.440	-0.09	0.566	-0.19	0.391

		23y	0.11	0.095	0.03	0.796	-0.08	0.979
	Total Novelty Seeking score	14y	0.01	0.943	0.20	0.413	0.17	0.854
		trajectory	0.01	0.959	-0.24	0.349	-0.23	0.226
		23y	0.00	0.954	0.00	1.000	0.00	0.979
LEQ	Family/Parents	14y	0.02	0.105	0.16	0.275	0.13	0.840
		trajectory	0.00	0.976	-0.06	0.774	-0.06	0.860
		23y	0.04	0.673	0.05	0.776	0.01	0.943
	Accident / Illness	14y	0.10	0.001	0.13	0.345	0.03	0.867
		trajectory	-0.07	0.710	-0.15	0.638	-0.08	0.860
		23y	0.02	0.673	0.13	0.669	0.12	0.748
	Sexuality	14y	0.04	0.246	0.27	0.275	0.23	0.840
		trajectory	0.00	0.976	-0.32	0.230	-0.30	0.226
		23y	0.14	0.074	-0.14	0.776	-0.27	0.241
	Autonomy	14y	0.06	0.251	-0.08	0.275	-0.13	0.840
		trajectory	-0.05	0.710	0.09	0.774	0.14	0.860
		23y	0.05	0.673	-0.05	0.776	-0.11	0.748
	Deviance	14y	0.07	0.251	0.19	0.489	0.12	0.867
		trajectory	-0.06	0.710	-0.08	0.774	-0.02	0.860
		23y	-0.08	0.357	-0.05	0.776	0.03	0.943
	Relocation	14y	-0.05	0.251	-0.06	0.865	-0.01	0.867
		trajectory	0.03	0.791	0.11	0.774	0.08	0.860
		23y	0.02	0.673	0.26	0.277	0.24	0.241
	Distress	14y	0.13	0.038	0.28	0.275	0.14	0.840
		trajectory	-0.02	0.928	0.01	0.963	0.03	0.860
		23y	0.11	0.106	0.19	0.328	0.08	0.748
	Overall valence	14y	0.10	0.000	0.21	0.225	0.10	0.780
		trajectory	-0.05	0.322	-0.06	0.609	-0.01	0.925
		23y	0.09	0.068	0.11	0.337	0.02	0.874
PRM	Percent correct	14y	0.00	0.490	-0.01	0.837	-0.01	0.612
		trajectory	-0.03	0.562	-0.24	0.168	-0.21	0.239
		19y	-0.06	0.291	-0.29	0.115	-0.19	0.241

AGN	Total omissions for positive category	14y	0.02	0.185	0.06	0.369	0.04	0.656
		trajectory	-0.06	0.341	-0.04	0.757	0.02	0.912
		19y	0.01	0.885	0.12	0.653	0.14	0.458
	Total omissions for negative category	14y	0.02	0.185	0.16	0.151	0.15	0.398
		trajectory	-0.14	0.045	-0.31	0.090	-0.17	0.605
		19y	-0.04	0.885	0.08	0.653	0.15	0.458
SWM	Between error	14y	-0.06	0.398	0.28	0.010	0.35	0.019
		trajectory	0.06	0.370	-0.16	0.595	-0.23	0.272
		23y	0.05	0.280	0.26	0.064	0.21	0.167
	Strategy	14y	-0.01	0.398	0.20	0.062	0.21	0.138
		trajectory	0.05	0.370	0.01	0.876	-0.04	0.819
		23y	0.06	0.280	0.26	0.064	0.21	0.167
CGT	Delay aversion	14y	-0.02	0.866	0.28	0.028	0.29	0.027
		trajectory	0.08	0.262	0.03	0.951	-0.05	0.967
		23y	0.13	0.044	0.12	0.578	-0.01	0.981
	Deliberation time	14y	-0.09	0.684	0.14	0.048	0.29	0.027
		trajectory	0.13	0.208	-0.15	0.893	-0.29	0.210
		23y	0.06	0.273	-0.07	0.781	-0.11	0.981
	Overall proportion bet	14y	0.00	0.684	0.19	0.048	0.19	0.075
		trajectory	0.07	0.262	0.08	0.893	0.01	0.967
		23y	0.09	0.100	0.14	0.578	0.05	0.981
	Quality of decision making	14y	0.01	0.684	-0.37	0.000	-0.40	0.001
		trajectory	-0.11	0.231	0.27	0.572	0.40	0.065
		23y	-0.12	0.044	-0.02	0.781	0.10	0.981
	Risk adjustment	14y	-0.02	0.531	-0.50	0.000	-0.47	0.001
		trajectory	-0.08	0.262	0.02	0.986	0.10	0.967
		23y	-0.12	0.044	-0.43	0.009	-0.34	0.165
	Risk taking	14y	0.01	0.684	0.16	0.048	0.15	0.116
		trajectory	0.06	0.276	0.06	0.893	0.00	0.967
		23y	0.10	0.100	0.10	0.578	0.01	0.981
RVP	A	14y	0.00	0.575	-0.03	0.488	-0.02	0.636

		trajectory	-0.07	0.185	-0.17	0.206	-0.09	0.500
		19y	0.00	0.943	-0.17	0.226	-0.18	0.215
kirby	Estimated K	14y	0.07	0.404	0.16	0.065	0.08	0.227
		trajectory	-0.02	0.654	-0.14	0.310	-0.12	0.394
		23y	-0.01	0.782	-0.03	0.810	-0.02	0.902
	Estimated K for small LDRs	14y	0.02	0.650	0.03	0.121	0.01	0.696
		trajectory	0.04	0.869	-0.16	0.463	-0.18	0.281
		23y	0.02	0.935	-0.10	0.605	-0.12	0.614
	Esitmated K for Medium LDRs	14y	0.02	0.650	0.24	0.115	0.22	0.119
		trajectory	-0.02	0.869	-0.23	0.260	-0.21	0.281
		23y	0.00	0.935	0.07	0.605	0.08	0.614
	Estimated K for Large LDRs	14y	0.05	0.650	0.16	0.115	0.09	0.119
		trajectory	-0.01	0.884	-0.09	0.548	-0.08	0.579
		23y	0.03	0.935	-0.09	0.605	-0.12	0.614
SST	Go reaction time	14y	-0.06	0.733	0.00	0.686	0.05	0.534
		trajectory	0.00	0.970	-0.12	0.526	-0.13	0.442
		23y	-0.06	0.222	-0.13	0.312	-0.07	0.624
	Go accuracy	14y	-0.07	0.175	-0.40	0.014	-0.28	0.062
		trajectory	0.08	0.148	0.34	0.063	0.24	0.218
		23y	0.04	0.400	0.21	0.212	0.17	0.494
	Stop accuracy	14y	0.07	0.022	-0.06	0.862	-0.12	0.234
		trajectory	-0.09	0.148	-0.17	0.231	-0.09	0.569
		23y	-0.09	0.188	-0.16	0.212	-0.08	0.597
	Stop signal reaction time	14y	0.03	0.943	0.11	0.686	0.09	0.686
		trajectory	0.02	0.970	-0.11	0.526	-0.12	0.442
		23y	0.10	0.126	0.18	0.312	0.08	0.624
IED	Total trials	23y	0.10	0.278	0.23	0.372	0.09	0.675
	Total trials adjusted	23y	0.10	0.278	0.24	0.372	0.10	0.675
	PreED errors	23y	0.10	0.278	0.15	0.387	0.03	0.775
	ED errors	23y	0.06	0.609	0.14	0.387	0.07	0.675
	Total errors	23y	0.09	0.378	0.20	0.243	0.09	0.418

	Total errors adjusted	23y	0.09	0.264	0.22	0.194	0.09	0.401
SDQ	Total difficulties score	14y	0.03	0.078	0.11	0.649	0.09	0.704
		trajectory	0.02	0.699	0.14	0.296	0.13	0.364
		23y	0.08	0.090	0.28	0.043	0.18	0.186
	Emotion problems score	14y	-0.04	0.616	0.11	0.965	0.16	0.664
		trajectory	0.02	0.739	0.18	0.431	0.16	0.366
		23y	0.03	0.685	0.27	0.127	0.23	0.280
	Conduct problems score	14y	0.03	0.635	0.18	0.776	0.16	0.664
		trajectory	0.12	0.099	0.04	0.714	-0.08	0.741
		23y	0.18	0.002	0.35	0.049	0.14	0.409
	Hyperactivity score	14y	0.03	0.557	0.17	0.776	0.13	0.664
		trajectory	0.00	0.914	-0.05	0.714	-0.05	0.741
		23y	0.02	0.690	0.10	0.529	0.09	0.672
	Peer problems score	14y	0.07	0.065	-0.22	0.776	-0.27	0.249
		trajectory	-0.07	0.303	0.19	0.431	0.27	0.134
		23y	0.05	0.680	0.07	0.549	0.02	0.885
	Prosocial score	14y	0.07	0.616	0.04	0.965	-0.02	0.664
		trajectory	-0.12	0.099	0.17	0.444	0.29	0.132
		23y	-0.06	0.680	0.17	0.477	0.22	0.280
	Impact score	14y	0.02	0.616	0.03	0.965	0.01	0.664
		trajectory	-0.09	0.268	0.24	0.431	0.33	0.132
		23y	-0.03	0.690	0.28	0.127	0.31	0.209
ESPAD	Lifetime smoking	14y	0.02	0.245	0.31	0.044	0.29	0.201
		trajectory	-0.05	0.264	-0.27	0.044	-0.21	0.114
		23y	-0.07	0.165	0.00	0.895	0.08	0.617
	smoking last month	14y	0.07	0.978	0.24	0.518	0.16	0.514
		trajectory	0.20	0.061	0.04	0.761	-0.16	0.532
		23y	0.16	0.006	0.28	0.077	0.09	0.546
	Whole life drink	14y	0.02	0.806	-0.07	0.121	-0.09	0.285
		trajectory	0.02	0.640	0.07	0.580	0.05	0.713
		23y	-0.01	0.806	-0.05	0.699	-0.04	0.773

	Drink last year	14y	-0.01	0.519	-0.07	0.801	-0.06	0.762
		trajectory	0.02	0.685	0.02	0.866	0.00	0.999
		23y	0.02	0.816	-0.10	0.522	-0.12	0.440
	Drink last month	14y	-0.03	0.932	-0.01	0.671	0.01	0.858
		trajectory	-0.03	0.601	0.02	0.936	0.05	0.756
		23y	-0.01	0.749	-0.13	0.361	-0.12	0.424
DAWBA	Major Depression	14y	-0.05	0.256	-0.01	0.566	0.04	0.836
		trajectory	0.10	0.092	0.55	0.006	0.31	0.030
		23y	0.13	0.023	0.70	0.001	0.33	0.018
	ADHD (child)	14y	-0.03	0.321	0.34	0.042	0.39	0.010
		trajectory	0.01	0.693	-0.13	0.618	-0.13	0.717
		23y	0.02	0.758	-0.10	0.579	-0.12	0.491
	ADHD (parent)	14y	0.04	0.220	0.34	0.004	0.30	0.015
		16y	-0.03	0.574	0.01	0.954	0.05	0.768

All two-sided p values were adjusted for sex, intracranial volume, handedness, site. FDR test was used for multiple correction within scales.

Supplementary Table 5. The longitudinal correlation between cortical GMVs and neurocognition functions for Group 3.

ROI	SWM				CGT											
	Between error		Strategy		Delay aversion		Deliberation time		Overall proportion bet		Quality of decision making		Risk adjustment		Risk taking	
	Cor	P_{adj}	Cor	P_{adj}	Cor	P_{adj}	Cor	P_{adj}	Cor	P_{adj}	Cor	P_{adj}	Cor	P_{adj}	Cor	P_{adj}
bankssts	0.04	0.761	0.10	0.761	-0.19	0.767	-0.06	0.983	-0.03	0.983	-0.08	0.983	0.00	0.983	-0.02	0.983
caudalanteriorcingulate	-0.07	0.577	0.08	0.577	-0.13	0.960	0.02	0.960	-0.08	0.960	0.01	0.960	-0.01	0.960	-0.06	0.960
caudalmiddle-frontal	-0.17	0.162	0.18	0.162	-0.03	0.828	-0.05	0.828	-0.11	0.792	-0.07	0.828	-0.15	0.792	-0.11	0.792
cuneus	-0.14	0.497	-0.03	0.813	-0.03	0.838	0.08	0.838	-0.04	0.838	0.15	0.838	-0.04	0.838	-0.05	0.838
entorhinal	-0.14	0.406	0.10	0.406	-0.29	0.121	-0.12	0.723	-0.09	0.723	0.08	0.723	0.02	0.884	-0.07	0.723
fusiform	-0.09	0.494	0.09	0.494	-0.10	0.515	-0.10	0.515	-0.21	0.182	-0.08	0.548	-0.26	0.182	-0.23	0.182
inferiorparietal	-0.04	0.755	0.13	0.563	0.03	0.783	-0.14	0.783	-0.06	0.783	-0.05	0.783	-0.07	0.783	-0.04	0.783
Inferior-temporal	-0.14	0.347	0.12	0.347	-0.12	0.401	-0.20	0.155	-0.21	0.155	-0.08	0.549	-0.27	0.155	-0.21	0.155
isthmuscingulate	-0.17	0.370	0.08	0.502	-0.05	0.762	-0.04	0.762	-0.06	0.762	-0.11	0.762	-0.18	0.762	-0.06	0.762
lateraloccipital	-0.07	0.589	0.17	0.367	0.04	0.737	0.05	0.737	-0.15	0.657	0.08	0.737	-0.06	0.737	-0.17	0.657
lateralorbitofrontal	-0.16	0.379	0.11	0.379	-0.16	0.535	-0.03	0.823	-0.10	0.535	-0.10	0.535	-0.13	0.535	-0.11	0.535
lingual	-0.14	0.454	0.09	0.454	-0.11	0.589	0.00	0.984	-0.24	0.111	-0.08	0.599	-0.30	0.089	-0.25	0.111
medialorbitofrontal	-0.07	0.841	-0.03	0.841	-0.14	0.578	0.09	0.578	-0.06	0.632	0.21	0.535	-0.11	0.578	-0.09	0.578
middletemporal	-0.20	0.110	0.21	0.110	-0.14	0.739	-0.11	0.739	-0.02	0.933	-0.18	0.739	-0.04	0.933	-0.01	0.933
parahippocampal	-0.04	0.752	0.08	0.752	-0.26	0.196	-0.20	0.224	0.11	0.459	-0.23	0.196	-0.07	0.569	0.12	0.459
paracentral	-0.04	0.772	0.26	0.066	0.30	0.081	0.07	0.593	-0.09	0.593	0.07	0.593	0.25	0.131	-0.10	0.593
parsopercularis	-0.17	0.301	0.13	0.301	-0.05	0.798	-0.03	0.803	-0.14	0.592	-0.08	0.756	-0.22	0.426	-0.13	0.592
parsorbitalis	-0.20	0.229	0.06	0.624	-0.21	0.523	0.04	0.874	-0.06	0.874	-0.12	0.874	0.00	0.983	-0.05	0.874
parstriangularis	-0.20	0.214	-0.07	0.598	-0.15	0.786	0.10	0.786	-0.07	0.786	0.03	0.786	-0.10	0.786	-0.05	0.786
pericalcarine	-0.05	0.681	0.16	0.389	0.00	0.992	-0.19	0.557	-0.04	0.865	-0.09	0.865	-0.16	0.557	-0.06	0.865
postcentral	0.18	0.319	0.09	0.486	0.14	0.716	-0.12	0.716	0.06	0.716	-0.10	0.716	0.07	0.716	0.05	0.716
posteriorcingulate	-0.16	0.208	0.16	0.208	-0.02	0.952	-0.06	0.952	-0.17	0.552	-0.02	0.952	-0.01	0.952	-0.18	0.552
precentral	-0.08	0.544	0.12	0.544	-0.07	0.937	-0.01	0.937	0.03	0.937	-0.13	0.937	-0.04	0.937	0.04	0.937
precuneus	-0.07	0.561	0.14	0.513	-0.11	0.875	-0.02	0.875	-0.06	0.875	-0.02	0.875	-0.04	0.875	-0.06	0.875
rostralanteriorcingulate	-0.22	0.156	0.15	0.221	-0.12	0.545	0.00	0.988	-0.12	0.545	-0.09	0.545	-0.10	0.545	-0.12	0.545

rostralmiddlefrontal	-0.26	0.069	0.04	0.751	-0.02	0.870	-0.04	0.870	-0.11	0.870	-0.05	0.870	-0.04	0.870	-0.10	0.870
superiorfrontal	-0.11	0.547	0.08	0.547	0.03	0.882	0.05	0.882	-0.15	0.815	0.02	0.882	-0.03	0.882	-0.14	0.815
superiorparietal	0.05	0.920	0.01	0.920	0.02	0.858	-0.08	0.858	0.03	0.858	-0.06	0.858	0.09	0.858	0.05	0.858
superiortemporal	-0.16	0.194	0.18	0.194	-0.13	0.607	-0.18	0.450	-0.04	0.823	-0.22	0.450	-0.03	0.823	-0.03	0.823
supramarginal	0.01	0.915	0.10	0.822	0.05	0.979	-0.10	0.979	0.03	0.979	-0.09	0.979	0.04	0.979	0.00	0.979
frontalpole	-0.14	0.534	0.00	0.975	0.05	0.693	0.45	0.001	-0.16	0.288	0.26	0.073	0.27	0.073	-0.15	0.288
temporalpole	-0.13	0.597	0.03	0.782	-0.09	0.581	0.00	0.994	-0.16	0.502	-0.12	0.502	-0.22	0.477	-0.14	0.502
transversetemporal	-0.12	0.638	0.05	0.662	-0.21	0.233	0.12	0.408	-0.18	0.233	0.01	0.952	-0.18	0.233	-0.18	0.233
insula	-0.15	0.253	0.14	0.253	-0.12	0.378	-0.17	0.378	-0.11	0.378	-0.12	0.378	-0.22	0.378	-0.12	0.378

Pearson's correlation was reported. All two-sided p values (one sample t-test) were adjusted for sex, intracranial volume, handedness, site. FDR test was used for multiple correction within scales.

Supplementary Table 6. The longitudinal correlation between cortical GMVs and neurocognition functions for Group 2.

ROI	SWM				CGT											
	Between error		Strategy		Delay aversion		Deliberation time		Overall proportion bet		Quality of decision making		Risk adjustment		Risk taking	
	Cor	P_{adj}	Cor	P_{adj}	Cor	P_{adj}	Cor	P_{adj}	Cor	P_{adj}	Cor	P_{adj}	Cor	P_{adj}	Cor	P_{adj}
bankssts	-0.05	0.417	-0.01	0.889	0.02	0.810	0.04	0.513	-0.07	0.210	0.01	0.810	-0.01	0.810	-0.07	0.210
caudalanteriorcingulate	-0.06	0.251	0.04	0.263	0.00	0.976	-0.01	0.976	-0.03	0.820	0.04	0.820	-0.02	0.904	-0.03	0.820
caudalmiddle-frontal	-0.04	0.559	0.02	0.559	-0.02	0.687	0.06	0.572	0.05	0.572	-0.03	0.572	0.01	0.820	0.04	0.572
cuneus	-0.03	0.544	-0.02	0.544	0.03	0.646	0.06	0.312	-0.03	0.646	0.07	0.305	0.00	0.930	-0.02	0.652
entorhinal	-0.02	0.634	-0.02	0.634	-0.03	0.653	-0.02	0.653	-0.04	0.653	-0.02	0.653	-0.02	0.653	-0.03	0.653
fusiform	-0.03	0.655	-0.02	0.655	0.00	0.907	-0.01	0.818	-0.11	0.007	0.03	0.521	-0.04	0.452	-0.11	0.007
inferiorparietal	-0.03	0.599	-0.02	0.599	0.01	0.970	0.06	0.236	-0.06	0.236	-0.02	0.845	0.00	0.984	-0.06	0.236
Inferior-temporal	-0.05	0.262	-0.04	0.262	-0.02	0.793	-0.02	0.793	-0.10	0.022	0.00	0.931	-0.06	0.170	-0.10	0.022
isthmuscingulate	-0.06	0.227	0.02	0.515	-0.01	0.824	0.02	0.824	-0.03	0.824	-0.01	0.824	-0.01	0.824	-0.03	0.824
lateraloccipital	-0.01	0.697	0.04	0.459	0.03	0.562	0.02	0.607	-0.05	0.477	0.02	0.607	0.03	0.562	-0.05	0.477
lateralorbitofrontal	-0.10	0.014	0.02	0.591	-0.02	0.677	-0.02	0.677	-0.08	0.108	-0.02	0.677	0.02	0.677	-0.08	0.108
lingual	-0.04	0.620	0.00	0.923	0.00	0.972	0.03	0.542	-0.04	0.542	0.03	0.542	0.01	0.950	-0.04	0.542
medialorbitofrontal	-0.05	0.284	-0.02	0.527	0.04	0.527	-0.03	0.587	-0.03	0.527	0.01	0.823	0.04	0.527	-0.04	0.527
middletemporal	-0.09	0.023	0.00	0.955	-0.01	0.854	0.01	0.854	-0.07	0.130	-0.02	0.854	-0.03	0.781	-0.08	0.130
parahippocampal	-0.03	0.675	0.00	0.982	-0.04	0.640	-0.06	0.544	-0.03	0.640	0.01	0.779	-0.01	0.779	-0.03	0.640
paracentral	-0.03	0.484	0.04	0.484	-0.02	0.877	0.02	0.877	-0.01	0.877	0.01	0.877	0.01	0.877	-0.01	0.877
parsopercularis	0.00	0.992	0.04	0.591	0.02	0.919	0.01	0.919	-0.04	0.919	0.01	0.919	0.00	0.919	-0.04	0.919
parsorbitalis	-0.07	0.131	0.00	0.966	0.03	0.997	-0.02	0.997	0.00	0.997	-0.01	0.997	0.03	0.997	0.00	0.997
parstriangularis	-0.05	0.195	0.05	0.195	0.05	0.925	0.02	0.945	0.00	0.945	0.02	0.945	0.03	0.945	0.01	0.945
pericalcarine	-0.04	0.519	-0.02	0.519	-0.03	0.887	-0.01	0.887	-0.01	0.887	-0.01	0.887	-0.01	0.887	-0.01	0.887
postcentral	0.01	0.824	0.04	0.473	-0.03	0.683	0.04	0.683	-0.01	0.916	-0.05	0.683	0.00	0.916	-0.02	0.916
posteriorcingulate	-0.04	0.462	-0.02	0.653	-0.02	0.925	0.01	0.925	-0.05	0.585	0.02	0.925	0.00	0.988	-0.05	0.585
precentral	-0.02	0.607	0.03	0.607	-0.02	0.821	0.03	0.821	0.01	0.821	-0.01	0.821	0.02	0.821	0.01	0.821
precuneus	-0.08	0.056	0.03	0.457	-0.02	0.639	0.02	0.639	-0.04	0.639	-0.02	0.639	-0.02	0.639	-0.04	0.639
rostralanteriorcingulate	-0.07	0.086	0.03	0.353	0.01	0.740	-0.04	0.480	-0.04	0.480	-0.05	0.480	-0.02	0.740	-0.04	0.480

rostralmiddlefrontal	-0.07	0.141	0.03	0.448	0.00	0.904	0.04	0.705	-0.03	0.705	0.03	0.705	0.05	0.705	-0.02	0.705
superiorfrontal	-0.04	0.349	0.03	0.349	0.00	0.960	0.04	0.741	0.02	0.748	-0.02	0.748	0.04	0.741	0.02	0.748
superiorparietal	0.01	0.810	0.01	0.810	-0.03	0.727	0.06	0.704	-0.01	0.904	0.00	0.911	-0.04	0.727	-0.01	0.904
superiortemporal	-0.07	0.141	0.02	0.644	-0.03	0.770	-0.02	0.770	-0.08	0.104	-0.01	0.796	-0.02	0.770	-0.09	0.104
supramarginal	0.00	0.911	0.05	0.400	-0.03	0.972	0.05	0.972	0.00	0.972	0.00	0.972	0.01	0.972	0.01	0.972
frontalpole	0.03	0.649	0.02	0.649	-0.02	0.716	0.09	0.044	0.04	0.437	-0.01	0.857	0.10	0.044	0.04	0.466
temporalpole	-0.05	0.374	-0.01	0.734	-0.01	0.875	-0.07	0.436	-0.02	0.875	-0.01	0.875	0.04	0.857	-0.02	0.875
transversetemporal	-0.03	0.352	0.03	0.352	-0.02	0.883	-0.01	0.883	-0.04	0.712	0.02	0.883	0.00	0.942	-0.05	0.712
insula	-0.07	0.110	0.02	0.563	0.00	0.900	-0.06	0.186	0.06	0.186	-0.09	0.115	0.04	0.294	0.06	0.186

Pearson's correlation was reported. All two-sided p values (one sample t-test) were adjusted for sex, intracranial volume, handedness, site. FDR test was used for multiple correction within scales.

Supplementary Table 7. The overlapping number of participants between clustering results within different sex and the one within the whole population

	**Clustering results within the whole population**		
**Clustering results within males**			
	Group 1 (*n*=376)	Group 2 (*n*=332)	Group 3 (*n*=39)
Group 1 (*n*=380)	374	6	0
Group 2 (*n*=335)	2	326	7
Group 3 (*n*=32)	0	0	32
**Clustering results within females**			
	Group 1 (*n*=335)	Group 2 (*n*=433)	Group 3 (*n*=28)
Group 1 (*n*=333)	327	6	0
Group 2 (*n*=425)	8	417	0
Group 3 (*n*=38)	0	10	28

Supplementary Table 8. Full comparison of Group 2 and Group 3 vs Group 1 clustered within sex in terms of the personal traits, environmental burden, neuro-cognition, behavioral risk factors and mental symptoms at age 14, longitudinal trajectory and at age 23.

Questionnaire	Item	Time	Clustering results within males						Clustering results within females					
			Group 2 vs Group 1		Group 3 vs Group 1		Group 3 vs Group 2		Group 2 vs Group 1		Group 3 vs Group 1		Group 3 vs Group 2	
			Cohen's d	P_{adj}	*Cohen's d*	P_{adj}	*Cohen's d*	P_{adj}	*Cohen's d*	P_{adj}	*Cohen's d*	P_{adj}	*Cohen's d*	P_{adj}
NEO	Neuroticism	14y	0.00	0.793	-0.14	0.798	-0.15	0.564	0.03	0.548	-0.14	0.796	-0.17	0.603
		trajectory	-0.02	0.854	0.02	0.915	0.04	0.848	0.06	0.591	0.14	0.909	0.08	0.811
		23y	0.06	0.429	0.00	0.933	-0.07	0.861	0.13	0.220	0.06	0.983	-0.07	0.943
	Extraversion	14y	0.01	0.903	0.45	0.053	0.45	0.153	0.00	0.548	0.12	0.796	0.11	0.626
		trajectory	-0.15	0.210	-0.31	0.283	-0.19	0.464	-0.02	0.783	-0.19	0.909	-0.20	0.521
		23y	-0.15	0.241	-0.01	0.933	0.15	0.861	-0.04	0.594	-0.25	0.598	-0.21	0.943
	Openness	14y	-0.05	0.793	0.00	0.798	0.05	0.978	0.05	0.548	-0.25	0.100	-0.29	0.264
		trajectory	-0.04	0.854	-0.20	0.518	-0.17	0.464	-0.09	0.591	0.14	0.909	0.21	0.521
		23y	-0.06	0.429	-0.22	0.933	-0.14	0.861	0.00	0.961	0.01	0.983	0.01	0.963
	Agreeableness	14y	-0.06	0.793	0.11	0.798	0.17	0.564	-0.02	0.548	-0.28	0.300	-0.26	0.264
		trajectory	-0.04	0.854	0.19	0.518	0.25	0.464	-0.23	0.013	0.09	0.909	0.30	0.521
		23y	-0.12	0.241	0.16	0.933	0.27	0.861	-0.24	0.005	-0.16	0.598	0.09	0.943
	Conscientiousness	14y	0.00	0.903	-0.12	0.798	-0.11	0.564	0.01	0.548	0.08	0.796	0.07	0.810
		trajectory	-0.13	0.325	0.09	0.915	0.21	0.464	-0.05	0.591	-0.01	0.909	0.05	0.838
		23y	-0.12	0.241	-0.06	0.933	0.05	0.861	-0.09	0.395	0.05	0.983	0.14	0.943
TCI	Exploratory excitability	14y	0.10	0.534	0.11	0.224	0.01	0.953	-0.02	0.532	0.18	0.992	0.19	0.723
		trajectory	-0.17	0.132	-0.16	0.646	0.01	0.930	-0.01	0.996	0.00	0.994	0.01	0.949
		23y	-0.10	0.479	-0.06	0.788	0.04	0.906	-0.09	0.635	0.05	0.924	0.13	0.805
	Impulsiveness	14y	0.05	0.534	0.26	0.062	0.21	0.941	-0.07	0.532	0.14	0.992	0.22	0.723
		trajectory	0.00	0.924	-0.20	0.646	-0.21	0.930	0.06	0.996	-0.04	0.994	-0.09	0.793
		23y	0.00	0.974	-0.06	0.788	-0.07	0.906	-0.04	0.867	0.05	0.924	0.09	0.805
	Extravagance	14y	0.04	0.540	0.15	0.319	0.11	0.941	-0.02	0.996	0.00	0.992	0.02	0.831
		trajectory	0.00	0.924	0.11	0.646	0.11	0.930	0.05	0.996	-0.30	0.375	-0.32	0.221
		23y	0.05	0.527	0.40	0.196	0.34	0.492	0.09	0.635	-0.05	0.924	-0.13	0.805
	Disorderliness	14y	0.07	0.634	0.10	0.883	0.03	0.953	-0.02	0.532	0.07	0.992	0.09	0.723

		trajectory	0.09	0.530	0.09	0.646	0.00	0.930	0.01	0.996	-0.28	0.375	-0.30	0.221
		23y	0.20	0.035	0.29	0.196	0.10	0.906	0.02	0.867	-0.13	0.924	-0.14	0.805
	Total Novelty Seeking	14y	0.10	0.534	0.27	0.100	0.14	0.941	-0.05	0.532	0.15	0.992	0.21	0.723
	score	trajectory	-0.04	0.924	-0.08	0.646	-0.04	0.930	0.05	0.996	-0.25	0.375	-0.27	0.221
		23y	0.05	0.527	0.21	0.436	0.17	0.906	-0.01	0.867	-0.02	0.924	-0.01	0.961
LEQ	Family/Parents	14y	-0.05	0.942	-0.03	0.995	0.03	0.901	0.06	0.040	0.30	0.066	0.26	0.371
		trajectory	0.03	0.864	-0.06	0.849	-0.09	0.880	-0.02	0.877	-0.08	0.695	-0.05	0.991
		23y	0.06	0.894	-0.13	0.894	-0.18	0.838	0.03	0.698	0.21	0.371	0.16	0.465
	Accident / Illness	14y	0.03	0.942	0.17	0.986	0.14	0.757	0.17	0.000	0.20	0.044	0.02	0.845
		trajectory	-0.06	0.864	-0.22	0.616	-0.16	0.876	-0.09	0.495	-0.07	0.695	0.03	0.991
		23y	-0.02	0.977	0.11	0.894	0.16	0.838	0.07	0.610	0.21	0.335	0.15	0.465
	Sexuality	14y	-0.01	0.942	0.09	0.995	0.09	0.901	0.11	0.005	0.60	0.001	0.48	0.029
		trajectory	-0.03	0.864	-0.34	0.616	-0.26	0.581	0.02	0.947	-0.44	0.067	-0.44	0.072
		23y	0.01	0.977	-0.31	0.894	-0.32	0.838	0.27	0.003	-0.02	0.710	-0.28	0.422
	Autonomy	14y	-0.02	0.942	-0.14	0.659	-0.10	0.757	0.11	0.080	0.10	0.858	-0.01	0.845
		trajectory	0.00	0.943	0.16	0.696	0.14	0.876	-0.06	0.616	-0.06	0.695	0.00	0.991
		23y	0.06	0.894	0.06	0.894	0.00	0.954	0.06	0.653	-0.17	0.456	-0.26	0.422
	Deviance	14y	0.03	0.942	0.19	0.995	0.15	0.901	0.10	0.014	0.42	0.024	0.29	0.371
		trajectory	0.02	0.864	0.06	0.849	0.04	0.889	-0.15	0.258	-0.48	0.052	-0.31	0.206
		23y	-0.03	0.977	0.11	0.894	0.14	0.838	-0.14	0.223	-0.26	0.230	-0.12	0.465
	Relocation	14y	-0.10	0.942	0.25	0.245	0.37	0.040	-0.02	0.111	-0.21	0.415	-0.20	0.110
		trajectory	0.06	0.864	-0.24	0.616	-0.34	0.581	0.02	0.838	0.46	0.052	0.45	0.072
		23y	-0.01	0.977	0.06	0.894	0.07	0.838	0.04	0.653	0.40	0.119	0.36	0.200
	Distress	14y	0.01	0.942	0.05	0.995	0.03	0.901	0.22	0.000	0.59	0.003	0.37	0.255
		trajectory	0.06	0.864	0.02	0.849	-0.04	0.889	-0.09	0.495	-0.07	0.695	0.02	0.991
		23y	0.12	0.894	-0.02	0.926	-0.14	0.838	0.11	0.264	0.28	0.230	0.17	0.465
	Overall valence	14y	-0.04	0.767	0.10	0.889	0.12	0.626	0.21	0.000	0.51	0.000	0.31	0.122
		trajectory	0.02	0.808	-0.12	0.585	-0.13	0.501	-0.11	0.135	-0.15	0.365	-0.04	0.793
		23y	0.08	0.337	-0.04	0.962	-0.11	0.626	0.13	0.077	0.19	0.219	0.07	0.665
PRM	Percent correct	14y	-0.09	0.648	0.23	0.254	0.32	0.164	0.03	0.506	-0.33	0.034	-0.37	0.013
		trajectory	0.04	0.824	-0.87	0.009	-0.82	0.007	-0.01	0.943	0.08	0.739	0.08	0.699

		19y	-0.02	0.722	-0.58	0.119	-0.37	0.148	-0.07	0.344	-0.20	0.273	-0.13	0.487
AGN	Total omissions for positive category	14y	0.08	0.138	-0.03	0.915	-0.12	0.816	-0.08	0.914	0.24	0.036	0.33	0.034
		trajectory	-0.09	0.290	-0.11	0.559	-0.01	0.887	-0.03	0.744	-0.19	0.408	-0.18	0.485
		19y	0.03	0.896	-0.14	0.627	-0.17	0.651	-0.02	0.855	0.28	0.427	0.38	0.344
	Total omissions for negative category	14y	0.07	0.138	0.10	0.795	0.04	0.816	-0.08	0.914	0.31	0.024	0.41	0.013
		trajectory	-0.14	0.186	-0.34	0.213	-0.20	0.686	-0.09	0.592	-0.50	0.059	-0.45	0.158
		19y	-0.01	0.896	-0.10	0.627	-0.12	0.651	-0.04	0.855	0.17	0.462	0.27	0.351
SWM	Between error	14y	-0.07	0.866	0.22	0.432	0.31	0.379	-0.04	0.364	0.38	0.002	0.42	0.015
		trajectory	-0.01	0.783	-0.32	0.258	-0.30	0.295	0.11	0.177	-0.14	0.780	-0.27	0.346
		23y	-0.02	0.846	0.10	0.650	0.12	0.579	0.13	0.076	0.23	0.237	0.11	0.967
	Strategy	14y	0.01	0.719	0.11	0.498	0.11	0.781	-0.02	0.492	0.32	0.021	0.33	0.045
		trajectory	-0.10	0.402	-0.05	0.722	0.06	0.857	0.20	0.017	0.01	0.780	-0.20	0.346
		23y	-0.08	0.728	0.26	0.588	0.33	0.273	0.18	0.025	0.17	0.237	-0.01	0.967
CGT	Delay aversion	14y	0.05	0.893	-0.01	0.797	-0.07	0.962	-0.09	0.672	0.54	0.004	0.63	0.004
		trajectory	0.00	0.976	0.16	0.871	0.16	0.498	0.15	0.509	-0.04	0.993	-0.19	0.749
		23y	0.18	0.046	0.12	0.941	-0.07	0.828	0.11	0.196	0.18	0.539	0.06	0.830
	Deliberation time	14y	-0.21	0.145	0.07	0.210	0.36	0.038	0.03	0.672	0.03	0.615	0.00	0.885
		trajectory	0.23	0.052	-0.05	0.952	-0.36	0.304	0.03	0.896	-0.15	0.993	-0.12	0.749
		23y	-0.02	0.762	-0.10	0.941	-0.08	0.828	0.12	0.196	-0.22	0.639	-0.21	0.660
	Overall proportion bet	14y	-0.04	0.893	0.40	0.034	0.43	0.038	-0.02	0.672	0.08	0.265	0.10	0.401
		trajectory	0.20	0.055	-0.13	0.871	-0.33	0.251	-0.04	0.896	0.03	0.993	0.07	0.749
		23y	0.20	0.028	0.02	0.941	-0.18	0.828	0.01	0.891	0.14	0.539	0.13	0.718
	Quality of decision making	14y	0.00	0.931	-0.31	0.023	-0.34	0.022	0.05	0.672	-0.33	0.006	-0.39	0.011
		trajectory	-0.18	0.063	0.23	0.871	0.43	0.251	-0.08	0.896	0.17	0.993	0.25	0.749
		23y	-0.11	0.196	0.02	0.941	0.13	0.828	-0.12	0.196	-0.12	0.539	0.00	0.945
	Risk adjustment	14y	0.04	0.893	-0.47	0.018	-0.50	0.022	-0.02	0.672	-0.50	0.004	-0.46	0.011
		trajectory	-0.20	0.055	0.13	0.871	0.35	0.251	0.01	0.941	-0.06	0.993	-0.07	0.749
		23y	-0.12	0.146	-0.28	0.755	-0.18	0.828	-0.11	0.196	-0.46	0.072	-0.35	0.391
	Risk taking	14y	-0.03	0.893	0.34	0.036	0.35	0.063	-0.01	0.672	0.06	0.277	0.07	0.421
		trajectory	0.19	0.055	-0.19	0.871	-0.37	0.251	-0.04	0.896	0.06	0.993	0.10	0.749
		23y	0.22	0.028	-0.05	0.941	-0.28	0.828	0.01	0.891	0.14	0.539	0.14	0.718

RVP	A	14y	0.00	0.858	0.09	0.551	0.09	0.613	0.03	0.573	-0.16	0.076	-0.19	0.148
		trajectory	0.01	0.974	-0.23	0.269	-0.25	0.252	-0.14	0.110	0.06	0.934	0.19	0.383
		19y	0.04	0.654	-0.06	0.831	-0.10	0.646	0.00	0.822	-0.31	0.102	-0.32	0.097
kirby	Estimated K	14y	0.11	0.237	0.15	0.402	0.03	0.688	0.03	0.938	0.03	0.276	0.00	0.656
		trajectory	-0.04	0.553	-0.10	0.556	-0.07	0.728	0.00	0.981	0.02	0.923	0.02	0.928
		23y	-0.02	0.806	0.06	0.810	0.09	0.718	0.01	0.943	0.00	0.988	-0.01	0.959
	Estimated K for small LDRs	14y	0.04	0.813	0.02	0.326	-0.02	0.948	-0.02	0.963	0.03	0.724	0.05	0.876
		trajectory	0.11	0.558	-0.04	0.969	-0.14	0.729	0.02	0.980	-0.09	0.966	-0.11	0.793
		23y	0.07	0.839	0.07	0.983	0.00	0.954	0.00	0.951	-0.03	0.957	-0.02	0.900
	Estimated K for Medium LDRs	14y	0.07	0.769	0.04	0.804	-0.02	0.948	-0.03	0.963	0.15	0.685	0.18	0.534
		trajectory	-0.02	0.834	-0.08	0.969	-0.06	0.729	-0.04	0.980	0.02	0.966	0.05	0.793
		23y	0.01	0.839	0.15	0.983	0.14	0.954	0.00	0.951	0.11	0.957	0.11	0.900
	Estimated K for Large LDRs	14y	0.09	0.769	0.17	0.326	0.07	0.948	0.02	0.963	0.05	0.685	0.02	0.534
		trajectory	-0.01	0.834	-0.10	0.969	-0.10	0.729	0.00	0.980	0.06	0.966	0.05	0.793
		23y	0.03	0.839	-0.01	0.983	-0.03	0.954	0.04	0.951	0.00	0.957	-0.04	0.900
SST	Go reaction time	14y	-0.04	0.965	-0.05	0.667	-0.01	0.642	-0.09	0.201	-0.03	0.946	0.06	0.521
		trajectory	-0.04	0.631	-0.13	0.534	-0.10	0.662	0.06	0.463	-0.18	0.453	-0.23	0.464
		23y	-0.10	0.229	-0.26	0.238	-0.23	0.675	0.00	0.952	-0.01	0.980	-0.01	0.956
	Go accuracy	14y	0.00	0.913	-0.31	0.229	-0.31	0.241	-0.12	0.076	-0.43	0.103	-0.19	0.612
		trajectory	-0.02	0.935	0.32	0.325	0.35	0.282	0.19	0.047	-0.10	0.838	-0.26	0.442
		23y	0.04	0.554	0.29	0.155	0.26	0.307	0.07	0.576	-0.21	0.406	-0.30	0.254
	Stop accuracy	14y	0.06	0.226	-0.02	0.912	-0.09	0.635	0.06	0.077	0.08	0.649	0.01	0.721
		trajectory	-0.14	0.181	-0.08	0.572	0.06	0.894	-0.01	0.896	-0.16	0.838	-0.15	0.455
		23y	-0.12	0.180	-0.30	0.155	-0.23	0.307	-0.05	0.576	0.17	0.406	0.23	0.254
	Stop signal reaction time	14y	0.06	0.773	0.22	0.517	0.17	0.642	0.00	0.495	0.20	0.946	0.20	0.521
		trajectory	-0.07	0.631	-0.25	0.476	-0.18	0.662	0.11	0.302	0.25	0.381	0.14	0.464
		23y	0.09	0.229	0.03	0.754	-0.06	0.822	0.09	0.382	0.53	0.005	0.48	0.019
IED	Total trials	23y	0.14	0.283	0.13	0.812	-0.02	0.993	0.07	0.931	0.43	0.197	0.30	0.293
	Total trials adjusted	23y	0.16	0.283	0.14	0.812	-0.04	0.993	0.09	0.921	0.39	0.197	0.20	0.293
	PreED errors	23y	0.07	0.371	0.00	0.944	-0.07	0.993	0.15	0.736	0.47	0.197	0.22	0.293
	ED errors	23y	0.10	0.292	0.06	0.812	-0.04	0.993	0.02	0.931	0.13	0.716	0.11	0.604

	Total errors	23y	0.10	0.292	0.02	0.819	-0.08	0.993	0.06	0.974	0.40	0.197	0.30	0.293
	Total errors adjusted	23y	0.14	0.283	0.08	0.812	-0.07	0.993	0.08	0.921	0.37	0.197	0.20	0.293
SDQ	Total difficulties score	14y	-0.06	0.817	0.05	0.706	0.12	0.578	0.12	0.015	0.11	0.564	0.00	0.968
		trajectory	0.15	0.055	0.17	0.275	0.03	0.788	-0.06	0.432	0.17	0.406	0.23	0.223
		23y	0.09	0.201	0.22	0.220	0.13	0.492	0.08	0.212	0.33	0.071	0.22	0.195
	Emotion problems score	14y	-0.07	0.930	0.16	0.413	0.23	0.385	-0.01	0.391	-0.07	0.849	-0.06	0.912
		trajectory	0.07	0.378	-0.03	0.928	-0.11	0.934	0.03	0.664	0.31	0.238	0.27	0.262
		23y	0.02	0.723	0.09	0.764	0.07	0.834	0.06	0.864	0.26	0.194	0.21	0.355
	Conduct problems score	14y	-0.03	0.930	0.18	0.413	0.22	0.385	0.08	0.270	0.23	0.814	0.15	0.912
		trajectory	0.20	0.078	0.11	0.928	-0.08	0.934	0.04	0.664	0.05	0.793	0.02	0.982
		23y	0.14	0.286	0.44	0.184	0.24	0.713	0.22	0.013	0.37	0.051	0.13	0.468
	Hyperactivity score	14y	-0.01	0.930	0.21	0.413	0.23	0.385	0.07	0.158	0.10	0.814	0.02	0.912
		trajectory	0.07	0.378	0.07	0.928	0.00	0.934	-0.07	0.564	-0.06	0.793	0.00	0.982
		23y	0.05	0.583	0.19	0.764	0.17	0.834	0.02	0.942	0.08	0.669	0.06	0.740
	Peer problems score	14y	-0.06	0.930	-0.49	0.301	-0.43	0.078	0.18	0.014	0.09	0.866	-0.09	0.912
		trajectory	0.06	0.378	0.33	0.495	0.26	0.721	-0.18	0.135	0.09	0.793	0.27	0.287
		23y	0.08	0.583	-0.04	0.954	-0.11	0.834	0.01	0.942	0.27	0.194	0.25	0.353
	Prosocial score	14y	0.03	0.930	0.14	0.413	0.11	0.540	0.15	0.380	0.06	0.895	-0.10	0.912
		trajectory	-0.11	0.378	-0.02	0.928	0.08	0.934	-0.14	0.243	0.35	0.238	0.50	0.061
		23y	-0.06	0.583	-0.09	0.764	-0.04	0.834	-0.01	0.942	0.52	0.051	0.49	0.047
	Impact score	14y	-0.02	0.930	-0.15	0.413	-0.12	0.432	0.08	0.168	0.22	0.849	0.11	0.912
		trajectory	-0.09	0.378	0.15	0.928	0.25	0.721	-0.09	0.564	0.31	0.238	0.39	0.106
		23y	-0.12	0.394	0.13	0.764	0.29	0.713	0.04	0.864	0.45	0.051	0.38	0.100
ESPAD	Lifetime smoking	14y	0.09	0.521	0.37	0.228	0.27	0.491	-0.02	0.312	0.41	0.003	0.46	0.032
		trajectory	-0.04	0.546	-0.28	0.137	-0.23	0.205	-0.09	0.214	-0.27	0.130	-0.18	0.308
		23y	-0.06	0.442	0.12	0.670	0.17	0.428	-0.08	0.266	-0.02	0.824	0.07	0.757
	smoking last month	14y	0.15	0.874	0.28	0.829	0.11	0.955	-0.09	0.359	0.16	0.379	0.24	0.236
		trajectory	0.42	0.009	0.10	0.667	-0.31	0.391	0.00	0.998	0.07	0.845	0.06	0.831
		23y	0.24	0.008	0.10	0.443	-0.13	0.646	0.08	0.303	0.34	0.148	0.21	0.306
	Whole life drink	14y	-0.04	0.007	0.03	0.605	0.06	0.917	0.10	0.037	0.08	0.508	-0.02	0.956
		trajectory	0.14	0.056	0.18	0.252	0.04	0.738	-0.16	0.029	-0.20	0.226	-0.04	0.778

		23y	0.08	0.249	0.29	0.112	0.25	0.260	-0.10	0.150	-0.28	0.126	-0.15	0.361
	Drink last year	14y	-0.06	0.006	-0.01	0.984	0.06	0.758	0.09	0.087	0.06	0.173	-0.03	0.659
		trajectory	0.11	0.129	0.27	0.121	0.16	0.350	-0.13	0.056	-0.33	0.051	-0.21	0.243
		23y	-0.01	0.986	0.19	0.392	0.19	0.369	0.02	0.920	-0.29	0.113	-0.34	0.084
	Drink last month	14y	-0.08	0.039	0.05	0.640	0.14	0.941	0.07	0.096	0.09	0.185	0.02	0.585
		trajectory	0.03	0.602	0.25	0.197	0.22	0.271	-0.14	0.060	-0.27	0.114	-0.16	0.425
		23y	-0.07	0.431	0.13	0.617	0.19	0.380	0.04	0.693	-0.28	0.152	-0.35	0.086
DAWBA	Major Depression	14y	0.17	0.761	0.07	0.956	-0.10	0.771	-0.13	0.619	0.11	0.960	0.30	0.639
		trajectory	0.10	0.275	-0.10	0.881	-0.13	0.594	0.11	0.140	0.76	0.000	0.64	0.001
		23y	0.22	0.015	-0.14	0.975	-0.23	0.374	0.06	0.345	0.82	0.000	0.58	0.001
	ADHD (child)	14y	-0.05	0.036	0.31	0.435	0.40	0.082	0.00	0.351	0.24	0.019	0.23	0.068
		trajectory	0.07	0.457	-0.20	0.378	-0.31	0.231	0.03	0.781	-0.31	0.158	-0.33	0.118
		23y	-0.08	0.333	-0.01	0.882	0.08	0.824	0.10	0.165	-0.02	0.953	-0.11	0.581
	ADHD (parent)	14y	0.06	0.155	0.35	0.030	0.28	0.105	0.04	0.727	0.28	0.120	0.23	0.145
		16y	-0.01	0.982	0.19	0.328	0.23	0.310	-0.02	0.855	0.25	0.166	0.28	0.128

All two-sided p values were adjusted for intracranial volume, handedness, site. FDR test was used for multiple correction within scales.

Supplementary Table 9. Comparison of PRS for attention-deficit/hyperactivity disorder (ADHD), austism disorder (ASD), educational attainment (EA) and IQ among three groups.

**Term**	**Group 2 vs Group 1**		**Group 3 vs Group 1**		**Group 3 vs Group 2**	
	t	P_{adj}	***t***	P_{adj}	***t***	P_{adj}
ADHD	-0.799	0.424	2.709	0.007	2.392	0.017
ASD	-0.359	0.720	0.476	0.634	0.623	0.533
EA	-0.426	0.670	1.105	0.270	1.281	0.200
IQ	1.579	0.115	0.656	0.512	0.015	0.988

Two-sided t-test was used and BH-FDR method was used for multiple correction within one phenotype.

Supplementary Table 10. Correlation between Group3-reweighted GMV and neurocognition in ABCD.

Questionnaire	Time	Correlation	P_{adj}
Game of Dice Task	Safe times (GDT_Safe)	-0.07	2.77E-06
	Risky times (GDT_Risky)	0.07	3.28E-06
	Safe vs risky	-0.07	2.18E-10
Delay Discounting Task	mean RT for immediate choices (DDT_immed)	-0.04	0.057
	mean RT for delayed choices (DDT_delayed)	0.05	7.25E-04
NIH Toolbox	PVT uncorrected (PVT)	-0.12	1.78E-58
	Flanker uncorrected (Flanker)	-0.01	1.10E-08
	List sorting uncorrected (List)	-0.09	9.86E-38
	DCCS uncorrected (DCCS)	-0.06	1.04E-11
	Pattern comparison uncorrected (Pattern)	-0.01	0.121
	PSMT uncorrected (PSMT)	-0.02	1.11E-04
	Reading uncorrected (Reading)	-0.13	3.05E-59
	Fluid cognition uncorrected (FluidCog)	-0.06	2.16E-19
	Crystallized cognition uncorrected (CrystalCog)	-0.15	8.94E-78
	Total cognition function uncorrected (TotalCog)	-0.13	2.36E-51

Pearson's correlation was reported. All two-sided p values (one sample t-test) were adjusted for sex, handedness, scanner. FDR test was used for multiple correction within scales. PVT, Picture Vocabulary Test; Flanker, Flanker Inhibitory Control and Attention Test; List sorting, List Sorting Working Memory Test; DCCS, Dimensional Change Card Sort Test; Pattern comparison, Pattern Comparison Processing Speed Test; PSMT, Picture Sequence Memory Test; Reading, Oral Reading Recognition Test

Supplementary Table 11. SNPs with significant genome-wise significant effects in Group 3 GWAS.

CHR	SNP	BP	Gene	Risk allele	*BETA*	*P*
6	rs9375442	126481991	CENPW: Non-coding Transcript Variant	A	0.51	9.25E-09
6	rs373980643	126378465	CENPW	GT	0.52	1.19E-08
6	rs576049	126409397	CENPW	T	0.51	1.25E-08
6	rs9321065	126422941	CENPW	G	0.51	1.27E-08
6	rs4559102	126452434	CENPW	G	0.51	1.29E-08
6	rs2152876	126440082	CENPW	G	0.51	1.70E-08
6	6:126419677	126419677	CENPW	TTA	0.51	1.71E-08
6	rs4897181	126432418	CENPW	C	0.51	1.72E-08
6	rs1042418304	126406269	CENPW	A	0.51	1.74E-08
6	rs9388496	126463916	CENPW	A	0.51	1.74E-08
6	rs2039735	126423930	CENPW	T	0.51	1.84E-08
6	rs2326387	126359343	CENPW	G	0.51	1.99E-08
6	rs1415671	126438441	CENPW	T	0.51	2.02E-08
6	rs572470766	126438495	CENPW	G	0.51	2.03E-08
6	rs4897182	126434496	CENPW	T	0.51	2.08E-08
6	rs36081436	126459070	CENPW	C	0.51	2.09E-08
6	rs1286935990	126364681	CENPW	G	0.51	2.11E-08
6	rs200727626	126345679	CENPW	A	0.50	2.15E-08
6	rs1361109	126449997	CENPW	C	0.50	2.37E-08
6	rs1361262	126380821	CENPW	T	0.50	2.42E-08
6	rs9372840	126501489	CENPW	A	0.51	2.60E-08
6	rs2326451	126490782	None*	A	0.51	2.65E-08
6	rs4895807	126365367	CENPW	C	0.49	2.89E-08
6	rs59698523	126411948	CENPW	T	0.50	2.91E-08
6	rs2130603	126505673	None*	A	0.50	3.08E-08
6	rs1572569	126450541	CENPW	G	0.50	3.10E-08
6	rs915273902	126369111	CENPW	AT	0.50	3.29E-08
6	rs1844594	126466961	CENPW	G	0.50	3.30E-08

6	rs9388495	126461311	CENPW	G	0.50	4.02E-08
6	rs4897178	126406762	CENPW	T	0.49	4.31E-08

One sample two-tailed t-test was used in the logistic regression model to test for coefficients.

* SNP is in the intergenic upstream/downstream district of CENPW.

Supplementary Table 12. Mediation effect of different environmental factors on peak GMV through cg06064461 in IMAGEN.

Questionnaire	Item	Total effect	P_{unadj}	P_{adj}	Indirect effect	P_{unadj}	P_{adj}	Mediation proportion
Childhood Trauma Questionnaire	Emotional abuse	-0.02	0.045	0.118	-5.00E-05	0.928	0.929	0.23%
	Physical abuse	-0.02	0.521	0.521	4.22E-04	0.753	0.929	-2.65%
	Sexual abuse	-0.02	0.238	0.298	-6.07E-04	0.577	0.929	2.51%
	Emotional neglect	-0.02	0.048	0.118	4.59E-05	0.929	0.929	-0.23%
	Physical neglect	-0.03	0.071	0.118	-1.71E-04	0.852	0.929	0.49%
Family Stresses	Socioeconomics	-0.06	0.054	0.111	-1.08E-03	0.584	0.673	1.75%
	Work	0.07	0.055	0.111	-1.45E-03	0.440	0.673	-2.22%
	Health	-0.05	0.185	0.247	8.29E-04	0.673	0.673	-1.52%
	Addiction	0.01	0.806	0.806	2.96E-03	0.346	0.673	25.90%
Childs experience of family life	Affirmation	0.05	0.013	0.027	4.84E-03	0.048	0.191	8.96%
	Discipline	-0.07	0.004	0.016	-5.43E-04	0.672	0.896	0.79%
	Rules	-0.04	0.207	0.207	1.65E-04	0.913	0.913	-0.46%
	Special allowance	-0.03	0.170	0.207	-1.23E-03	0.384	0.767	4.04%

All two-sided p values (one sample t-test) were adjusted for sex, handedness, site. FDR test was used for multiple correction within scales.

Supplementary Table 13. Correlations between PRS of delayed neurodevelopment and baseline brain region GMVs in UKB.

ROI	Correlation	P_{adj}
**Cortical regions**		
bankssts	-0.05	3.85E-15
caudalanteriorcingulate	-0.04	1.26E-09
caudalmiddlefrontal	-0.06	2.00E-21
cuneus	-0.03	3.52E-09
entorhinal	-0.02	5.04E-05
fusiform	-0.07	6.66E-32
inferiorparietal	-0.04	2.52E-13
inferiortemporal	-0.06	1.27E-27
isthmuscingulate	-0.05	2.65E-17
lateraloccipital	-0.06	1.32E-22
lateralorbitofrontal	-0.07	1.11E-29
lingual	-0.03	1.61E-08
medialorbitofrontal	-0.07	2.12E-29
middletemporal	-0.06	4.09E-26
parahippocampal	-0.02	1.47E-04
paracentral	-0.04	1.75E-14
parsopercularis	-0.04	1.51E-13
parsorbitalis	-0.05	7.04E-20
parstriangularis	-0.04	9.44E-14
pericalcarine	-0.02	6.29E-04
postcentral	-0.05	6.22E-20
posteriorcingulate	-0.05	1.49E-20
precentral	-0.05	2.53E-20
precuneus	-0.05	6.74E-21
rostralanteriorcingulate	-0.06	9.91E-29
rostralmiddlefrontal	-0.07	4.85E-32
superiorfrontal	-0.06	1.08E-26

superiorparietal	-0.05	5.08E-20
superiortemporal	-0.06	2.63E-28
supramarginal	-0.05	6.03E-19
frontalpole	-0.04	2.10E-10
transversetemporal	-0.04	7.20E-12
insula	-0.06	1.67E-23
**Subcortical regions**		
thalamus_proper	-0.05	7.04E-20
caudate	-0.03	2.43E-09
putamen	-0.03	1.38E-08
pallidum	-0.04	2.27E-10
hippocampus	-0.04	5.28E-14
amygdala	-0.05	2.40E-17
accumbens_area	-0.04	1.52E-11
**Summary volume**		
subcorticalGMV	-0.06	2.27E-21
coticalGMV	-0.08	3.31E-42
totalGMV	-0.08	3.31E-42

Pearson's correlation was reported. All two-sided p values (one sample t-test) were adjusted for sex, age at recruitment and site. FDR test was used for multiple correction.

Supplementary Table 14. Correlations between CENPW score of delayed neurodevelopment and baseline brain region GMVs in UKB.

ROI	Correlation	P_{adj}
**Cortical regions**		
bankssts	-0.02	5.28E-03
caudalanteriorcingulate	-0.02	2.10E-03
caudalmiddlefrontal	-0.05	3.47E-19
cuneus	0.00	6.46E-01
entorhinal	-0.01	1.45E-01
fusiform	-0.03	1.11E-06
inferiorparietal	-0.02	1.12E-04
inferiortemporal	-0.02	1.12E-04
isthmuscingulate	-0.02	2.08E-03
lateraloccipital	-0.01	4.58E-02
lateralorbitofrontal	-0.06	4.20E-25
lingual	-0.01	2.08E-02
medialorbitofrontal	-0.04	2.08E-13
middletemporal	-0.03	2.06E-05
parahippocampal	-0.01	3.24E-01
paracentral	-0.02	1.38E-04
parsopercularis	-0.02	2.08E-03
parsorbitalis	-0.04	1.12E-10
parstriangularis	-0.03	1.41E-07
pericalcarine	0.00	6.85E-01
postcentral	-0.03	2.18E-06
posteriorcingulate	-0.02	4.00E-03
precentral	-0.04	5.27E-09
precuneus	-0.02	1.12E-04
rostralanteriorcingulate	-0.05	1.60E-14
rostralmiddlefrontal	-0.05	4.38E-17
superiorfrontal	-0.05	1.36E-15

superiorparietal	-0.02	2.08E-03
superiortemporal	-0.02	1.12E-04
supramarginal	-0.02	2.80E-04
frontalpole	-0.01	1.24E-01
transversetemporal	-0.02	1.16E-02
insula	-0.05	4.38E-17
**Subcortical regions**		
thalamus_proper	-0.04	8.30E-11
caudate	-0.03	1.95E-05
putamen	-0.03	2.21E-06
pallidum	-0.04	1.27E-09
hippocampus	-0.02	1.85E-03
amygdala	-0.01	6.74E-02
accumbens_area	-0.01	9.29E-02
**Summary volume**		
subcorticalGMV	-0.04	1.18E-11
coticalGMV	-0.04	3.30E-13
totalGMV	-0.05	3.74E-14

Pearson’s correlation was reported. All two-sided p values (one sample t-test) were adjusted for sex, age at recruitment and site. FDR test was used for multiple correction.

Supplementary Table 15. Correlations between genetic risk score of delayed neurodevelopment and neurocognition in UKB.

**Neurocognition tests**	**whole-genome PRS**	**CENPW score**
Numeric memory	-0.015 (*p*=0.005)	-0.0003 (*p*=0.950)
Trail making	-0.006 (*p*=0.344)	0.007 (*p*=0.244)
Tower rearranging test	0.003 (*p*=0.679)	-0.007 (*p*=0.284)

One sample two-sided t-test was used to evaluate the significance of correlations. Whole-genome PRS refers to the average of PRS across different *p* thresholds.

Supplementary Table 16. Demographics and baseline characteristics for all adolescents in IMAGEN and those included in the analyses.

**Variable**	**all adolescents in IMAGEN**	**adolescents selected**	**statistics* (*P*-value)**
N	2138	1543	
Baseline Age (years; mean ± s.d.)	14.39 ± 0.40	14.39 ± 0.39	0.569 (0.570)
Male (*n*, %)	1049, 49.0%	747, 48.4%	0.091 (0.763)
Maternal Ethnicity			0.794 (0.672)
White	1907, 89.2%	1423, 92.8%	
Black	36, 1.7%	29, 1.9%	
Other	195, 9.1%	82, 5.3%	
Paternal Ethnicity			0.2235 (0.889)
White	1996, 94.3%	1450, 94.6%	
Black	36, 1.7%	23, 1.5%	
Other	84, 4.0%	60, 3.9%	
Maternal Education			11.944 (0.063)
Professional qualification	251, 11.8%	201, 13.1%	
Bachelor degree	492, 23.1%	381, 24.8%	
Advanced diploma	344, 16.2%	266, 17.3%	
A levels or a BTEC national diploma	296, 13.9%	219, 14.2%	
NVQ or GNVQ	412, 19.4%	286, 18.6%	
O levels, GCSEs/CSEs or less	300, 14.1%	163, 10.6%	
None of the above	34, 1.6%	23, 1.5%	
Paternal Education			8.853 (0.182)
Professional qualification	363, 17.1%	282, 18.3%	
Bachelor degree	458, 21.5%	360, 23.4%	
Advanced diploma	272, 12.8%	203, 13.2%	
A levels or a BTEC national diploma	252, 11.8%	184, 12.0%	
NVQ or GNVQ	319, 15.0%	231, 15.0%	
O levels, GCSEs/CSEs or less	420, 19.7%	256, 16.6%	
None of the above	45, 2.1%	23, 1.5%	

Handedness			0.180 (0.672)
Left Handedness	228, 10.7%	157, 10.2%	
Right Handedness	1910, 89.3%	1386, 89.8%	
Imaging Center			3.710 (0.813)
London	260, 12.2%	190, 12.3%	
Nottingham	356, 16.7%	237, 15.4%	
Dublin	221, 10.3%	151, 9.8%	
Berlin	261, 12.2%	186, 12.1%	
Hamburg	260, 12.2%	207, 13.4%	
Mannheim	260, 12.2%	177, 11.5%	
Paris	260, 12.2%	206, 13.4%	
Dresden	260, 12.2%	189, 12.2%	

Percentages of missing values in all items are less than 1.0%.

* t-statistics for Age, and Chi-square statistics for all other variables, between adolescents selected in the analyses and all adolescents in the IMAGEN study. Two-sided p-values were reported

Supplementary Table 17. Demographics and baseline characteristics for participants in ABCD, IMAGEN and UKB study.

Variable	IMAGEN	ABCD	UKB
N	2138	11760	502409
Baseline Age (years; mean ± s.d.)	14.39 ± 0.40	9.91 ± 0.62	56.53 ± 8.09
Male (*n*, %)	1049, 49.0%	6146, 52.3%	229084, 45.6%
Ethnicity†			
White	1907, 89.2%	7463, 64.4%	454169, 90.9%
Black	36, 1.7%	1822, 15.7%	2872, 0.6%
Other	195, 9.1%	2297, 19.9%	42591, 8.5%
Parental Education* (Europe)			
Professional qualification	444, 20.9%		
Bachelor degree	589, 27.7%		
Advanced diploma	316, 14.8%		
A levels or a BTEC national diploma	259, 12.2%		
NVQ or GNVQ	310, 14.6%		
O levels, GCSEs/CSEs or less	198, 9.3%		
None of the above	13, 0.6%		
Parental Education* (US)			
Post graduate degree		4503, 38.3%	
Bachelor		2975, 25.3%	
Some college		2975, 25.3%	
High school diploma/graduate equivalency degree		1022, 8.7%	
Less than high school diploma		288, 2.5%	
Education (Europe)			
Professional qualification			25799, 5.2%
College or University degree			161128, 32.7%
A levels/AS levels or equivalent			55308, 11.2%
O levels, GCSEs or equivalent			105176, 21.4%
CSEs or equivalent			26885, 5.5%
NVQ/HND/HNC or equivalent			32724, 6.6%

None of the above			85259, 17.3%
Follow-up visit frequency	15-16y, 18-20y, 22-23y	Annual	Only baseline interview
Number of scans	3	2	data was used
Number of Imaging sites/scanners	8	31 (scanners)	22
Number of participants with genomic data	1982	11101	337199
Location	Europe	US	UK

† As the ethnicity group of participants is not available in IMAGEN, participants with both white parents were classed as White while those with both black parents were classed as Black.

* Parental education is defined as the highest educational level of parents.

Supplementary Methods

ABCD - Adolescent Brain and Cognitive Development

The ABCD Study is a landmark, longitudinal study of brain development, examining approximately 11,875 youth from 21 sites across the United States from age 9 to 10 for approximately ten years into young adulthood[1]. Data from the 3.0 (for baseline data collected between 2016-2018, ages 9-11) and latest 4.0 (for follow-up data collected between 2017-2020, ages 11-13) annual curated data releases of the ABCD project (https://abcdstudy.org/about/) were included. In our analysis, the ABCD data serves two main purposes. Firstly, it is used to estimate the developmental curve of total gray matter volume (GMV) in the reference population and group-specific GMV developmental curve. Secondly, it is used to examine the genetic influence on distinct neurodevelopmental patterns during adolescence through genome-wide association study (GWAS). A total of 11,811 participants (11,760 were included at baseline) aged 8.92 to 13.83 years old with neuroimaging data available was included in the study, 7,776 (65.84%) of which had two scans. Among the participants, 6170 (52.24%) are males and 5641 (47.76%) are females.

Structural neuroimaging data.
The ABCD neuroimaging data were obtained using 3T scanners (Siemens Prisma, General Electric MR750 and Philips Achieva dStream) with 32-channel head coil and high resolution T1-weighted structural magnetic resonance imaging (MRI). Both the methods and evaluations of these MRI images have been harmonized and optimized across all ABCD research sites[2,3]. The pre-processing processes were completed by the ABCD research teams according to the ABCD standard pipeline and protocol, with details described in the image processing paper[3]. The quality control (QC) procedure of the processed neuroimages was checked by the ABCD team both automatically and manually. Then regional morphometric structure evaluations were obtained using FreeSurfer 6.0 cross-sectional pipelines including cortical volumes from the FreeSurfer Desikan-Killiany (h.aparc) atlas, and subcortical volumes from the ASEG atlas. According to the FreeSurfer reconstruction QC measures (freesqc01), a total of 1,9576 scans including 11,811 participants passed the QC were included in the structural analyses. As there is more than one scanner in several sites, we controlled for scanner (*mri_info_deviceserialnumber* variable in *abcd_mri01* file) rather than site in the analysis. A total of 31 scanners was used in ABCD. Because most of the sample size of each scanner was large enough, regression method is able to substantially mitigate scanner effects.

Genetic data
The ABCD imputed genotype data were obtained from the public release 3.0. Imputation was performed using the Michigan Imputation Server with hrc.r1.1.2016 reference panel[4] and Eagle v2.3 phasing. We performed stringent QC standards by PLINK 1.90. Individuals with >10% missing rate and single-nucleotide polymorphisms (SNPs) with call rates < 95%, minor allele frequency < 0.1%, deviation from the Hardy-Weinberg equilibrium with P < 1E-10 were excluded from the analysis, yielding 11,101 participants and 5,020,358 SNPs. To ensure the homogeneity of the ABCD and IMAGEN population, we selected only ABCD subjects self-reporting ancestral origins as white, with 2,387 participants excluded. Considering that ABCD is oversampled for siblings and twins, and thereby has a nested structure, we randomly selected one participant within a family (the kinship relationship between participants was decided by genetically inferred zygosity status in *acspsw03* file). Finally, a total of 7,662 participants was included in the genetic analysis.

IMAGEN

The IMAGEN study is a significant multicenter genetic-neuroimaging study aimed to investigate the genetic and

neurobiological basis of individual variability in neurocognition, and determining their predictive value for the development of frequent psychiatric disorders[5]. The study recruited approximately 2,000 healthy Caucasian adolescents at age 14 (BL) from middle-class school across Europe in 8 sites (Berlin, Dresden, Dublin, Hamburg, London, Mannheim, Nottingham and Paris). Out of the recruited participants, 2,138 participants had neuroimaging data available. At each evaluation, participants had a structural MRI scan and a comprehensive assessment of their individual, social and family characteristics. Adolescents were followed up at the age of 16 years (FU1), 19 years (FU2) and 23 years old (FU3). However, neuroimaging data were only available at BL, FU2 and FU2. A total of 1,543 participants was included in the analysis. Among them, 747 (48.41%) are males and 796 (51.59%) are females.

Structural neuroimaging data

High-resolution T1-weighted images were obtained using 3T MRI systems based on the ADNI protocol (http://www.loni.ucla.edu/ADNI/Cores/index.shtml), from 4 different manufacturers (Siemens: 4 sites, Philips: 2 sites, General Electric: 1 site, and Bruker: 1 site). The scanning variables were specially chosen to be compatible with all scanners. The MR protocols and QC procedures of the IMAGEN study are described in Schumann et al.[6]. In brief, preprocessing is performed centrally using an automated pipeline that processes the continuously incoming data, and accounts for inter-site variability. In addition to the standard IMAGEN procedures, FreeSurfer (v6.0) Desikan-Killiany and ASEG atlas were used to extract regional brain morphology. 2,138 participants with 4,681 scans passed the QC. For consistency, cross-sectional rather than longitudinal FreeSurfer pipelines were used, including in the longitudinal dataset, as not every individual was measured on all three follow-ups. Therefore, data were preprocessed using cross-sectional FS pipelines and post-hoc analyses taking into account the within-individual correlations were conducted using mixed effect models. 21 individuals with GMV beyond 4 interquartile ranges (IQRs) in any left/right hemisphere regions were considered to be outliers and were excluded from the following analyses. To ensure a more accurate estimation of individual trajectories from linear mixed effect model, only 1,543 participants with at least two MRI scans were included in the study. Among them, 974 adolescents having a total of 3 scan and 569 adolescents having a total of 2 scans. As there is only one scanner manufacturer in every site, site was used as a covariate to control for potential scanner effect.

Genetic data and epigenetic data

Details of the genotyping and quality control are available in Desrivières et al.[7]. A same QC processing as the one in ABCD was performed using PLINK 1.90, where SNPs with call rates < 95%, minor allele frequency < 0.1%, deviation from the Hardy-Weinberg equilibrium with P < 1E-10 were excluded from the analysis. Then an imputation was conducted on the quality-controlled genetic data using the TOPMed imputation server (https://imputation.biodatacatalyst.nhlbi.nih.gov) with the HapMap3 reference panel[8]. After imputation, 5,966,316 SNPs were available for 1,982 IMAGEN sample. Among them, 1,398 were included in the analysis (644 for group 1, 694 for group 2 and 60 for group 3).

Epityping was conducted on DNA extracted from peripheral blood cells using the Infinium HumanMethylation450K BeadChip (Illumina) running on an Illumina HiScan System using the manufacturer's standard protocol. Pyrosequencing was carried out for technical validation and independent replication with a PSQ96 genetic sequencer using PyroMark Gold Q96 reagents (Qiagen, Valencia, Calif.) in accordance with the manufacturer's recommendations. DNAm beta values were normalized using Illumina GenomeStudio software. Quality control was performed by excluding CpGs with detection P < 0.01 and including samples had >98% sites with detection P < 0.01, yielding 372,582 CpGs and 1,329 samples. Among them, 446 were identified as group 1, 463 were identified as group 2 and 36 were identified as group3.

HCP - Human Connectome Project

The HCP consortium shared several large-scale cross-sectional neuroimaging datasets, which can be accessed through the HCP website (https://www.humanconnectome.org), including HCP Development (HCP-D) and HCP Young Adult (HCP-YA). The details on the inclusion and exclusion criteria of HCP YA were provided in the previous study[9]. HCP-YA sampled 300-400 healthy young adult sibships of average size 3-4, with most of these sibships including a MZ or DZ twin pair. A total of 1,113 participants from 457 unique families (including 170 dizygotic twins, 286 monozygotic twins, 576 non-twin siblings, and 25 non-sibling familial relations) aged 22-37 years old was included in the analysis with neuroimaging data available, comprising of 507 (45.55%) and 606 (54.45%) females. HCP-D is an extension of the HCP-YA study, specifically aimed at extending the coverage of HCP to a fuller lifespan[10]. It follows a comparable acquisition and recruitment protocol to HCP-Y but focuses on younger participants. A total of 652 participants aged 5.58-21.92 years old was included in the analysis with neuroimaging data available, comprising of 301(46.17%) males and 351 (53.83%) females.

Structural neuroimaging data

HCP-YA imaging data were acquired on a Siemens Skyra 3T scanner employing a 32-channel head coil with a customized SC72 gradient insert. HCP-D imaging data is conducted on a 3T Siemens Prisma scanners (Siemens, Erlangen, Germany). Participants 8-21 years old are scanned using the Siemens 32-channel Prisma head coil; a pediatric 32-channel head coil developed by Ceresensa (www.ceresensa.com) is used for 5-7 years old participants (see Harms et al. (under review)). Besides, contrast to HCP-YA, a slightly larger T1w voxel size (0.8mm) to allows some additional SNR margins, use of volumetric navigators for prospective motion correction, only one acquisition per modality to reduce scanning time, multi-echo acquisition for T1 (TE=1.8, 3.6, 5.4 and 7.2ms), slower TR (800ms) to allows maintenance of full Fourier k-space acquisition necessitated by the increased number of echoes were used for HCP-D participants. The preprocessing in HCP-D follows a similar procedure as in HCP-Y. Detailed protocols are available at https://www.humanconnectome.org. Minimally processed data was obtained directly from HCP and then be used to extract regional volumes using Freesurfer 6.0 Desikan-Killiany and ASEG atlas with cross-sectional pipelines.

PNC - Philadelphia Neuroimaging Cohort

PNC is a large-scale study of child development that incorporates rich multi-modal neuroimaging, genetics, and detailed clinical and cognitive phenotyping. The PNC data includes information from 9,498 children recruited from the Children's Hospital of Philadelphia care network. A sub-sample including approximately 1,000 healthy participants received multi-modal neuroimaging performed on a separate study visit at Penn[11]. A total of 1,587 participants aged 8.08-23.08 years old was included in the analysis with neuroimaging data available, comprising of 756 (47.64%) males and 831 (52.36%) females.

Structural neuroimaging data

All MRI scans were acquired at a single site, on a single 3T Siemens TIM Trio scanner with 32-channel head coil, in a short period of time that did not span any software or hardware upgrades as described previously[12]. In brief, receive coil shading was reduced by selecting the Siemens prescan normalize option, which corrects for B1 inhomogeneity based on a body coil reference scan. Image quality assessment was performed using visual inspection, which primarily focused on identifying excessive subject motion. Quality-controlled processed data was obtained directly from PNC and then be used to extract regional volumes using Freesurfer 6.0 Desikan-Killiany and ASEG atlas with cross-sectional pipelines.

UKB - UK Biobank

UKB study provides a large, comprehensive and ongoing dataset that includes both extensive phenotypic information as well as neuroimaging and genetics with over 500,000 participants aged 40-69 years across UK when recruited in 2006-2010[13]. In this study, we only used the baseline data to assess the genetic-predicted risk of delayed neurodevelopment on long-term outcomes. Socioeconomic, cognition and mental health outcomes, and structural brain morphology at mid-to-late adulthood were of interest. A total of 502,409 participants aged 37-73 years old was included in the analysis, comprising of 229084 (45.60%) males and 273325 (54.40%) females.

Structural neuroimaging data

Structural minimally processed T1-data was collected by the UK Biobank study with identical hardware and software in Manchester, Newcastle, and Reading, collected with a standard Siemens Skyra 3T scanner with a 32-channel head coil. Brain volumetric phenotypes were pre-processed by an imaging-pipeline developed and executed on behalf of UK Biobank[14]. Details of the imaging protocol can be found in an open-source document (https://biobank.ndph.ox.ac.uk/showcase/showcase/docs/brain_mri.pdf). Volumetric measures (mm^3) have been generated in each participant's native space. We used imaging-derived phenotypes of cortical and subcortical grey-matter volumes in regions of interest (UK Biobank category 192 & 190). A total of 43,103 participants balanced between sex was available with sMRI data across 22 sites.

Genetic data

Genotype data were available for all the initial participants in the UK Biobank cohort. Detailed genotyping and quality control procedures for the UK Biobank are available in a previous publication[15]. Additional QC procedures were conducted the same as described in ABCD and IMAGEN. We excluded SNPs with call rates < 95%, minor allele frequency < 0.1% or deviation from the Hardy-Weinberg equilibrium with $P < 1E{-}10$ and selected individuals that were estimated to have recent British ancestry and have no more than ten putative third-degree relatives in the kinship table, yielding 616,339 SNPs and 337,199 participants.

Supplementary Table 18. A sketch of data utilization in analysis.

	ABCD	IMAGEN	UKB	HCP	PNC
Group identification and characterization		√			
Estimation of group-specific GMV developmental curve	√	√		√ (HCP-D)	√
Estimation of peak total GMV in IMAGEN	√	√		√ (HCP-D / YA)	√
GWAS and GWAS validation	√		√		
EWAS and EWAS validation			√		
Long-term impacts of delayed neurodevelopment		√			

Supplementary Table 19. Neurocognition assessments in ABCD

Instrument	Variable	Description
Game of Dice Task[16]	gdt_scr_values_safe	Counts how many times participants selected a safe bet (bets on 3 or 4 dice faces)
	gdt_scr_values_risky	Counts how many times participants selected a risky bet (bets on 1 or 2 dice faces)

Delay Discounting Task[17]	ddis_scr_expr_mnrt_immcho / ddis_scr_expr_mnrt_allcho	mean latency of 'immediate' choices adjusted by mean latency of all choices
	ddis_scr_expr_mnrt_delaycho / ddis_scr_expr_mnrt_allcho	mean latency of 'delayed' choices adjusted by mean latency of all choices
NIH Tool Box[18]		
Picture Vocabulary Test	nihtbx_picvocab_uncorrected	a measure of general vocabulary knowledge
Flanker Inhibitory Control and Attention Test	nihtbx_flanker_uncorrected	a measure of executive function, specifically tapping inhibitory control and attention.
List Sorting Working Memory Test	nihtbx_list_uncorrected	a measure of episodic memory
Dimensional Change Card Sort Test	nihtbx_cardsort_uncorrected	a measure of executive function, specifically tapping cognitive flexibility
Pattern Comparison Processing Speed Test	nihtbx_pattern_uncorrected	a measure of speed of processing
Picture Sequence Memory Test	nihtbx_picture_uncorrected	a measure of episodic memory
Oral Reading Recognition Test	nihtbx_reading_uncorrected	a measure of reading decoding skill
Fluid Cognition	nihtbx_fluidcomp_uncorrected	A composite of Flanker, Dimensional Change Card Sort, Picture Sequence Memory, List Sorting and Pattern Compariso, which plays an important role in adapting to novel situations in everyday life
Crystallized Cognition	nihtbx_cryst_uncorrected	A composite of Picture Vocabulary and Reading Tests, which represents an accumulated store of verbal knowledge and skills
Total Cognition	nihtbx_totalcomp_uncorrected	A combination of fluid and crystallized composites

Supplementary Table 20. Environmental, neurocognition, behavioral, personal trait and mental health assessments in IMAGEN

Instruments	Variables	Descriptions
Environmental factors		
Life Events Questionnaire[19] (LEQ)	Family/Parents	Mean life-time frequency of events comprising parental divorce, parental discord, parental remarriage, parental alcohol abuse, family financial difficulties
	Accident / Illness	Mean life-time frequency of events comprising accident/illness, given medication by a physician, death in family, serious accident or illness
	Sexuality	Mean life-time frequency of events comprising falling in love, starting or ending a relationship, having first

		sexual experience, having a gay experience, pregnancy
	Autonomy	Mean life-time frequency of events related to independence including starting college, a hobby or new friends
	Deviance	Mean life-time frequency of events comprising getting in trouble, at school or the law, stealing
	Relocation	Mean life-time frequency of events related to change of school or residence
	Distress	Mean life-time frequency of events comprising face breaking out with pimples, starting to see a therapist, thinking about suicide, running away from home, getting poor grades at school, and gaining a lot of weight
	Overall valence	Mean life-time frequency of all measured events
Childhood Trauma Questionnaire[20] (CTQ)	Emotional abuse	Verbal assaults on a child's sense of worth or well-being or any humiliating or demeaning behavior directed toward a child by an adult or older person (CTQ_3+ CTQ_8+ CTQ_14+ CTQ_18+ CTQ_25)
	Physical abuse	Sexual contact or conduct between a child younger than 18 years of age and an adult or older person (CTQ_9+CTQ_11+CTQ_12+CTQ_15+CTQ_17)
	Sexual abuse	Bodily assaults on a child by an adult or older person that posed a risk of or resulted in injury. (CTQ_20+CTQ_21+CTQ_23+CTQ_24+CTQ_27)
	Emotional neglect	The failure of caretakers to meet children's basic emotional and psychological needs, including love, belonging, nurturance, and support (*CTQ_5+*CTQ_7+*CTQ_13+*CTQ_19+*CTQ_28)
	Physical neglect	The failure of caretakers to provide for a child's basic physical needs, including food, shelter, clothing, safety, and health care (CTQ_1+*CTQ_2+CTQ_4+CTQ_6+CTQ_26)
Development Well-being Assessment Interview (DAWBA)[21] - Family Stress Scale	Socioeconomics	Family stressors in socioeconomic/housing
	Work	Family stressors in work/pressure
	Health	Family stressors in illness
	Addiction	Family stressors in relationships/addition
DAWBA[21] - Family Life Questionnaire	Affirmation	Behaviors that the parent puts in place to support or help children in various situations or to show them approval and affection and refers to parent-child relationship (i.e. 'gets love and affection', 'praised and rewarded', 'gets help and support when stressed' and 'liked and respected')
	Discipline	Behaviors by parents in response to and intended to correct misbehavior by the children and it refers to punishment (i.e. 'physical punishment' and 'non-

		physical punishment')
	Rules	The ability to create coherent and shared family rules and to enforce them and it measures structure and organization within the family (i.e. 'clear rules' and 'consistently applied rules')
	Special allowance	Overprotection behaviors as opposed to lack of supervision (i.e. 'very protected' and 'Spends time alone')
Neurocognition		
Cambridge Neuropsychological Test Automated Battery[22] (CANTAB)		
Pattern recognition memory (PRM) - visual pattern recognition memory	Percent correct	Higher the percentage of correct trails, better the visual pattern recognition memory
Affective Go/No Go Task (AGN) - decision making	Total omissions for positive category	The AGN test assesses information processing biases for positive and negative stimuli. The subject is given a target category, and is asked to press the press pad when they see a word matching this category
	Total omissions for negative category	
Spatial Working Memory (SWM) - executive function	Between error	The number of times touching boxes that have been found to be empty and revisiting boxes which have already been found to contain a token
	Strategy	The number of times a subject begins a new search with a different box for 6- and 8-box problems only. A high score represents poor use of this strategy and a low score equates to effective use
Cambridge Gambling Test (CGT) - decision making	Delay aversion	Tendency to bet larger amounts when possible bet amounts are presented in descending order, calculated by subtracting risk taking measure from ascending trials from risk taking measure of descending trials
	Deliberation time	Mean latency from presentation of colored boxes to subject's choice of which color to bet on
	Overall proportion bet	Average proportion of the current point total (using nominal percentage) that subject is willing to risk on each gamble test trial
	Quality of decision making	Proportion of test trials on which the subject bets on the more likely outcome of the two choices
	Risk adjustment	Tendency to bet higher proportion of points when the large majority of boxes are the color chosen
	Risk taking	Mean proportion of the current points total that subject is willing to risk on trial for which they have chosen the more likely outcome

Rapid Visual Information Processing (RVP) - attention	A	A signal detection theory measure of target sensitivity
Intra-Extra Dimensional Set Shift (IED) - attentional flexibility	Total trials	The number of trials completed on all attempted stages
	Total trials adjusted	Attempts to compensate for the fact that subjects failing at any stage of the test have had less opportunity to complete trials. The adjustment adds 50 for each stage not attempted due to failure at an earlier stage
	PreED errors	The number of errors made prior to the extra-dimensional shift of the task. Errors are defined as instances when the subject fails to select the stimulus that is compatible with the current rule
	ED errors	The number of errors made in the extradimensional stage of the task. Errors committed at the reversal stage following the EDS stage are not included
	Total errors	The number of errors in all 9 stages
	Total errors adjusted	The adjustment adds 25 for each stage not attempted due to failure at an earlier stage
Monetary-Choice Questionnaire[23] (KIRBY) - impulsivity	Estimated K	A measure of delay discounting. Larger k values indicating greater delay discounting of value for the delayed options, indicating a higher level of impulsivity
	Estimated K for small LDRs	K value for small long delay reward
	Esitmated K for Medium LDRs	K value for small long delay reward
	Estimated K for Large LDRs	K value for small long delay reward
Stop Signal Task[24] (SST)	Go reaction time	Mean response time for ‘Go’ trials
	Go accuracy	Rate of correct "Go" trials
	Stop accuracy	Rate of correct "Stop" trials
	Stop signal reaction time	A measure indicative of the inhibitory control process with a lower value indicative of better such control
Behavioral risk factors		
DAWBA - Strengths and Difficulties Questionnaire[25] (SDQ)	Total difficulties score	Summing scores from all the scales except the prosocial scale
	Emotion problems score	i.e. "Many worries", "Nervous or clingy in new situations"
	Conduct problems score	i.e. "Often has temper tantrums or hot tempers", "Often lies or cheats"
	Hyperactivity score	i.e. "Restless, overactive", "Easily distracted, concentration wanders"
	Peer problems score	i.e. "Rather solitary, tends to play alone", "Picked on or bullied by other children"
	Prosocial score	i.e. "Considerate of other people's feelings", "Often

		volunteers to help others"
	Impact score	The sum of distress and impairment scores (i.e. interfere with home life/friendships/classroom learning/leisure activities by self-report)
European School Survey Project on Alcohol and Drugs[26] (ESPAD); Fagerstrom test for nicotine dependence[27] (FTND)	Lifetime smoking	"On how many occasions during your lifetime have you smoked cigarettes?"
	smoking last month	"How frequently have you smoked cigarettes during the last 30 days?"
	Whole life drink	"On how many occasions in your whole lifetime have you had any alcoholic beverage to drink?"
	Drink last year	"On how many occasions over the last 12 months have you had any alcoholic beverage to drink?"
	Drink last month	"On how many occasions over the last 30 days have you had any alcoholic beverage to drink?"
Personal trait		
NEO Five-Factor Inventory[28]	Neuroticism	Tend to accept negative emotions all the time, ignoring all the positive factors in life
	Extraversion	Like to engage with new people in social surroundings and are found to strengthen relationships
	Openness	Be open to new experiences and are often named under challenging personality
	Agreeableness	Be altruistic, sympathetic, and cooperate with everyone
	Conscientiousness	Be extensive with their duties and responsibilities
Temperament and Character Inventory[29] (TCI) - novelty seeking scale	Exploratory excitability	vs. stoic rigidity (i.e. "I prefer to start conversations, rather than waiting for others to talk to me")
	Impulsiveness	vs. reflection (i.e. "I often follow my instincts, hunches, or intuition without thinking through all the details")
	Extravagance	vs. reserve (i.e. "I prefer spending money rather than saving it")
	Disorderliness	vs. regimentation (i.e. "I lose my temper more quickly than most people")
	Total Novelty Seeking score	A sum of scores from the above four subscales
Mental symptoms		
Development Well-being Assessment Interview[21] (DAWBA)	Major Depression	A sum of thirty-five depression-related question scores by self-report
	ADHD (child)	A sum of three ADHD-related question scores by self-report (i.e. "Child says teachers/teachers complain", " Child thinks self-hyperactive")
	ADHD (parent)	A sum of thirty ADHD-related question scores by parent-rated

* indicates a reverse-coded preprocessing.

Supplementary Table 21. Socioeconomic, cognition and mental health assessments in UKB

Variables	Field ID	Descriptions
Socioeconomics		
Household income	738	Average total household income before tax, discretized by 18k, 40k, 52k and 100k
Indices of Multiple Deprivation (IMD) income score	26411 (England)<br>26428 (Scotland)<br>26418 (Wales)	Indices of Multiple Deprivation come from a UK government qualitative study of deprived areas in British local councils. The study is conducted separately in England, Scotland and Wales with different components. In general, IMD was consisted of scores from crime, education, employment, health, housing, income, access to services and physical environment.
IMD employment score	26412 (England)<br>26429 (Scotland)<br>26419 (Wales)	
IMD education score	26414 (England)<br>26431 (Scotland)<br>26421 (Wales)	
Job physical	816	Job involves heavy manual or physical work
Neurocognition		
Numeric memory test	4282	The participant was shown a 2-digit number to remember. The number then disappeared and after a short while they were asked to enter the number onto the screen. The number became one digit longer each time they remembered correctly (up to a maximum of 12 digits). We used maximum digits remembered correctly to measure numeric short-term memory
Trail making test	6348, 6350	The participant was presented with sets of digits/letters in circles scattered around the screen and asked to click on them sequentially according to a specific algorithm. We used the average time to complete numeric (trail #1) and alphanumeric path (trail #2) to measure visual attention and task switching
Tower rearranging test	21004	The participant was presented with an illustration of three pegs (towers) on which three differently-coloured hoops had been placed. The were then asked to indicate how many moves it would take to re-arrange the hoops into another specific position. We used the number of puzzles correct to measure executive functioning
Intelligence	20016	A sum of the number of correct answers given to the thirteen fluid intelligence questions
Education	6138	Recoded as: 4=College or University degree; 3=A levels/AS levels, NVQ or HND or HNC, other professional qualifications or equivalent; 2=O levels/GCSEs, CSEs or equivalent; 1=None of the above. The highest qualification was used
Mental health		

Depression	41270	Diagnosed as depressive episode (F32) or recurrent depressive disorder (F33)
Anxious	41270	Diagnosed as generalized anxiety disorder (F41.1)
Neuroticism score	20127	An externally derived summary score of neuroticisms, based on 12 neurotic behavior domains
Mood swing	1920	"Does your mood often go up and down?"
Miserableness	1930	"Do you ever feel 'just miserable' for no reason?"
Irritability	1940	"Are you an irritable person?"
Sensitivity	1950	"Are your feelings easily hurt?"
Fed-up feelings	1960	"Do you often feel 'fed-up'?"
Nervous feelings	1970	"Would you call yourself a nervous person?"
Suffer from 'nerves'	2010	"Do you suffer from 'nerves'?"
Tense	1990	"Would you call yourself tense or 'highly strung'?"
Worrier feelings	1980	"Are you a worrier?"
Loneliness	2020	"Do you often feel lonely?"
Guilty feelings	2030	"Are you often troubled by feelings of guilt?"
Risk taking	2040	"Would you describe yourself as someone who takes risks?"

Supplementary References

1 Karcher, N. R. & Barch, D. M. The ABCD study: understanding the development of risk for mental and physical health outcomes. *Neuropsychopharmacology* **46**, 131-142, doi:10.1038/s41386-020-0736-6 (2021).

2 Casey, B. J. *et al.* The Adolescent Brain Cognitive Development (ABCD) study: Imaging acquisition across 21 sites. *Dev Cogn Neurosci* **32**, 43-54, doi:10.1016/j.dcn.2018.03.001 (2018).

3 Hagler, D. J., Jr. *et al.* Image processing and analysis methods for the Adolescent Brain Cognitive Development Study. *Neuroimage* **202**, 116091, doi:10.1016/j.neuroimage.2019.116091 (2019).

4 McCarthy, S. *et al.* A reference panel of 64,976 haplotypes for genotype imputation. *Nat Genet* **48**, 1279-1283, doi:10.1038/ng.3643 (2016).

5 Mascarell Maricic, L. *et al.* The IMAGEN study: a decade of imaging genetics in adolescents. *Mol Psychiatry* **25**, 2648-2671, doi:10.1038/s41380-020-0822-5 (2020).

6 Schumann, G. *et al.* The IMAGEN study: reinforcement-related behaviour in normal brain function and psychopathology. *Mol Psychiatry* **15**, 1128-1139, doi:10.1038/mp.2010.4 (2010).

7 Desrivieres, S. *et al.* Single nucleotide polymorphism in the neuroplastin locus associates with cortical thickness and intellectual ability in adolescents. *Mol Psychiatry* **20**, 263-274, doi:10.1038/mp.2013.197 (2015).

8 International HapMap, C. *et al.* Integrating common and rare genetic variation in diverse human populations. *Nature* **467**, 52-58, doi:10.1038/nature09298 (2010).

9 Van Essen, D. C. *et al.* The Human Connectome Project: a data acquisition perspective. *Neuroimage* **62**, 2222-2231, doi:10.1016/j.neuroimage.2012.02.018 (2012).

10 Somerville, L. H. *et al.* The Lifespan Human Connectome Project in Development: A large-scale study of brain connectivity development in 5-21 year olds. *Neuroimage* **183**, 456-468, doi:10.1016/j.neuroimage.2018.08.050 (2018).

11 Satterthwaite, T. D. *et al.* The Philadelphia Neurodevelopmental Cohort: A publicly available resource for the study of normal and abnormal brain development in youth. *Neuroimage* **124**, 1115-1119, doi:10.1016/j.neuroimage.2015.03.056 (2016).

12 Satterthwaite, T. D. *et al.* Neuroimaging of the Philadelphia neurodevelopmental cohort. *Neuroimage* **86**, 544-553, doi:10.1016/j.neuroimage.2013.07.064 (2014).

13 Sudlow, C. *et al.* UK biobank: an open access resource for identifying the causes of a wide range of complex diseases of middle and old age. *PLoS Med* **12**, e1001779, doi:10.1371/journal.pmed.1001779 (2015).

14 Alfaro-Almagro, F. *et al.* Image processing and Quality Control for the first 10,000 brain imaging datasets from UK Biobank. *Neuroimage* **166**, 400-424, doi:10.1016/j.neuroimage.2017.10.034 (2018).

15 Bycroft, C. *et al.* The UK Biobank resource with deep phenotyping and genomic data. *Nature* **562**, 203-209, doi:10.1038/s41586-018-0579-z (2018).

16 Brand, M. *et al.* Decision-making deficits of korsakoff patients in a new gambling task with explicit rules: associations with executive functions. *Neuropsychology* **19**, 267-277, doi:10.1037/0894-4105.19.3.267 (2005).

17 Koffarnus, M. N. & Bickel, W. K. A 5-trial adjusting delay discounting task: accurate discount rates in less than one minute. *Exp Clin Psychopharmacol* **22**, 222-228, doi:10.1037/a0035973 (2014).

18 Hodes, R. J., Insel, T. R., Landis, S. C. & Research, N. I. H. B. f. N. The NIH toolbox: setting a standard for biomedical research. *Neurology* **80**, S1, doi:10.1212/WNL.0b013e3182872e90 (2013).

19 Accurso, E. C., Garland, A. F., Haine-Schlagel, R., Brookman-Frazee, L. & Baker-Ericzen, M. J. Factors contributing to reduced caregiver strain in a publicly-funded child mental health system. *J Emot Behav Disord* **23**, 131-143, doi:10.1177/1063426614532948 (2015).

20 Bernstein, D. P. *et al.* Initial reliability and validity of a new retrospective measure of child abuse and neglect. *Am J Psychiatry* **151**, 1132-1136, doi:10.1176/ajp.151.8.1132 (1994).

21 Goodman, R., Ford, T., Richards, H., Gatward, R. & Meltzer, H. The Development and Well-Being Assessment: description and initial validation of an integrated assessment of child and adolescent psychopathology. *J Child Psychol Psychiatry* **41**, 645-655 (2000).

22 Robbins, T. W. *et al.* Cambridge Neuropsychological Test Automated Battery (CANTAB): a factor analytic study of a large sample of normal elderly volunteers. *Dementia* **5**, 266-281, doi:10.1159/000106735 (1994).

23 Kirby, K. N., Petry, N. M. & Bickel, W. K. Heroin addicts have higher discount rates for delayed rewards than non-drug-using controls. *Journal of experimental psychology. General* **128**, 78-87, doi:10.1037//0096-3445.128.1.78 (1999).

24 Lappin, J. & Eriksen, C. Use of a delayed signal to stop a visual reaction-time response. *Journal of Experimental Psychology* **72**, 805-811, doi:10.1037/h0021266 (1966).

25 Goodman, R. The Strengths and Difficulties Questionnaire: a research note. *J Child Psychol Psychiatry* **38**, 581-586, doi:10.1111/j.1469-7610.1997.tb01545.x (1997).

26 Hibell, B. *et al.*

27 Heatherton, T. F., Kozlowski, L. T., Frecker, R. C. & Fagerström, K. O. The Fagerström Test for Nicotine Dependence: a revision of the Fagerström Tolerance Questionnaire. *British journal of addiction* **86**, 1119-1127, doi:10.1111/j.1360-0443.1991.tb01879.x (1991).

28 De Fruyt, F., Mervielde, I., Hoekstra, H. A. & Rolland, J. P. Assessing adolescents' personality with the NEO PI-R. *Assessment* **7**, 329-345, doi:10.1177/107319110000700403 (2000).

29 Cloninger, C. R. J. S. L., MO: Center for Psychobiology of Personality, Washington University. The temperament and character inventory-revised. (1999).
