# Supplementary material for: Investigating grey matter volumetric trajectories through the lifespan at the individual level
Source: Nat Commun. 2024 Jul 15;15:5954. doi: 10.1038/s41467-024-50305-0 (PMC11251262; doi:10.1038/s41467-024-50305-0)
Supplement: Supplementary file 3 — Reporting Summary [file 41467_2024_50305_MOESM3_ESM.pdf]

nature portfolio

Corresponding author(s): Xiaolei Lin
Barbara J. Sahakian
Jianfeng Feng

Last updated by author(s): Mar 10, 2024

Reporting Summary

Nature Portfolio wishes to improve the reproducibility of the work that we publish. This form provides structure for consistency and transparency in reporting. For further information on Nature Portfolio policies, see our Editorial Policies and the Editorial Policy Checklist.

Statistics

For all statistical analyses, confirm that the following items are present in the figure legend, table legend, main text, or Methods section.

n/a	Confirmed	
☐	☒	The exact sample size (*n*) for each experimental group/condition, given as a discrete number and unit of measurement
☐	☒	A statement on whether measurements were taken from distinct samples or whether the same sample was measured repeatedly
☐	☒	The statistical test(s) used AND whether they are one- or two-sided<br>*Only common tests should be described solely by name; describe more complex techniques in the Methods section.*
☐	☒	A description of all covariates tested
☐	☒	A description of any assumptions or corrections, such as tests of normality and adjustment for multiple comparisons
☐	☒	A full description of the statistical parameters including central tendency (e.g. means) or other basic estimates (e.g. regression coefficient) AND variation (e.g. standard deviation) or associated estimates of uncertainty (e.g. confidence intervals)
☐	☒	For null hypothesis testing, the test statistic (e.g. *F*, *t*, *r*) with confidence intervals, effect sizes, degrees of freedom and *P* value noted<br>*Give P values as exact values whenever suitable.*
☒	☐	For Bayesian analysis, information on the choice of priors and Markov chain Monte Carlo settings
☒	☐	For hierarchical and complex designs, identification of the appropriate level for tests and full reporting of outcomes
☐	☒	Estimates of effect sizes (e.g. Cohen's *d*, Pearson's *r*), indicating how they were calculated

Our web collection on statistics for biologists contains articles on many of the points above.

Software and code

Policy information about availability of computer code

Data collection	Data collections of ABCD, IMAGEN, PNC, HCP and UKB datasets were done centrally, not performed by us.
Data analysis	Freesurfer v6.0 was used to process the imaging data.<br>PLINK 1.90 was used to control genetic data quality and PLINK 2.0 was used to perform genome-wide association analysis and calculate gene-specific risk score.<br>PRSice v2.3.3 was used to calculate the polygenic risk score.<br>MAGMA v1.10 was used to perform gene-based GWAS.<br>R version 4.2.2 package:<br>lme4 1.1-31 was used to calculate individual annual growth rate for regional gray matter volumes and afex 1.2-1 was used to extract p-values for linear mixed effect model.<br>nlme 3.1-160 and splines 4.2.2 were used to fit individual gray matter trajectory curve.<br>effectsize 0.8.3 was used to calculate Cohen's d for group comparison.<br>lavaan 0.6-12 was used to perform mediation analysis.<br>The custom code was provided in the Github repository at https://github.com/abnmsry/Life-course-investigation-of-structural-neurodevelopment-at-the-individual-level

For manuscripts utilizing custom algorithms or software that are central to the research but not yet described in published literature, software must be made available to editors and reviewers. We strongly encourage code deposition in a community repository (e.g. GitHub). See the Nature Portfolio guidelines for submitting code & software for further information.

Data

Policy information about availability of data

All manuscripts must include a data availability statement. This statement should provide the following information, where applicable:

- Accession codes, unique identifiers, or web links for publicly available datasets
- A description of any restrictions on data availability
- For clinical datasets or third party data, please ensure that the statement adheres to our policy

The summary statistics of the GWAS for delayed brain development generated in this study has been deposited in the NHGRI-EBI Catalog of human GWAS database (https://www.ebi.ac.uk/gwas/) under GCP ID GCP000834 upon publication or is available at https://delayedneurodevelopment.page.link/29hQ. The raw ABCD, IMAGEN, HCP, PNC and UKB data are protected and are not available due to data privacy laws. However, access can be obtained upon application. ABCD data can be accessed at https://abcdstudy.org/; IMAGEN data can be accessed by email at https://imagen-project.org/; HCP data are available from: https://www.humanconnectome.org/; PNC data can be accessed from dbGaP: https://www.ncbi.nlm.nih.gov/projects/gap/cgi-bin/study.cgi?study_id=phs000607.v3.p2; and UKB data can be accessed at https://biobank.ndph.ox.ac.uk/. Public GWAS summary statistics of ADHD and ASD used in this study are available in the Psychiatric Genomics Consortium database of summary statistics at https://www.med.unc.edu/pgc/results-and-downloads, while public GWAS summary statistics of EA can be accessed at http://www.thessgac.org/data and public GWAS summary statistics of IQ can be accessed at https://ctg.cncr.nl/. All the data generated in this study are provided in the Supplementary Information and Source Data file. Source data are provided with this paper.

Research involving human participants, their data, or biological material

Policy information about studies with human participants or human data. See also policy information about sex, gender (identity/presentation), and sexual orientation and race, ethnicity and racism.

Reporting on sex and gender	We used the term sex throughout this manuscript. Sex was determind base on self-reports.<br>As heterogeneity in neurodevelopment exist in both males and females, we focused on the whole population and just use sex as a covariate to improve statistical power. Sex-stratified analysis has been performed regardless of the results on the whole population, which yielded similar results as those on the whole population, although some differences on differentiated neurocognition measurements were found between males and females.<br>All studies included in this manuscript have balanced sample sizes for male and female participants (Supplementary Table 15).
Reporting on race, ethnicity, or other socially relevant groupings	We only used the term ethnicity in the descriptive summary of participants characteristics. Ethnicity data was collected through self-report. As the ethnicity group of participants is not available in IMAGEN, participants with both white parents were classed as White while those with both black parents were classed as Black.
Population characteristics	For IMAGEN, healthy Caucasian adolescents a age 14 were recruited from middle-class school across Europe. 1,543 participants with at least two structural MRI scans were investigated in this study. 48.4% of them were males and 92.8% of them were classified as white. For ABCD, a total of 11,760 participants aged between 9 to 13 years (52.2% males) was obtained across 21 research sites across the USA. 7,662 uncorrelated adolescents reported as whites were included in this study with genetic data available. For HCP-YA, a total of 1,113 participants aged between 22 and 37 years (45.6% males) and for HCP-D, a total of 652 participants aged 5-22 years (46.2% males) were included in the analysis. For PNC, a total of 1,587 participants aged 8-23 years (47.6% males) were included in the analysis. For UKB, a total of 502,409 participants aged between 37 and 73 years (45.6% males) was obtained. Imaging data of 43,103 participants across 22 sites and genetic data of 337,199 participants were available and utilized in this study. A detailed inclusion of participants was described in Supplementary Methods and baseline population characteristics of the participants in the ABCD, IMAGEN, UKB were listed in Supplementary Table 17.
Recruitment	N/A (recruitment not part of this study)
Ethics oversight	Detailed ethic approval for ABCD can be found in the paper: Biomedical ethics and clinical oversight in multisite observational neuroimaging studies with children and adolescents: The ABCD experience. ABCD and HCP dataset was supported by the National Institutes of Health (NIH). The IMAGEN study was approved by local ethnics research committees at each research site: King's College London, University of Nottingham, Trinity College Dublin, University of Heidelberg, Technische Universitat Dresden, Commissariat a l'Energie Atomique et aux Energies Alternatives, and University Medical Center. UK Biobank has approval from the North West Multi-centre Research Ethics Committee as a Research Tissue Bank approval. Informed consent was sought from all participants and a parent/guardian of each participant if under 18 years in all studies .

Note that full information on the approval of the study protocol must also be provided in the manuscript.

Field-specific reporting

Please select the one below that is the best fit for your research. If you are not sure, read the appropriate sections before making your selection.

[x] Life sciences [ ] Behavioural & social sciences [ ] Ecological, evolutionary & environmental sciences

For a reference copy of the document with all sections, see nature.com/documents/nr-reporting-summary-flat.pdf

Life sciences study design

All studies must disclose on these points even when the disclosure is negative.

Sample size	No statistical methods were used to predetermine sample sizes. All currently available sample size were used in the study including 7,662 uncorrelated adolescents reported as whites in ABCD, 1,543 adolescents with at least two imaging scans in IMAGEN, 1,113 participants in HCP-YA, 652 participants in HCP-D, 1,587 participants in PNC and 502,409 participants in UKB.
Data exclusions	In all studies, participants failed the FreeSurfer reconstruction quality control and genetic data quality control (>10% missing rate) were excluded from the analysis. Moreover, to reduce bias in GWAS, all participants in ABCD reported as Black or Mixed were excluded and we randomly select one participant within a family. For IMAGEN, individuals with GMV beyond 4 IQR in any ROIs were considered to be outliers and were excluded from the following analyses. After applying the exclusion criteria, 1543 adolescents with at least two structural MRI scans from 14 to 23 years old were included in the analyses. For UKB, we selected individuals that were estimated to have recent British ancestry and have no more than ten putative third-degree relatives in the kinship table. For genetic data in ABCD, IMAGEN , UKB, we excluded single-nucleotide polymorphisms (SNPs) with call rates < 95%, minor allele frequency < 0.1%, deviation from the Hardy–Weinberg equilibrium with P < 1E-10. Details can also be found in Supplementary Methods.
Replication	No replication was conducted given the huge sample size of the present study.
Randomization	This is an observational study and no randomization was conducted. Nevertheless, covariates (e.g. research sites/scanners, sex and handedness) were included wherever suitable to control for potential confounding effects.
Blinding	This is an observational study and no cohort focus on any specific exposures or outcomes. So blinding was not needed.

Reporting for specific materials, systems and methods

We require information from authors about some types of materials, experimental systems and methods used in many studies. Here, indicate whether each material, system or method listed is relevant to your study. If you are not sure if a list item applies to your research, read the appropriate section before selecting a response.

Materials & experimental systems

n/a	Involved in the study	
☒	☐	Antibodies
☒	☐	Eukaryotic cell lines
☒	☐	Palaeontology and archaeology
☒	☐	Animals and other organisms
☒	☐	Clinical data
☒	☐	Dual use research of concern
☒	☐	Plants

Methods

n/a	Involved in the study	
☒	☐	ChIP-seq
☒	☐	Flow cytometry
☐	☒	MRI-based neuroimaging

Magnetic resonance imaging

Experimental design

Design type	Structural MRI
Design specifications	N/A
Behavioral performance measures	For ABCD, Game of Dice Task Youth, Delay Discounting Task and NIH Toolbox Summary Scores were obtained. For IMAGEN, personal traits were obtained from the NEO Five-Factor Inventory (NEO-FFI) and temperament and character inventory (TCI-R). Environmental burden were obtained from Pregnancy and Birth Questionnaire (PBQ), life-events questionnaire (LEQ), Childhood Trauma Questionnaire (CTQ), and Family Stress Scale and Family Life Questionnaire from development well-being assessment interview (DAWBA). Neurocognitive performances were obtained from Cambridge Neuropsychological Test Automated Battery (CANTAB) tests, Monetary-Choice Questionnaire (KIRBY) and Stop and Signal Task (SST) results. Behavior assessments were obtained from strengths and difficulties questionnaire (SDQ), European school survey project on alcohol and drugs (ESPAD), and Fagerstrom test for nicotine dependence (FTND). Mental health conditions were obtained from self-rated and parent-rated development well-being assessment interview (DAWBA). For UKB, household income, jobs involved in physical activity and Indices of Multiple Deprivation (IMD), fluid intelligence and the highest educational attainment, diagnosis of mental disorders (anxiety and depression), summary score of neuroticism and self-report mental symptom appearances were obtained. Detailed variables can also be found in Supplementary Methods.

Acquisition

Imaging type(s): T1-weighted structural imaging

Field strength: 3T

Sequence & imaging parameters: see ABCD, IMAGEN, HCP, PNC, UKB datasets official websites for details.

Area of acquisition: Whole brain

Diffusion MRI: ☐ Used ☒ Not used

Preprocessing

Preprocessing software: Freesurfer v6.0. FreeSurfer Desikan-Killiany (h.aparc) and ASEG atlases were used to extract cortical and subcortical regional GMV from quality-controlled processed data obtained directly from every study database. For UKB, public release neuroimaging data was obtained (category ID 190 and 192). Detailed preprocessing processes were described in Hagler et al. (2019) for ABCD, Schumann et al. (2010) for IMAGEN, https://www.humanconnectome.org for HCP, Satterthwaite et al. (2014) for PNC and https://biobank.ndph.ox.ac.uk/showcase/showcase/docs/brain_mri.pdf for UKB, as described in the Supplementary Methods.

Normalization: see above

Normalization template: fsaverage

Noise and artifact removal: see above

Volume censoring: see above

Statistical modeling & inference

Model type and settings: linear mixed effect model, generalized linear model

Effect(s) tested: For generalized linear model, t-tests were utilized to access statistical significance and derive t-statistics and corresponding two-sided p values adjusting for multiple comparisons. Cohen's d were derived from t-statistics.

Specify type of analysis: ☐ Whole brain ☒ ROI-based ☐ Both

Anatomical location(s): FreeSurfer Desikan-Killiany (h.aparc) and ASEG atlases were used to identify cortical and subcortical regions.

Statistic type for inference (See Eklund et al. 2016): N/A

Correction: False Discovery Rate (FDR) Benjamini-Hochberg (BH)

Models & analysis

n/a	Involved in the study
☒	☐ Functional and/or effective connectivity
☒	☐ Graph analysis
☒	☐ Multivariate modeling or predictive analysis
